# Sustained-input switches for transcription factors and microRNAs are central building blocks of eukaryotic gene circuits

**DOI:** 10.1186/gb-2013-14-8-r85

**Published:** 2013-08-23

**Authors:** Molly Megraw, Sayan Mukherjee, Uwe Ohler

**Affiliations:** 1 Institute for Genome Sciences and Policy, Duke University, 101 Science Drive, Durham, NC 27708, USA; 2 Department of Statistical Science, Duke University, Box 90251, Durham, NC 27708, USA; 3 Computer Science, Duke University, 308 Research Drive, Durham, NC 27708, USA; 4 Department of Mathematics, Duke University, 120 Science Drive, Durham, NC 27708, USA; 5 Department of Biostatistics and Bioinformatics, Duke University, 2424 Erwin Road, Durham, NC 27710, USA; 6 Berlin Institute for Medical Systems Biology, Max Delbrück Center for Molecular Medicine, Robert-Rössle-Strasse 10, 13125 Berlin, Germany; 7 Center for Genome Research and Biocomputing, Oregon State University, 2750 SW Campus Way, Corvallis, OR 97331, USA; 8 Department of Botany and Plant Pathology, Oregon State University, 2701 SW Campus Way, Corvallis, OR, USA

**Keywords:** gene regulation, microRNA, network motif, transcription factor

## Abstract

WaRSwap is a randomization algorithm that for the first time provides a practical network motif discovery method for large multi-layer networks, for example those that include transcription factors, microRNAs, and non-regulatory protein coding genes. The algorithm is applicable to systems with tens of thousands of genes, while accounting for critical aspects of biological networks, including self-loops, large hubs, and target rearrangements. We validate WaRSwap on a newly inferred regulatory network from *Arabidopsis thaliana*, and compare outcomes on published *Drosophila *and human networks. Specifically, sustained input switches are among the few over-represented circuits across this diverse set of eukaryotes.

## Background

The study of system-wide genetic circuit structure is useful to discover underlying patterns that are difficult to observe by looking at a few individual interactions in isolation. Studies of transcription factor (TF) networks in yeast have shown that even in unicellular organisms, there is a strong connection between overall regulatory architecture and TF dynamics [1-3]. Because additional components, such as non-coding RNAs known as microRNAs (miRNAs), have been shown to play crucial roles in gene regulatory networks of nearly every eukaryote [4-7], it would be desirable to identify and analyze patterns of regulatory architecture, especially patterns that include multiple regulatory entities with a variety of biological behaviors. For example, an early study used human gene-expression data to provide evidence that feed-back and feed-forward circuits involving both TFs and miRNAs are abundant in TF-miRNA interaction networks [8].

**Figure 1:**
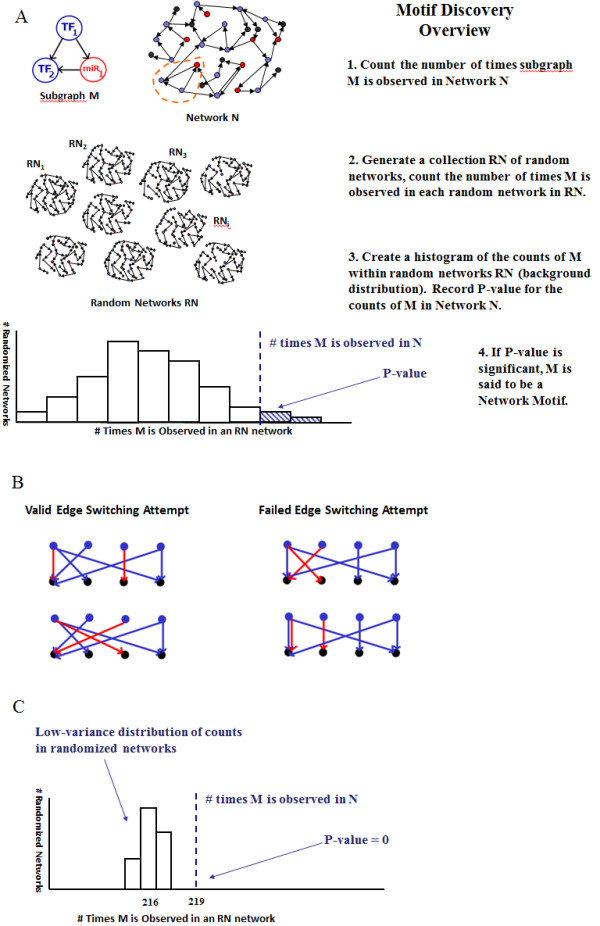
**An illustrative overview of network motif discovery, and two possible problems that may be encountered using the classic edge-switching method for network randomization**. **(A)** How network motif discovery works, illustrated using a subgraph of interest M. The method determines whether M is observed a significantly large number of times in an original network N, compared with randomized networks RN. If the *P*-value falls below a small pre-determined value, the subgraph count of M that is observed in original network N is considered to be significant, and therefore M is called a network motif for N. **(B**) Edge switching is a method for generating randomized networks RN in **(A)** from an original network N. It operates by repeatedly switching the endpoints of two edges. (Left) The two edges in red are selected for switching (top) and their endpoints are exchanged, resulting in a valid graph (bottom). (Right) An example of a failed edge switch: the two edges in red are selected for switching (top), but the exchange of endpoints (bottom) results in an invalid graph, in this case a graph with a double edge. **(C)** An example of a low-variance count distribution for a particular subgraph, which indicates that the subgraph is highly significant and therefore a motif. Low-variance count distributions (that is, those with small standard deviations) are one symptom of insufficiently randomized networks RN **(A)**, and can result from edge switching in large multi-layer networks.

One of the most relevant methods for identifying over-represented circuit structures within a larger network is called network motif discovery. This is a well-established statistical method for finding over-represented sub-structures within a larger network, and facilitates analysis of the small understandable components of networks, which are used repeatedly as building blocks for a system. The aim of motif discovery is to compare the frequency of particular sub-structures (such as a three-node feed-forward loop) in a given 'real-world' network with its frequency in randomized networks (Figure 1A). If the sub-structure appears much more often than in these randomized 'background' networks, it is said to be a network motif. Network motif finding algorithms have mainly used edge switching (Figure 1B), a fast and simple randomization method that preserves the exact in-degree (number of directed edges coming in) and out-degree (number of directed edges going out) of every node in a network [9,10]. However, concerns have been raised about the appropriateness of this method because edge switching can sample uniformly only in the limit of extraordinarily long sampling times, thus questionable results can be generated even for small TF-only networks on the order of several hundred nodes [11,12]. The problem underlying this difficulty is how to efficiently and uniformly sample a collection of bipartite graphs with source and target nodes of fixed in-degrees and out-degrees. To address this problem, a trade-off between uniformity and speed must be made when moving from near-regular graphs (low-variance degrees) to highly irregular graphs (networks such as those found in eukaryotes with large TF 'hubs' that have nodes of large in-degree or out-degree).

An algorithm that can handle arbitrary degree sequences (collections of node degrees, however uneven) was presented in a recent review [13]. This algorithm was more efficient than previous approximations, but was 'far from practical,' even for hundreds of nodes, and no code is available.

Two methods are currently available to address the case of near-regular graphs, a fully polynomial randomized approximation scheme (FPRAS) for undirected graphs [14], and a software program called Diaconis Monte Carlo Importance Sampling (DIA-MCIS) [15] which implements a rapid, statistically motivated heuristic based on sequential importance sampling [16]. Because most biological networks contain TF hubs, and therefore do not satisfy the regularity conditions required by these methods, they have not gained popular use within the computational biology community. A software implementation of the edge-switching technique, FANMOD (Fast Network Motif Detection) [17], has been applied to large predicted biological networks in humans to address a system with many genes and regulation of small RNAs [18,19]. Although some insight has been gained by such efforts, such studies have also reported that even if a large percentage of edges in the predicted biological network (up to 50% [19]) are replaced with falsified edges, the same network motif outcome is obtained. Although this could indicate robust results, it could also strongly suggest that the network motif outcome is an artifact of the sampling method in these networks. Thus, there is clearly a need for evaluation of appropriate methods to handle network motif analysis in such large multi-layer graph structures.

These theoretical limitations and concerns about the edge-swapping algorithm have a direct bearing on our understanding and interpretation of the actual important network motifs underlying many biological processes. However, because to date there is a complete absence of fast, publicly available algorithmic implementations that address these challenges, edge switching has remained the practical method of choice.

We hypothesized that an algorithm that treats the background graph-generation method in a way that respects the statistical goal of uniform draws, but also incorporates realistic biological network behavior, would reduce or eliminate the problems described above. Based on state-of-the-art methods from the probability literature and current biological knowledge of networks involving both TFs and miRNAs, we designed a new hybrid algorithm, WaRSwap (Weighted-and-Reverse-Swap), which respects uniformity constraints even for graphs with highly uneven in-degree and out-degree distributions (large source and target hubs), and performs efficiently on graphs with tens of thousands of nodes and hundreds of thousands of edges. We compared WaRSwap with current methods on theoretically motivated test examples, as well as actual TF-miRNA-gene networks generated with a variety of biological constraints (binding stringencies) in a large eukaryotic genome, *Arabidopsis thaliana*. For a realistic comparison on large graphs approaching genome-scale networks, the *Arabidopsis *genome was chosen for its low-noise TF and miRNA target prediction characteristics (small intergenic regions, near-complementary miRNA targeting) and large gene set (around 30,000 genes, similar to the number of human genes). We found that the method facilitated discovery of biologically relevant patterns in the gene-circuitry structure and generation of hypotheses in a large genetic system involving both TFs and non-coding RNAs. The arrival of the WaRSwap algorithm is timely, with increasingly available experimental data and interest in other large and complex genetic systems including human cell lines.

Our validation of WaRSwap in *Arabidopsis *identified prevalent autoregulatory gene circuits long associated with developmental patterning. Among the motifs containing both TFs and miRNAs, the miRNA-mediated feed-forward loop was a predominant theme, but our results suggest that this pulse-generating circuit may be specific to tightly regulated TF and miRNA binding. We also identified a gene circuit capable of creating a sustained-input switch for a downstream TF or miRNA that is robustly embedded into the structure of our biological network in *Arabidopsis*. We compared these outcomes with applications of WaRSwap in published *Drosophila *and human regulatory networks, and made the striking finding that sustained-input switches are among the few over-represented circuits in these networks as well. Our analysis supports the hypothesis that sub-circuits associated with development are dominant across large multicellular genetic systems because of the need for reliable spatiotemporal pattern formation in complex organisms.

## Results

The goal of network motif discovery is to identify small over-represented subgraphs within a larger graph structure. This goal is achieved by counting the number of a particular subgraph present in the biological network of interest, and then comparing this number with the distribution of counts for this same subgraph over a number of randomized networks (using a *P*-value or z-score as an indicator of how extreme the value is compared with the distribution of values from randomized networks; see Figure 1A for a visual illustration). To obtain a meaningful outcome, randomized networks must be produced in a way that 1) samples as uniformly as possible from the set of all obtainable random networks (that is, it does not unjustifiably produce certain random networks more often others), 2) reflects the biological properties of the actual network, and 3) is time-efficient and practical.

Edge switching operates by taking the original biological network, selecting two directed edges within that network, and switching their endpoints if possible (Figure 1B), then repeating this 'select two edges and swap endpoints' routine for a defined number of attempts. Network motif discovery using edge switching has been shown to be valuable in bacterial and yeast networks, despite the theoretical caveats of the method. However, because the networks of higher eukaryotes are often larger (tens of thousands of nodes, as opposed to several hundred nodes) and contain multiple layers of regulation (TFs and non-coding RNAs), we discovered that these caveats can impede analyses of these networks.

We identified three primary difficulties when applying edge switching, as implemented in FANMOD, on networks of the desired size; in this case, the tens of thousands of nodes and hundreds of thousands of edges in an *Arabidopsis *network (Table 1). Using the recommended settings and three-node motif searches, we found an unexpectedly large number of motifs with a high z-score and small *P*-value, which did not greatly narrow the focus to a much smaller set of subgraphs. This result alone is not worrisome because it is possible that, biologically, all of the motifs are significant. However, upon close inspection of the resulting background histogram of many 'significant' motifs, we found the standard deviation of the number of randomized motifs to be very small (Figure 1C), clearly indicating inadequate sampling. Finally, upon tracing the execution of the algorithm, we discovered a large number of failed switches; that is, an edge swap between two adjacent nodes that results in multiple edges and therefore cannot be performed (Figure 1B). In our networks, an estimated 50% of the motifs discovered with edge switching exhibited the problems above (Table 1), which is the problem our algorithm addresses.

**Table 1:** Graph size, percentage of failed FANMOD edge switches, and motif count standard deviations for the 12 biological networks considered in the analysis.^a^

FNR (TF binding sites)	deltaG (miRNA targets), n	Nodes, n	Edges, n	FANMOD failed switches, %	Standard deviation	
					WaRSwap count, n	FANMOD count, n
0.2	60	27,289	463,239	96	1,241,486	3,638
	70	27,281	462,242	96	18,154	3,626
	80	27,257	461,146	96	17,512	3,426
0.4	60	25,835	255,485	89	713,360	3,739
	70	25,791	254,488	89	10,484	3,726
	80	25,734	253,392	90	10,244	3,588
0.6	60	21,034	107,776	73	255,576	1,791
	70	20,883	106,779	73	3,751	1,759
	80	20,653	105,681	74	3,658	1,637
0.8	60	12,366	36,301	57	58,094	434
	70	12,009	35,303	58	841	420
	80	11,506	34,203	59	825	392

Abbreviations: FANMOD, Fast Network Motif Detection; FNR, False-negative rate, miRNA, MicroRNA; TF, Transcription factor; WaRSwap, Weighted-and-Reverse-Swap.

^a^For each biological network, there were 2,500 random subgraphs generated using the WaRSwap and FANMOD algorithms.

We hypothesized that these problems were symptomatic of an inadequate exploration of the random graph search space for large networks with highly uneven degree distributions, such as the biological networks typical of multicellular eukaryotes. *Arabidopsis *is advantageous for graph construction because of its straightforward miRNA target identification and short intergenic regions, which limit the *cis*-element search space. Despite this, the number of *Arabidopsis *genes (around 30,000) also far exceeds that of single-cellular organisms, and is similar to the size of the human gene set. Given this large genome size but relatively simple architecture, this system is ideal for methodological development. We designed a new algorithm using high-confidence *Arabidopsis *networks to test and address the algorithmic requirements of motif finding in large eukaryotic gene networks with multiple layers of regulation.

### WaRSwap: an algorithm to identify over-represented TF-miRNA-gene circuits in large eukaryotic genomes

#### WaRSwap design addresses motif discovery in large multi-layer networks

**Figure 2:**
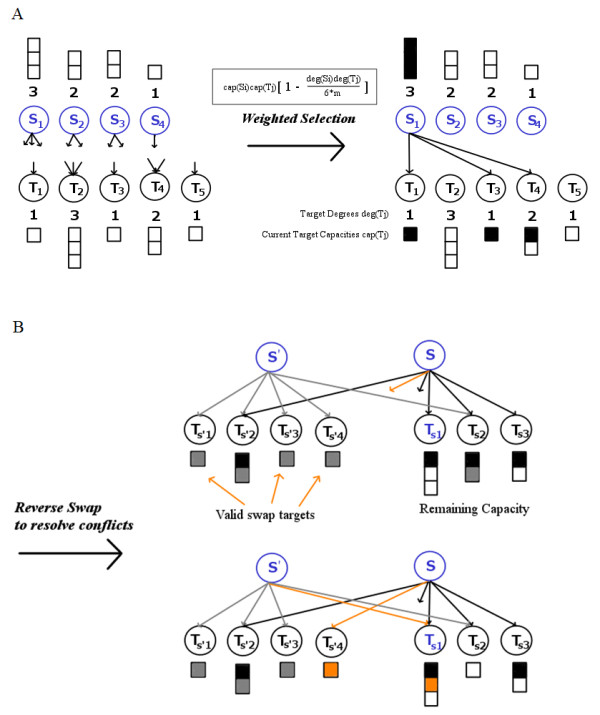
**Weighted-and-Reverse-Swap (WaRSwap). (A) WaRSwap weighted vertex selection step: source vertices are placed in descending degree order, and sources 'select' their targets for a directed-edge pairing of source and target**. The target vertices are selected by a source via weighted random sampling without replacement, with each target being assigned an 'attraction' weight that is proportional to the current source and target capacity, but reduced by a factor that prevents overly frequent selection of large-degree source-target pairs. In this way, over-selection of hub to hub connections is avoided. In the weighting formula, *m *is the number of required edges in the graph. **(B)** WaRSwap reverse-swap step: if the algorithm reaches a state where a source S has undistributed edges to place (such as the orange edge in the top illustration) but no valid targets, S will also have unfilled capacity for at least one target (shown here as T_S1_) for which a valid edge exchange can be determined. The method identifies a corresponding target (shown here as T_S'4_) from a previously visited source S', such that the unplaced edge of S can connect to this target, and the previously placed (orange) edge from S' to T_S'4 _can be moved to fill the open capacity of the S' target, T_S1_.

The Core Method of the WaRSwap sampling algorithm combines two main strategies to achieve fast, near-uniform sampling on graphs with both regular and highly uneven degree distributions. (1) in weighted sampling the algorithm uses dynamically corrected weighting at each vertex selection step to place an edge between two vertices according to their current degrees and total required degrees [14]; this strategy avoids over-selection of hub-hub connections. (2) Reverse swapping is then carried out if necessary. This distributes edges from the large source hubs first; if/when the algorithm reaches a state where no valid edge can be created, it backtracks to find a valid swap with an already-placed edge, so that the method can proceed instead of discarding the sample. These two core strategies are illustrated in Figure 2.

**Figure 3:**
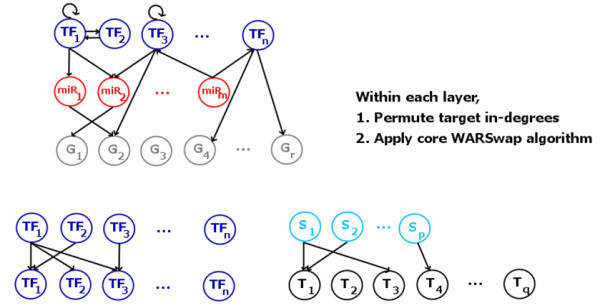
**Weighted-and-Reverse-Swap (WaRSwap) treats the biological network as a composition of five possible source and target layers**, The only layer that has self-loops is the TF→TF layer. Each layer is randomized by first permuting target in-degrees, then applying the core WaRSwap algorithm. The result is a randomized network that preserves target in-degree distribution with respect to each source-target type.

The WaRSwap Core Method is incorporated into an algorithm with the capacity to handle multi-layered graph structures, which treats the interactions between each type of node in the network according to their biological constraints (Figure 3). In a biological network with three different types of nodes, that is, TFs, miRNAs, and non-TF protein-coding genes (referred to hereafter simply as 'genes'), there are five biologically different types of targeting interactions that occur (represented in a graph by directed edges): TF→TF, TF→miRNA, TF→gene, miRNA→TF, and miRNA→gene. Among these five graph layers, there are effectively two different types of source-target interaction (see Figure 3): layers where sources can target themselves (TF→ TF) and layers where this cannot happen (all others). Thus, self-loops are handled in a natural way by the method; they are simply treated as a directed edge from a source to a target in the TF→TF layer of the network, and the core algorithm is applied to this layer exactly as to all others. Because miRNA and TF target sites may be gained and lost during evolution, we specifically designed WaRSwap to preserve the in-degree distribution for each layer rather than the exact in-degrees. Figure 3 shows how this is achieved using WaRSwap: for each source-target layer in the network, the in-degrees are first permuted, and the core sampling method described above is then applied to the layer. The WaRSwap algorithm is presented in full, step-wise detail in the Methods section.

#### WaRSwap handles the highly uneven degree distributions of biological networks

**Figure 4:**
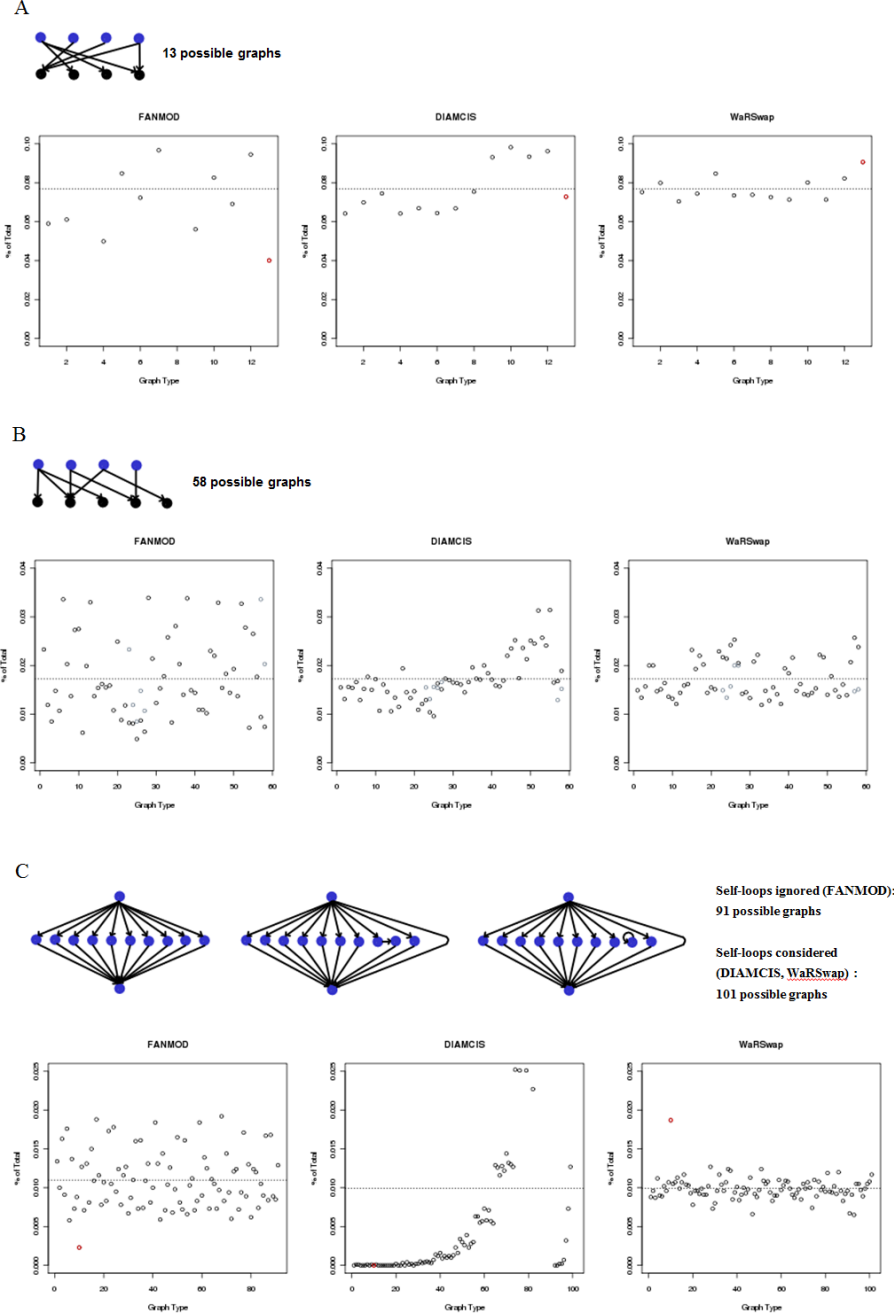
**The methods Fast Network Motif Detection (FANMOD) (involves edge switching), Diaconis Monte Carlo Importance Sampling (DIA-MCIS) (involves importance sampling), and Weighted-and-Reverse-Swap (WaRSwap) were used to produce 10,000 randomized graphs for each of three examples**. The resulting graphs were tabulated according to their unique numerical representation (simply shown as integers on the × axis). The percentage of each graph produced is plotted on the Y axis, and the horizontal dotted line in each plot displays the percentage representing perfect uniformity. **(A)**. Because there are 13 possible graphs using the same in-degrees and out-degrees as the example shown, an algorithm that samples the graph space uniformly would produce each possible type of graph exactly 1/13 of the time. The values shown in red correspond to the single possible graph with no connections between the source vertex of out-degree 3 and the target vertex of in-degree 3, which is the graph instance displayed here. **(B)** An example with 58 possible graphs. **(C)** The graph instance at the left is a canonical example of an extremely uneven degree distribution [20]. In this example, all vertices are blue because they are represented here as being both sources and targets (that is, as transcription factors). Graphs with these particular in-degrees and out-degrees can have three different types of topologies, as shown here. The instance at the left is the only possible one with no hub to hub connection, and the sampling outcome for this graph is highlighted in red on the plots for each method. The other two topologies shown correspond to 90 and 10 possible outcomes, respectively. Thus, methods that consider self-loops (for example, Diaconis Monte Carlo Importance Sampling (DIA-MCIS) and Weighted-and-Reverse-Swap (WaRSwap)) have a total of 101 possible labeled outcomes, whereas those that do not consider these (for example, Fast Network Motif Detection (FANMOD)) have a total of 91 possible labeled outcomes. Graphs containing self-loops correspond to the 10 rightmost X-axis labels in the plots for DIA-MCIS and WaRSwap.

The theoretical underpinnings of the WaRSwap Core Method are designed to handle large graphs that might have highly irregular degree distributions. However, all algorithms must make some tradeoffs in uniformity when handling extreme cases. To evaluate the relative ability of the Core Method to sample graph spaces uniformly in a practical way, we used canonical examples to directly compare its performance with two other methods: FANMOD [17], a commonly used implementation of the edge-switching algorithm, and DIA-MCIS [12,15], an importance sampling method intended to address the shortcomings of edge-switching in TF networks. Figure 4 displays a comparison on three graphs of increasing vertex number, for which it is still possible to enumerate the total number of graphs with the same fixed in-degrees and out-degrees. Even on this progression of tiny examples, the number of possible outcomes increased rapidly (as discussed in the Introduction, there is no closed formula for determining the number of such graphs). Each algorithm was applied to the given graph (Figure 4) (in the case of WaRSwap and DIA-MCIS, the methods started from the given degree sequences defined by the graph rather than the graph itself) to generate 10,000 random graphs.

We found that WaRSwap behaved in accordance with its theoretical expectation. On examples without extreme degree distributions (Figure 4a, b), the Core Method tightly clustered the results about the uniform probability value compared with FANMOD, while on extreme examples, such as the canonical 'large-hub' graph in Figure 4c[20], this finding also held true. We also found that whereas the other methods tended to undersample graphs with no hub-hub connections, WaRSwap slightly oversampled (as in the case of Figure 4c), although not to an extent greater than FANMOD's deviation for many sampled instances. WaRSwap's weighting correction parameter can be specifically tuned; here, it was simply fixed at the smallest definable integer value for all real-world biological networks that we considered in our study (detailed in Methods), and it was not chosen to enhance performance on these specific examples in any way. Interestingly, DIA-MCIS generally achieved its goal of sampling more uniformly than FANMOD on these small examples where degree distributions were relatively even (Figure 4a, b); however, the method went awry in other cases (Figure 4c) and vastly undersampled the graphs with no hub-hub connections. Additional degree variation and sampling uniformity comparison examples are provided (see Additional file 1).

The construction of WaRSwap accomplishes three design goals for a network motif discovery algorithm in large multi-layer networks involving both TFs and miRNAs. 1) Within each biological network layer, WaRSwap samples uniformly on both near-regular graphs and on canonically troublesome extreme cases with large source and target hubs. 2) WaRSwap accounts for important biological properties of graphs involving both TFs and miRNAs. TFs target themselves in real biological networks (in fact, self-loops are an important theme in developmental networks [21]), and TF binding sites as miRNA target sites are not static entities, but rather may be gained and lost over time [22]. 3) WaRSwap results in a valid graph after visiting each source node once (the result of distributing high weights first, then performing exchanges to escape invalid states), allowing the method to sample quickly enough to be practical on large graphs (see Methods).

### WaRSwap identifies nested subsets of circuits identified by edge swapping

We first performed a stability analysis to determine a number of samples for which WaRSwap provided highly stable *P*-value output over all three-node subgraphs for the large biological networks under evaluation. We found that having 2,500 random graphs per background distribution was more than sufficient. We then compared WaRSwap and FANMOD using a set of large biological networks (Table 1), which resulted from different TF binding-site and miRNA target stringencies. In particular, we constructed networks with four different TF binding thresholds, from an extremely stringent matching requirement for each TF binding domain, as represented by a positional weight matrix (PWM) with a false-negative rate (FNR) of 80%, to a strong but less stringent requirement (FNR of 20%). For each of these TF binding thresholds, we constructed networks with three different miRNA target stringencies as represented by a minimum deltaG (dG) free energy threshold (from most to least stringent, dG ≥ 80, dG ≥ 70, dG ≥ 60). WaRSwap and FANMOD were applied to each network, and three-node subgraphs were considered (these applications omitted self-loops from the networks to allow for direct comparison). For each method, we considered a three-node subgraph to be a motif if it was over-represented in one or more of the biological networks with the simultaneous requirements of a *P*-value of < 0.01, a z-score of > 2.0, and a standard deviation (SD) of > 1 (reported in terms of number of observations).

Under these strict requirements, we found that WaRSwap network motifs were a near-nested subset of the corresponding results using standard edge switching (FANMOD) (see Additional file 1: Figure S1). This result was true across all of the datasets in our study, even when different upstream region lengths were considered (see Additional file 1: Figure S2). When considering three-node circuits where all nodes interact, the resulting sets were strictly nested (Additional file 1: Figure S3). WaRSwap loosened the requirement from preserving exact target node in-degrees in randomized networks to preserving only in-degree distribution. It was therefore striking that this more extensive randomization led to fewer network motifs at a given *P*-value cutoff, and in particular, did not lead to any additional motifs not suggested by edge swapping.

Evidence from these comparison studies strongly supports our hypothesis that for large graphs, symptoms indicative of spuriously 'discovered' network motifs derive from the fact that edge switching does not appropriately explore the space of possible random graphs. Rather than being 'conservative', edge switching can produce misleading results because the null distribution of subgraph counts is actually too narrow. In many cases, this produces sub-circuit characteristics in the randomized graphs that are unrepresentative of the original graph (and thus, these circuits have a tendency to spuriously 'stand out' with a high *P*-value). WaRSwap produces randomized graphs that better explore the space of all possible such graphs; on average it yields larger SDs for all subgraphs considered (see Table 1) and relatively smooth *P*-values as the network parameters are varied (Figure 6). For a given number of background graphs, WaRSwap thus provides a more conservative and trustworthy result for large multi-layer networks. This greatly reduces concern over previously reported difficulties in determining whether a subgraph is a network motif or simply a robust artifact that is due to the limitations of edge switching in large multi-layer networks [18].

### Developmental circuits are building blocks of the *Arabidopsis *gene network

**Figure 5:**
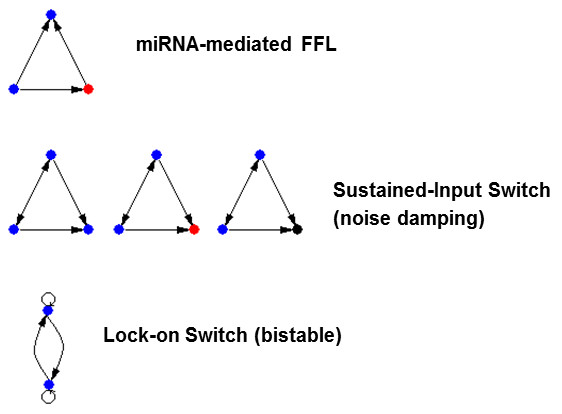
**Three categories of network motifs were discovered in our *Arabidopsis *networks by Weighted-and-Reverse-Swap (WaRSwap)**. 1) The microRNA (miRNA)-mediated feed-forward loop; 2) sustained-input (or noise-damping) switches for transcription factors (TFs), miRNAs, and genes; and 3) the lock-on bi-stable switch for TFs.

Because WaRSwap allowed us to identify a small set of the most confident network motifs, we examined the classes of the three-node motif topologies for several cases: one-node motifs without self-loops (that is, they are self-loops themselves), and two-node or three-node motifs 'decorated' by self-loops on the TF nodes. Three over-represented topologies emerged (Figure 5): 1) regulating feed-back motifs that can serve as sustained-input switches (or sustained noise repressors), 2) a two-node bi-stable switch, and 3) the miRNA-mediated feed-forward loop (FFL). Additionally, self-loops themselves were over-represented, and a wide variety of decorated three-node subgraphs were found to be motifs when the decorated and self-loopless subgraphs were considered separately (see Methods).

The FFL is generally common in TF networks of both unicellular and multicellular organisms, and two of the most abundant types exhibit temporal control functions such as pulse generation, response acceleration, and filtering of noisy input signals [21]. The miRNA-mediated FFL was originally identified in human gene-expression studies as being abundant [8], and has been universally noted in subsequent network motif finding applications of edge switching to a variety of organisms. However, regulating feed-back motifs have not been noted. Interestingly, in this study we observed two types of regulating feed-back motifs, both of which are associated with development in multicellular organisms [21]: a bi-stable switch, known as a 'lock-on' motif, which ensures expression of two TFs after either one reaches a crucial threshold, and a 'sustained-input' motif that ensures stable expression of a downstream gene or miRNA in cases where both regulating TFs serve as activators. The sustained-input switch can also have a noise-repression role when both regulating TFs serve as repressors, as an input signal to either regulating TF is damped, shutting down input to the downstream gene.

All of these motifs (including the lock-on motif, whose observation is enabled by WaRSwap's ability to test networks containing self-loops) serve functions consistent with the need for a multicellular organism with a large and complex genetic system to achieve and maintain spatial patterning integral to development. Regulating feed-back motifs, including the sustained-input switches and noise repressors, are known to be crucial components of canonical plant sub-networks that serve a well-studied purpose in plant development. For example, the plant TFs AP1 and LFY are known to upregulate both each other and their mutual target PI as part of the cell-fate determination process during flower development [23]. Mutual feed-back switches with and without self-loops are central to hormonal control of root meristem cell differentiation (such as SHY2 and Auxin mutual repression) [24] and to stem cell maintenance in shoot apical meristem (such as CLV3 and WUS) [25,26]. Our study identified thirteen instances of miRNA-mediated FFLs at the highest level of binding stringency, eight two-node bi-stable switches, and several hundred sustained-input switches. All of the entities involved in these circuits have experimental support for gene expression within the same *Arabidopsis *root cell type. The circuits have been made available for further study (see Additional file 2).

In order to provide additional insight into how these motifs may be specifically contributing to developmental programs, we performed an analysis using the GOStat toolset [27] to identify statistically significantly over-represented Gene Ontology (GO) terms in the set of *Arabidopsis *genes (including miRNAs) that we identified as being both 1) present in at least one WaRSwap-identified motif and 2) co-expressed in the same *Arabidopsis *tissue as all other genes participating in the same motif. In this gene set, 19 over-represented GO terms (with respect to all *Arabidopsis *genes as a control) were identified. Functional categories are generally related to transcriptional and post-transcriptional gene regulation, but specific categories include epigenetic control and regulation of developmental processes. Of the five categories related to development (these terms were associated with miRNAs 164c and 167a), the specific functional terms all highlight reproductive structure development, including specification of floral organ number. This is supportive of the concept that the sustained-input switches in the *Arabidopsis *system take on roles that include morphological patterning. We have made the gene list and GOStat analysis output available (see Additional file 3).

### FFLs are significant only under stringent binding conditions

**Figure 6:**
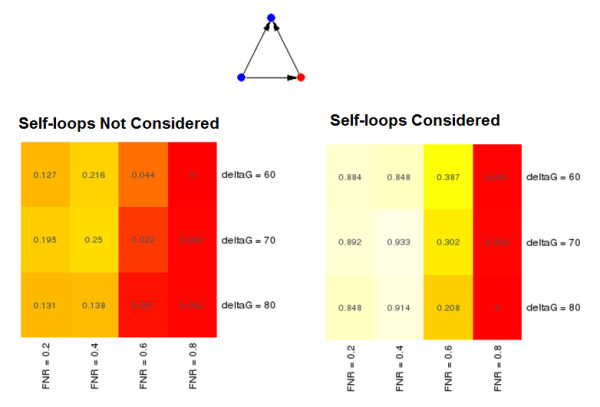
***P*-values for the miRNA-mediated feed-forward loop (FFL) over the 12 *Arabidopsis *networks evaluated by Weighted-and-Reverse-Swap (WaRSwap)**. (Left) Self-loops in the networks were not considered; (right) self-loops were taken into account, so that miRNA-mediated FFLs with one or more transcription factor (TF) self-loops were considered to be separate subgraphs in the analysis.

In our analysis, we examined a large variety of parameterized networks for the purpose of understanding the behavior of a network motif discovery algorithm in response to graded differences in network construction (Table 1). In running WaRSwap over this grid of 12 networks, we found that the use of different TF binding requirements resulted in clear differences in the network motifs identified and in their quantitative significance values. When different miRNA target stringencies were used, these differences were much less pronounced. However, we still observed a clear trend for the miRNA-mediated FFL, which became even more predominant when three-node subgraphs with all possible TF self-loop decorations were considered separate entities (Figure 6).

We found that in addition to miRNA-mediated FFLs becoming increasingly significant with greater TF binding-stringency cutoffs, there was also a tendency toward increasing significance at more stringent hybridization energy requirements for miRNA targeting. Therefore, in the networks under examination, it appears that FFLs are over-represented only under the strictest of binding conditions. WaRSwap enables this trend to be discovered via analysis of multiple parameterized networks, by providing adequate randomization in the larger networks with looser binding-threshold requirements. This finding is consistent with the hypothesis that the temporal control functions of FFLs (for example, pulse generation of TFs with many downstream effectors) are common only under tightly regulated binding conditions that ensure FFL regulation occurs in the proper context of the larger network.

### Sustained-input switches unify network motif outcomes in *Arabidopsis*, *Drosophila*, and human TF-miRNA-gene regulatory networks

While generating high-confidence genome-scale networks in animal organisms is a challenge compared with generating them in *Arabidopsis*, because the TF binding-site and miRNA target predictions are considerably lower confidence endeavors in these systems (larger intergenic distances and more complex miRNA target patterns), we wanted to compare the motif outcome of WaRSwap with that of FANMOD in other eukaryotes. We chose *Drosophila *and human networks provided by the modENCODE and ENCODE consortiums, as each of these has published a sizeable network with some experimental support that involves both TFs and miRNAs [28,29].

**Figure 7:**
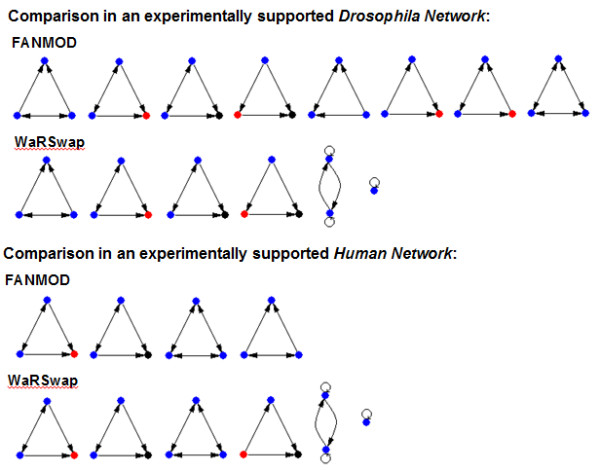
**Network motif outcomes from Fast Network Motif Detection (FANMOD) and WaRSwap compared for published *Drosophila *and human networks**.

We applied WaRSwap and FANMOD to each of these TF-miRNA-gene networks in exactly the same manner as for the *Arabidopsis *networks, and had a striking result from the motif comparison of the *Drosophila *network; the outcome was essentially identical to that of the *Arabidopsis *comparison. FANMOD predicted eight three-node motifs, whereas WaRSwap showed that only four of these motifs were truly over-represented: an miRNA-mediated FFL controlling a gene, and all three types of sustained-input switches (Figure 7). Additionally, WaRSwap also showed that the lock-on switch was a motif, as was TF auto-regulation.

The human network was a considerably smaller network, where there was more agreement between the two algorithms (Figure 7); there was agreement on three out of the four motifs, and two of these three were sustained-input switches. This comparison emphasizes that despite varying degrees of confidence in network construction, there is very strong support across multiple systems for the previously unrecognized sustained-input switch being an important over-represented circuit in eukaryotes.

## Discussion

### WaRSwap is necessary to distinguish motifs from artifacts

No practical solution has been presented previously to reliably handle motif finding for large directed networks with imbalanced node degree, which is a significant barrier for biologists aiming to understand regulatory networks. The FANMOD program implements an edge-switching algorithm to provide a fast-running solution for small networks and an option for multicolored input networks. However, the authors of FANMOD acknowledge that when a relatively small number of random graphs are sampled and/or the reported percentages of failed edge switches are high, the results are not reliable. These problems are alleviated as one approaches the limit of sampling an infinite number of random graphs. However, the long run times resulting from subgraph enumeration, even with three-node subgraphs and relatively small numbers of samples (around 1,000), are prohibitive, particularly when the method is not designed to run in parallel. As a result, analyses performed with good intentions that use a small fraction of even the minimum number of random graphs recommended for edge switching [30] are unlikely to yield meaningful results as the graph sizes grow larger. WaRSwap presents a practical solution for such analyses, providing a statistically justified algorithm for large TF-miRNA networks that can be run in parallel and with run times that do not compete with subgraph enumeration.

In this study, we tested and applied WaRSwap using large multi-layer graphs in *Arabidopsis *that involve both TFs and miRNAs, as well as non-TF protein-coding genes. Over a wide variety of parameterized networks, three motifs stood out: the sustained-input switch, the two-node bi-stable switch, and the miRNA-mediated FFL. By constructing these networks over a range of different binding-stringency requirements for TFs and miRNAs, network motif discovery can suggest a trend that is instrumental in hypothesis generation. For example, we found that the FFL in the *Arabidopsis *network was strongly over-represented using high-stringency settings, whereas the co-regulation of TFs, miRNAs, and genes by two upstream TFs in a sustained-input switch was an over-represented phenomenon primarily at intermediate TF binding-profile matches. It is reasonable to assume that the miRNA-mediated FFL may be predominant in situations where a gene pulse is desired in specific circumstances that are regulated by strong TF promoter signals and an excellent miRNA target-binding match. Our observation about FFLs is consistent with findings in human cells that use methods different from network motif discovery [8], as these studies often use conservation as a proxy for stringent binding requirements. Our results also illustrate the importance of how a putative biological network is constructed from data, as different underlying assumptions in a construction can yield striking differences in qualitative results. We note that our algorithm produced output that varied relatively smoothly with network parameterization (Figure 6), supporting the sufficiency of the sampling method and the reliability of the graph construction method. An algorithmic application comparison in *Drosophila *and human networks provides strong support for the validity of the findings of our main study in high-confidence *Arabidopsis *networks.

### WaRSwap highlights motifs associated with multicellular development

As in the human system, *Arabidopsis *is known to have both large TF source hubs as well as target hubs. One difference is that plant miRNAs are thought to target TFs preferentially (as opposed to a wide variety of gene classes). Given these similarities and differences, it is interesting that gene-expression studies have shown that the microRNA-mediated FFL is prevalent in animals [8], and seems to be a robust presence in the eukaryotic kingdom, including plants. In our investigation we found two types of network motifs that are associated with roles in development and fate specification, namely a sustained-input switch and a two-node bi-stable switch. This may be at least partly due to the character of the input set, as many of the TFs in our collection have clearly been of interest for their roles in such processes. Nonetheless, it is a useful hypothesis that these switches are over-represented in *Arabidopsis *networks because of their widespread function as crucial building blocks of developmental gene-regulation networks. The two-loop developmental switch is canonically noted in the systems biology literature as a developmental regulator [21], yet it has been absent from the literature on finding eukaryotic network motifs, because of the lack of suitable algorithms addressing large-scale networks containing TF self-loops. Additionally, we found many 'decorated motifs', that is, motifs that are significant when one or more nodes have self-loops. This finding might be partly due to the fact that self-loops themselves are over-represented in this particular system; however, it is well documented that smaller TF-only developmental networks tend to contain such motifs as well [21].

Regardless of self-loops, the sustained-input switch is a motif observed here for the first time as an over-represented network motif in a large eukaryotic system. This same result was obtained whether the sustained-input target was a TF, miRNA, or other coding gene, and always at a medium TF binding-threshold requirement, as mentioned above. This motif has clear implications in developmental network architectures because of its function when both of the 'input TFs' are serving as activators or repressors. It can be seen qualitatively that in the case where both are activators, upregulation of either input TF will result in sustained upregulation of the third gene (TF/miRNA/gene), leading to a lasting temporal effect on downstream targets. In the case where both are repressors, upregulation of either input TF will lead to downregulation of the other as well as of the third gene, allowing for a rapid shut-off signal in the presence of either input. In the case where one TF is an activator and the other a repressor, the dynamics of this circuit will depend on the specific physical parameters of the system, as there are a variety of possible time-dependent behaviors depending on signals to the input TFs, predominantly including time-delayed shut-off. All three of these behaviors are characteristic of developmental networks in higher eukaryotes [21], and the examples here suggest that roles may include cell-type differentiation and morphological patterning.

We used full enumeration of all network subgraphs (as opposed to a sampling method that yields approximate subgraph counts) to provide the highest possible confidence in our evaluation, This limited the practical motif size for testing and comparison to three-node motifs. With improved network subgraph sampling methods, it will be possible to examine four-node and five-node motifs with reasonable statistical confidence. *Arabidopsis *was chosen as a model organism for robust statistical comparison of network motif discovery algorithms, because of its low-noise TF and miRNA target prediction characteristics. We curated a very conservative subset of experimentally supported *Arabidopsis *TF binding-domain representations from those available in Plant TRANSFAC, a plant TF database [31]. With continually advancing large-scale binding-domain query technologies, such as protein binding microarrays [32], we anticipate a greatly increasing availability of plant PWMs in the near future. Finally, as miRNA target prediction methods continue to increase in sensitivity and specificity, along with experimental technologies for large-scale investigation of target binding, (for example, Degradome sequencing and photoactivatable-ribonucleoside-enhanced crosslinking and immunoprecipitation (PARCLIP) technologies), the WaRSwap algorithm provides a sound and practical method for exploring network motifs in large animal networks.

## Conclusion

Network motif finding is fundamentally a hypothesis-generation method rather than an endpoint to analysis, and as shown in bacteria and yeast networks [9,10], it can be a valuable computational tool for biological discovery on a systems-wide scale. However, if network motif discovery is to spur follow-up laboratory explorations in eukaryotic genomes, a practical and statistically sound method, which also respects the biology in these genomes, must be used. We have presented evidence that WaRSwap provides such a method, and shown that it can reveal patterns that would be difficult to ascertain by eye or with the use existing methods that are intended for use in much smaller genomes. This new method allowed us to identify new over-represented genetic sub-circuits that are specifically related to development. The repeated use of network motifs such as the sustained-input (or noise-damping) switch for both TFs and miRNAs supports the idea that these themes help to ensure correct patterning in multicellular organisms. Our study also provides fresh evidence supporting the hypothesis that one role of TF-miRNA interactions within the context of developmental networks is to canalize or buffer gene-expression changes in order to confer a robust spatial plan despite noisy environmental input signals [33]. Overall, the availability of reliable methods such as WaRSwap is expected to spur future studies as large, experimentally supported data sets become available in other genomes.

### Software availability

We have provided our implementation of the WaRSwap algorithm in R as publicly available open source code on the web [34] (see Additional file 4). At this same site, we have made available the modified command-line versions of FANMOD that we used in running the edge-switching randomizations in parallel, enumerating subgraphs, and performing randomization comparisons on small test networks. Both WaRSwap and FANMOD parallelizations were run on Sun Grid Engine; we have made the script sets available for others to use and adapt under the GNU Public License (GPL). Our TF binding-site scanning software is currently available on the web under the GPL as the 'Scanner Toolset' [27].

## Materials and methods

### WaRSwap algorithm

WaRSwap produces random graphs from an original graph by breaking the original into layers representing the five possible types of interactions: TF→TF, TF→miRNA, TF→gene, miRNA→TF, and miRNA→gene. Each layer is randomized using the Core Method (Figure 2), which is the key algorithm contained within the Complete Method described step-by-step below (Figure 3). For a given graph layer, the Core Problem is as follows.

Given a directed bipartite graph G with edges directed from source nodes S_1_, ..., S_i_, ..., S_n _to target nodes T_1_, ..., T_j_, ..., T_m_, we define the sample space Sp(G) as the collection of all graphs with the same in-degrees deg(S_1_), ..., deg(S_i_), ..., deg(S_n_) and out-degrees deg(T_1_), ..., deg(T_j_), ..., deg(T_m_) as G, but no double edges. Figure 2 provides a visual example.

WaRSwap addresses the Core Problem using the Core Method, outlined as follows.

0. Sort source out nodes so that S_1_, ..., S_i_, ..., S_n _are in descending order of out-degree magnitude.

For each source node S_i,_:

1) Compute sampling weights: for each target T_j_,

$$\text{sampling weight}={\text{cap(S}}_{\text{i}}{\text{)cap(T}}_{\text{j}}\text{)}\;\left[\text{1}–\text{deg}\left(\text{Si}\right)\text{deg}\left({\text{T}}_{\text{j}}\right)/\text{6m}\right].$$

2) If deg(S_i_) is less than or equal to the number unsaturated target nodes, match source node S_i _with deg(Si) target nodes T_j_, using draws that are weighted proportionally to sampling weights.

3) If deg(S_i_) is greater than the number of unsaturated target nodes, then perform the following.

3a) Place edges from S_i _to each target node T_j _that has available capacity.

3b) For each unplaced edge from S_i_, select an unsaturated target node of S_i_, and label it T_si_. Select a swap source S' having at least one target T_s' _that is NOT already targeted by S_i_. (Edge S' →T_s' _is drawn uniformly from among all possible such edges), and perform the swap by replacing edge S' → T_s' _with edge S' → T_si _and placing an edge from S_i _to T_s_'.

The WaRSwap Core Method proceeds to termination, resulting in a valid graph (the algorithm always terminates, it cannot encounter a loop state). The Pseudocode is provided (see Additional file 1), and R code is also provided (see section on 'Software Availability'). The WaRSwap Complete Method then uses the Core Method to address graphs with multiple layers using the following steps.

A) Break the original graph into layers representing each type of interaction

B) For each layer, permute target in-degrees (preserves target in-degree distribution), and apply the WaRSwap Core Method to the layer.

Algorithms that address the Core Problem face inherent tradeoffs between sampling uniformity of graph space Sp(G) and speed; these tradeoffs are exacerbated by non-uniform in-degree and out-degree distributions. The balance between speed and uniformity becomes extremely important in large graphs, G, where Sp(G) is enormous and it is therefore impossible to sample a large portion of the space. In such cases, a practical algorithm must be able to produce a near-uniform sample within an achievable amount of computing time. How does WaRSwap achieve fast near-uniform sampling performance, even in the case of extreme degree distributions (Figure 4) where the tendency of other algorithms is to grossly undersample graphs without any hub-hub edge connections [20]? Intuitively, the WaRSwap Core Method avoids undersampling of hub-hub connections by updating the source-target selection weights at each step (e.g. Step 1) so that the strong tendency to place edges between 'very heavy' sources and targets early in the process is offset by a factor proportional to the total desired weights of the vertices.

As shown in Figure 2, this offset factor is equal to 1 minus the product of total desired source-target vertex weights divided by a parameter of the algorithm, 6 × *m*, where *m *is the required number of edges in the graph (equal to the sum of total required source weights, and to the sum of total required target weights). It can be shown theoretically that for the case of near-regular undirected graphs, weight updating in this manner with a parameter of 4 × *m *will produce near-uniform sampling within an estimated number of attempts using the sampling method described in [14]; however, as graphs become highly irregular, this useful idea breaks down in two ways. First, it can be seen from the weight selection formula that with graphs containing highly uneven source and vertex degrees, if the parameter *p *× *m *is too small, the correction factor ultimately also becomes too small, and might even be negative (which would not make sense as a proportionality factor that should lie between 0 and 1). In the case of our method, we chose 6 as the smallest integer multiplier of the number of edges *m *such that in the case of all of our graphs under study, the proportionality constant is a valid factor between 0 and 1.

However, weighted edge selection algorithms of this general type can still get 'stuck' in the sense that they reach a state where no edge can validly be placed into the graph without creating a double edge. One solution is to discard the graph and start again; this is the case in the method of Bayati *et al*. [14]. However, the number of sampling attempts rapidly grows untenably large with increasingly irregular degree distributions. In the case of WaRSwap, sorting source out-degree from heaviest to lightest source and then distributing the source edge weight from the heavy sources first (Step 0, Steps 1-3) serves to reduce the chance of the algorithm getting 'stuck' in this way; but an impasse may still occur (Figure 2B). The reverse-swap step addresses this occurrence by finding a resolution to the currently 'stuck' state and continues forward to completion. Resolving swaps do slightly affect uniformity, but even in large graphs with very irregular in- and out-degree distributions, only a few resolving swaps are needed. For example, in the largest biological network in our analysis (FNR = 0.2, dG = 60 from Table 1), over the 2,500 random networks generated by WaRSwap, a maximum of 3 swaps and an average of 0.09 swaps per random network were required. The result of this combined approach in the Core Method was near-uniform sampling within each graph layer (Figure 3), both on near-regular graphs and on extreme graphs (Figure 4). The overall graph uniformity implications of the choice to preserve in-degree distribution while allowing self-loops are provided (see Additional file 1).

The current WaRSwap implementation is formulated using the R language with the igraph package [35] for the purpose of being clear, modular, and easy to extend as opposed to being coded for maximum runtime efficiency in a compiled language such as C. Thus, run times on a given computer processor are not comparable with FANMOD in a direct sense, yet the WaRSwap graph randomization time is well under the time required for subgraph enumeration, and therefore importantly is not a rate-limiting step. For example, in the biological networks associated with dG = 70 from Table 1, the mean WaRSwap randomization times were 4, 8, 17, and 30 seconds for FN = 0.8, 0.6, 0.4, and 0.2, respectively; mean subgraph enumeration times for these same networks were 1, 8, 43, and 109 minutes, respectively (means are taken over the 2,500 subgraphs computed on a variety of processors in our parallel computing cluster). FANMOD code was used for subgraph counting in all cases, using full subgraph enumeration as opposed to subgraph sampling estimates, in order to provide fully accurate subgraph counts for analysis and comparison. The FANMOD subgraph enumeration code was modified to output subgraph counts as opposed to frequencies, in order to eliminate the loss of numerical accuracy in the case of rarely observed subgraphs. SDs of the number of counts, z-scores, and *P*-values were then calculated directly from counts. Graph randomizations (and subsequent subgraph enumeration) were run in parallel with a Sun Grid Engine computing cluster, with each randomization using a pre-selected seed for the random-number generator (seeds drawn uniformly over the range of all possible seed choices) so as to be reproducible and unbiased by the system clock.

Enumeration of subgraphs from WaRSwap randomizations included not only three-node subgraphs but also two-loops and self-loops. Two-loop counting was performed using FANMOD with motif size set to 2 (none of the two-loops found significant), and the code was modified to perform self-loop counts. Decorated three-node motif analysis was performed by treating a self-loop decorated node as a fourth color. Thus, there is a difference between the analysis of subgraphs performed when the enumeration method ignores self-loops (for example, the unchanged version of FANMOD subgraph enumeration used for comparing WaRSwap output directly with FANMOD output), and when the TF nodes that have self-loops are considered as distinct from the TF nodes that do not have self-loops. For example, when self-loop decorated nodes are considered, the miRNA-mediated FFL targeting a TF is not a single subgraph but instead is associated with four unique subgraphs: one entirely undecorated, one in which both TFs have self-loops, and two in which a single TF has a self-loop. We have made both types of WaRSwap output (self-loops ignored, self-loops considered) available (see Additional file 5).

### Stability analysis

To determine the number of random graph samples that was sufficient to provide a stable WaRSwap *P*-value over all three-node subgraphs for the networks under examination, we performed the following evaluation. For each of the four TAIR10 (The Arabidopsis Information Resource, version 10) *Arabidopsis *networks constructed with a miRNA target stringency of dG = 60, 10,000 random networks were generated using WaRSwap by seeding a random-number generator according to 10,000 uniform draws over the interval of all possible seeds. From this selection, the *P*-values were determined for all three-node motifs for subgraphs 1 to 500, 1 to 1,000, 1 to 1,500, ..., 1 to 10,000. For each group of (500, 1,000, 1,500, ..., n ,..., 10,000) random networks, for each three-node subgraph, the absolute value of the difference between the *P*-value computed with n random networks and the *P*-value computed with 10,000 random networks was recorded. These *P*-value differences were plotted for each subset of n networks as a box plot (see Additional file 1, Figure S4). The *P*-value difference distributions narrowed rapidly at first and then leveled off. Thus at 2,500 random networks, this distribution of *P*-value differences over all three-node subgraphs fell entirely below 0.02 for all four networks examined. That is, the maximum difference between the *P*-value for any three-node subgraph at 2,500 random networks generated and 10,000 random networks generated is 0.02. Mean differences in *P*-value at this level are negligible. We thus considered 2,500 random networks to be more than sufficient for stable outcomes over the networks in our study.

### Comparison with the FANMOD implementation of edge switching

The FANMOD implementation of the edge-switching algorithm was modified to provide the ability to execute one network randomization at a time, thus allowing many subgraph randomizations to run in parallel using Sun Grid Engine. Just as with WaRSwap, the random-number generator used in FANMOD was seeded with a uniformly drawn selection of seeds from the full range of possible seed values to avoid any bias due to the system clock in parallel runs. Enumeration was performed in all cases to provide a full count of all three-node subgraphs for each randomization. Subgraph counts, SDs, and *P*-values were then calculated from these counts in exactly the same way as for WaRSwap. We compared WaRSwap and FANMOD over the grid of networks displayed in Table 1 below, parameterized according to TF binding-site stringency and miRNA target-binding stringency (discussed in detail in the 'Datasets' section). Two problems were readily apparent with the FANMOD output: the large percentage of failed edge switches (Figure 1B), and the relatively small SDs of subgraph counts on average. Allowing FANMOD to make an increasingly large number of edge-switch attempts did not result in changed percentages of failed swaps per run, but only in increased run times.

We explored the reasons for these observed problems, and discovered that a theoretical basis for these difficulties had already been noted in previous studies (see Introduction). For a simple intuitive explanation, notice that edge switching begins from a particular given network and moves away from it in a series of edge switches. This strategy is in contrast to strategies in algorithms such as WaRSwap, which 'seek' into the space of all possible network randomizations with the given in-degree and out-degree requirements, and produce a valid instance. In 'moving away' in a series of edge-switching steps, subgraph topologies are created and broken, and it may take a long time to reach certain valid graphs.

Suppose that a limited number of possible random graphs is strongly favored by such searches, because the total space cannot be thoroughly explored in a reasonable number of swapping steps. Even over many randomization attempts, this may produce a distribution of certain subgraph counts which is narrow in range (small SD) and unrepresentative of all possible outcomes. When this happens, it is easy to observe a spurious *P*-value because the subgraph count value from the original network appears to be highly unusual compared with this narrow range of values from the 'randomized' graph collection.

## Datasets

### TFs, TF binding sites, and TF target regions

Transcription factors and their binding profiles were derived from Plant TRANSFAC [31], a literature-curated database containing 115 PWM representations of plant TF binding domains. We first divided this collection into a conservative *Arabidopsis*-only set, in which one or more literature-supported binding observations from *Arabidopsis *was used in the TRANSFAC construction of the PWM, and a 'Comprehensive' set. To be included in the Comprehensive set, a PWM was required to be associated with at least one TF having an identifiable *Arabidopsis *ortholog within the Gramene Database [36] or PlantTFDB (Plant Transcription Factor Database) [37]. Finally, PWMs were filtered to remove binding profiles with low-information content given the AT-rich promoter background of *Arabidopsis *(that is, PWMs having too much overlap with background sequence content were removed). Specifically, the distribution of log-likelihood scores of sequences drawn from the background (all TAIR protein-coding gene upstream sequences of length 1,000) was computed, along with the distribution of log-likelihood scores from the PWM itself (see Additional file 1; see Additional file 6; see Additional file 7). PWMs were eliminated from the set if the distribution overlap was such that the 10% left tail of the PWM score distribution corresponded to a false-positive rate (FPR) of 0.005 or greater. Receiver operating characteristic (ROC) curves for all PWMs are provided (see Additional file 8; see Additional file 9).

The filtered *Arabidopsis*-only set contained a total of 41 PWMs corresponding to 44 TAIR-identifiable TFs, whereas the filtered Comprehensive set contained 81 PWMs corresponding to 138 TAIR-identifiable TFs. Given the weaker link between the binding domains in the Comprehensive set and the specific *Arabidopsis *TFs, we chose to focus our primary TAIR10 analysis and results on networks constructed from the *Arabidopsis*-only set. However, we have made available all constructed networks and motif outcomes for the Comprehensive PWMs in our TAIR9 dataset described below. In the network constructions described in our Results section, the TAIR10 *Arabidopsis*-only PWM set was scanned over the upstream regions of TFs, miRNAs, and non-TF protein-coding genes using log-likelihood scanning [38,39] and a first-order Markov background model (which accounts for dinucleotide sequence content). PWM-specific thresholds were computed for four separate binding-match stringencies, namely, FN rates of 20%, 40%, 60%, and 80% (lowest to highest stringency). FN rates correspond to the percentage of potential binding sites that could have been drawn from the PWM scoring distribution, but that did not pass the threshold as a sufficiently strong match to be considered a binding site. We used FN rates because we required that only PWMs with high information content could be used. The FP rates are therefore very close to zero at these values, yet we still wish to narrow the accepted quality of binding matches (see Additional file 1 for a diagram, along with average hit rates per kilobase of sequence and ROC curves for all PWMs).

TF target regions were taken as the upstream regions of TFs, miRNAs, and non-TF protein-coding genes as follows. For each entity, an approximate TSS was determined (using either the TAIR10 annotation or, in the case of miRNAs, additional experimental evidence, as described below). A region of up to 3,000 nt upstream of this TSS was then extracted as the target region; if an upstream gene on either strand intervened in this 3,000 nt region, then the region was shortened to include only the sequence between the TSS and the neighboring gene. In general, *Arabidopsis *has short intergenic regions (around 2,500 nt on average when considering genes on both strands), making the determination of putative binding sites substantially easier than in many other organisms, because of the reduced search space. Additionally, it has been shown that 3,000 nt of upstream sequence is enough to recapitulate the expression patterns of approximately 80% of *Arabidopsis *genes in a large set of transcriptional fusion experiments [40].

### miRNAs and miRNA targets

We performed our TAIR10 analysis using a curated set consisting of of miRBase (version 18.0) miRNAs along with a small additional set of the latest published *Arabidopsis *miRNAs [41]. Intronic miRNAs were removed from the set because it remains uncertain whether these would necessarily be transcribed along with their host genes in all cases. The miRNA ath-miR169g* was corrected to its proper mature sequence, and ath-miR406 was removed from the set [41]. The remaining miRNA precursors and their reported mature sequences were used for determining the miRNA promoter regions and target genes as follows. In cases where one or more experimentally supported TSS was reported for a miRNA [42], the most upstream part of these sites was used as the TSS. In the remaining cases, the upstream end of the precursor was taken as the approximate TSS.

Several sets of miRNA target sites were determined as follows. Predicted binding sites were determined using the WMD3 Target Search software package [43], which was selected for this project because it is the only *Arabidopsis *miRNA target prediction tool implemented based on rules that were determined directly from both *in vivo *and *in vitro *binding observations [44,45]. The WMD3 tool was locally installed, and targets were determined for three different binding energy requirements: dG = 60, dG = 70, and dG = 80, with 80 being the most stringent requirement (other WMD3 settings were left at the defaults except for the 'Show Only One Isoform' option, which was set to false so that all target transcripts could be identified). The sets were nested, that is, the most liberal binding requirement of dG = 60 contained all targets that are predicted to bind under the requirement that dG is at least 60. These sets were then filtered to remove any target marked by the program as a 'possible candidate', that is, a target that does not strictly satisfy all binding rules observed for endogenous miRNAs.

It should be noted that the networks generated using these data sets in our study are not independent; in fact, they depend on each other in a complex way because of the TF binding site and miRNA target stringency cutoffs (for example, miRNA targets with a dG requirement of at least 80 are a subset of those targets with a dG requirement of at least 60). It is not clear in this situation how to apply multiple hypothesis testing, and we did not do so, as the purpose was to analyze the output of the WaRSwap method relative to alternatives. We invite future analyses using the WaRSwap method with other datasets.

### Non-TF protein-coding gene set

We curated the complete publically available TAIR10 gene set TAIR10_GFF3_genes.gff by restricting it only to genes identified in the gene descriptor field as a 'protein_coding_gene', for a remaining total of 27,416 genes (see Additional file 10). All genes considered as TFs in our Plant TRANSFAC TF sets detailed above were then labeled as TFs rather than genes in our use of this set for TFBS scanning and motif finding. We recognize that this set would still have contained a small proportion of genes that are in fact TFs but are not labeled as such, because there is no currently available corresponding PWM in Plant TRANSFAC. TSSs were taken as the TAIR10 annotated sites for all genes in this set.

### TAIR10 versus TAIR9 assembly datasets

For the analysis that is the main focus of our results and discussion above, we used the TAIR10 *Arabidopsis *genome annotation, which includes miRNAs corresponding to miRBase version 18.0 along with the most recently discovered *Arabidopsis *miRNAs [41] discussed in detail above (see section on 'miRNAs and miRNA targets'). We have also provided network motif outcomes from the TAIR9 annotation (see Additional file 1), which includes only miRNAs corresponding to an older miRBase (version 14.0). We refer to these studies in the text as the TAIR10 and TAIR9 sets, respectively, and the network motif outcomes from each are nearly identical. The TAIR10 and TAIR9 *Arabidopsis *annotations are based on precisely the same genome assembly; only the annotation and coordinates have been updated in TAIR10.

We used the TAIR9 set for several preliminary studies prior to the release of TAIR10, including for the analysis of network motif outcomes when different upstream region lengths of TFs and miRNAs were considered for TF targeting. Network motifs were compared for promoter region sets of 3,000 nt, 2,000 nt, and 1,000 nt for both WaRSwap and FANMOD, and in all cases, the WaRSwap results for three-node three-edge motifs were a nested subset of the FANMOD results (see Additional file 1, Figure S2). WaRSwap gave similar outcomes across all three types of regions, whereas FANMOD reported an increasing number of motifs as the promoter region size actually decreased. We believe this may be due to the fact that edge switching is more susceptible to dataset noise, as discussed above in the Results section.

### *Drosophila *and human networks

We used the TF-miRNA-gene network provided in the data file DataS9_pRN_network.txt of the modENCODE *Drosophila *study [28]. That study applied FANMOD to the network to report eight motifs, and our application of FANMOD also produced eight motifs. However, only seven of the eight motifs in both studies agree, This variation in reporting is probably due to our generation of 2,500 background networks versus the 100 background networks reported to have been used by the modENCODE study [28]. We used the human TF-miRNA-gene network in the combination of data files tfmir.txt, mirgene.txt, and proxraw.txt provided by Gerstein *et al *[29]. The publication [29] applied FANMOD to a network that was not explicitly provided, and did not provide a direct report of miRNA-TF-gene motifs, thus we were unable to confirm that the network we used was identical to that of the study. However, although the study reported the number of miRNA-mediated FFLs in the network, it did not report this circuit to be statistically over-represented, and we also did not find that either FANMOD or WaRSwap reported any miRNA-mediated FFLs as motifs.

## Abbreviations

DG: deltaG; DIA-MCIS: Diaconis Monte Carlo Importance Sampling; FANMOD: Fast Network Motif Detection; FFL: Feed-forward loop, transcription factor APETALA1, AP1; transcription factor LEAFY, LFY; transcription factor PI, PI; FNR: False-negative rate; FPR: false-postive rate; GO: Gene Ontology; miRNA: MicroRNA; PWM: positional weight matrix; ROC: receiver operating characteristic; TF: Transcription factor; WaRSwap: Weighted-and-Reverse-Swap.

## Competing interests

The authors declare that they have no competing interests.

## Authors' contributions

MM and UO designed the study. MM carried out the experiments, data analysis, algorithm design and implementation. SM and UO contributed to algorithm design. MM wrote the paper, SM and UO contributed to editing of the paper, all authors read and approved the final version of the paper.

## Supplementary Material

Additional file 1 **Main Supplement**. Main supplement, contains table of contents for this and all additional files, and detailed description of all additional files. Contains supplementary figures and text. Click here for file

Additional file 2 **Tissue Co-expressed Motif Instances**. Specific motif instances having the property that entity involved in the circuit has experimental support for gene expression within the same Arabidopsis root cell type. Click here for file

Additional file 3 **GoStat Analysis Output**. Complete output from GoStat analysis described in the main text. Click here for file

Additional file 4 **WaRSwap Code**. Zipped folder containing README, R code, and sample Bash calling script. Click here for file

Additional file 5 **HTML Browsable Motif Output**. Zipped folder containing all WaRSwap and FANMOD motif output, viewable in a web browser. Click here for file

Additional file 6 **PWM Log-likelihood Score Distributions, Arabidopsis Only Set**. Zipped pdf file containing plots of foreground and background log-likelihood scoring distributions for each PWM in the Arabidopsis Only set. Click here for file

Additional file 7 **PWM Log-likelihood Score Distributions, Comprehensive Set**. Zipped pdf file containing plots of foreground and background log-likelihood scoring distributions for each PWM in the Comprehensive set. Click here for file

Additional file 8 **PWM ROC Curves, Arabidopsis Only Set**. Zipped pdf file containing ROC curve plots for each PWM in the Arabidopsis Only set. Click here for file

Additional file 9 **PWM ROC Curves, Comprehensive Set**. Zipped pdf file containing ROC curve plots for each PWM in the Comprehensive set. Click here for file

Additional file 10 **TAIR10 Protein Coding Gene Set**. Zipped gff file containing the curated TAIR10 gene set used in our analysis. The publically available file TAIR10_GFF3_genes.gff was filtered by restricting it only to genes identified in the gene descriptor field as a "protein_coding_gene", for a remaining total of 27,416 genes. Click here for file
