# Supplementary material for: Sustained-input switches for transcription factors and microRNAs are central building blocks of eukaryotic gene circuits
Source: Genome Biol. 2013 Aug 23;14(8):R85. doi: 10.1186/gb-2013-14-8-r85 (PMC4054853; doi:10.1186/gb-2013-14-8-r85)

Supplementary Information

for

Sustained-input switches for transcription factors and microRNAs are central building blocks of eukaryotic gene circuits

Molly Megraw[1,7,8*], Sayan Mukherjee[2,3,4] & Uwe Ohler[1,3,5,6].

[1]Institute for Genome Sciences & Policy, Department of [2]Statistical Science, [3]Computer Science, [4]Mathematics, and [5]Biostatistics & Bioinformatics, Duke University, Durham, NC, USA.

[6]Berlin Institute for Medical Systems Biology, Max Delbrück Center for Molecular Medicine, Berlin, Germany.

[7]Center for Genome Research & Biocomputing, and [8]Department of Botany & Plant Pathology, Oregon State University, Corvallis, OR, USA.

[*]Corresponding author
Email: megrawm@science.oregonstate.edu

Table of Contents

Figure S1 ……………………………………………………………………...………. 2
Figure S2 ……………………………………………………………………...….. 3-5
Figure S3 ……………………………………………………………………..……………. 6
Figure S4 ……………………………………………………………………...…………. 7
Additional File 2: Supplementary Dataset ..….…………..……………………………………... 8
Additional File 3: GOStat Analysis Output…....…………..……….…………………………... 8
Pseudocode for the WaRSwap core algorithm ……………………………...……….. 9-10
Graph uniformity implications of the choice to preserve in-degree distribution ……………… 11
Additional File 4: R Code for WaRSwap, .zip description ………..………………………..….12
Additional File 5: HTML-browsable motif output for WaRSwap and FANMOD…...…………12
Additional Files 6,7: PWM Log-likelihood Score Distributions ………….……….…..……13-15
Additional Files 8,9: PWM ROC Curves ……………………...……….………...…………15
Average TF Binding Site hit rates per KB of sequence ………………………………………16
Additional File 10: TAIR10 Protein Coding Gene Set……………..…………………………....16
Degree variation and sampling uniformity…………………………………………………...17-20

Figure S1.

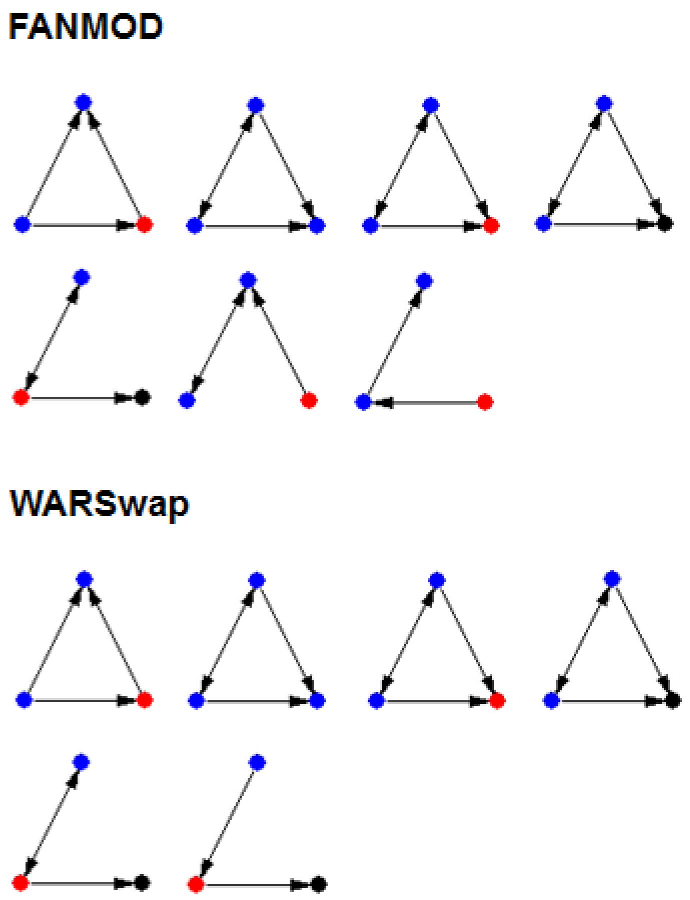

Supp. Figure S1: For both FANMOD and WaRSwap methods, we considered a 3-node subgraph to be a motif if it was over-represented in one or more of the biological networks with the simultaneous requirements of a p-value less than 0.01, a z-score greater than 2.0, and a standard deviation (reported in terms of number of observations) greater than 1. All resulting motifs are shown here. WaRSwap motifs are a near-nested subset of FANMOD motifs. TAIR10 dataset and Arabidopsis-specific PWMs were used.

Figure S2A.

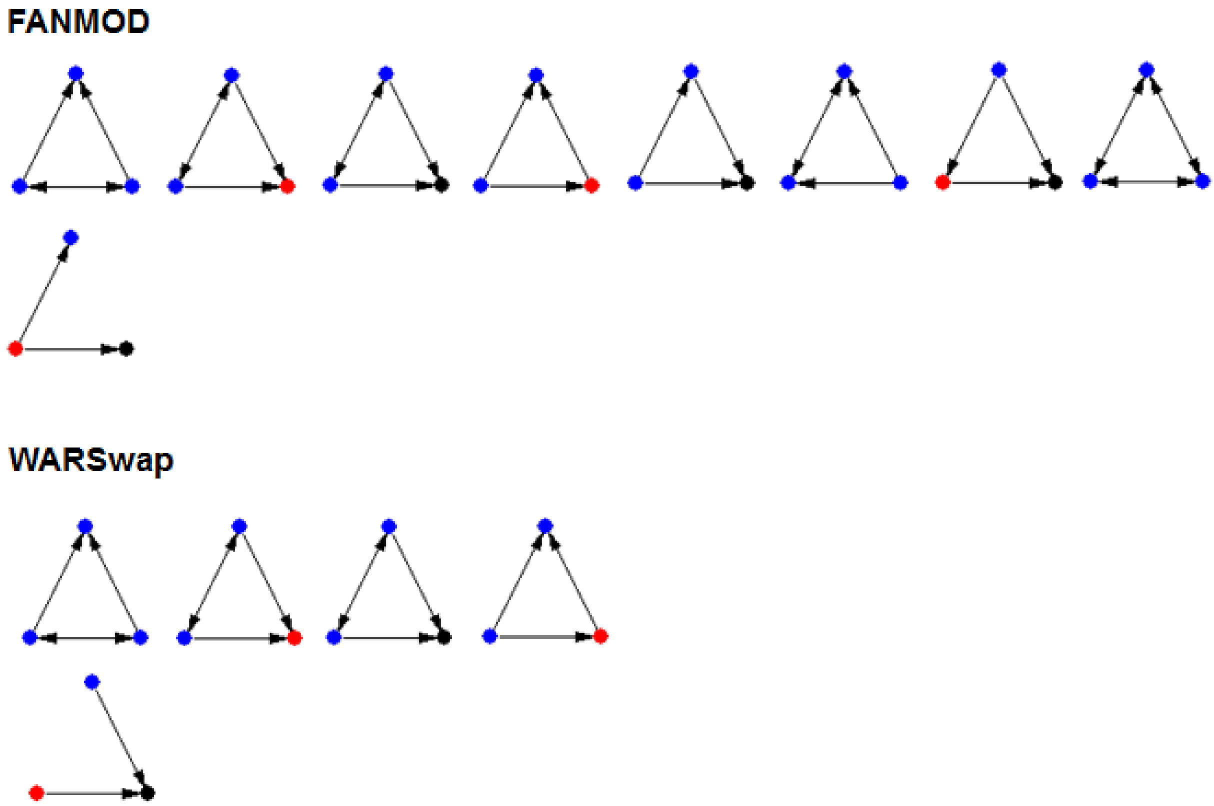

Supp. Figure S2A: Results analogous to Supp. Figure S1, here the TAIR9 dataset was used (Arabidopsis specific PWMs and 3000nt promoter regions were used just as in Supp. Figure S2).

Figure S2B.

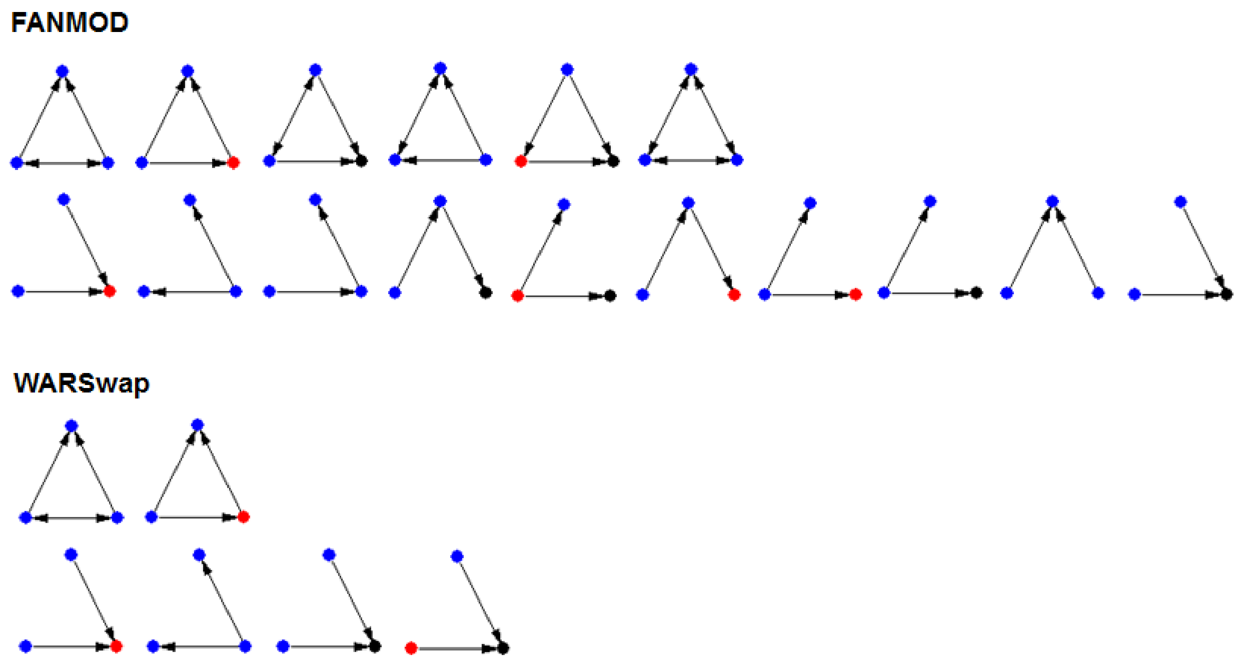

Supp. Figure S2B: Results analogous to Supp. Figure S1, here the TAIR9 dataset was used (along with Arabidopsis specific PWMs) but with 2000nt promoter regions.

Figure S2C.

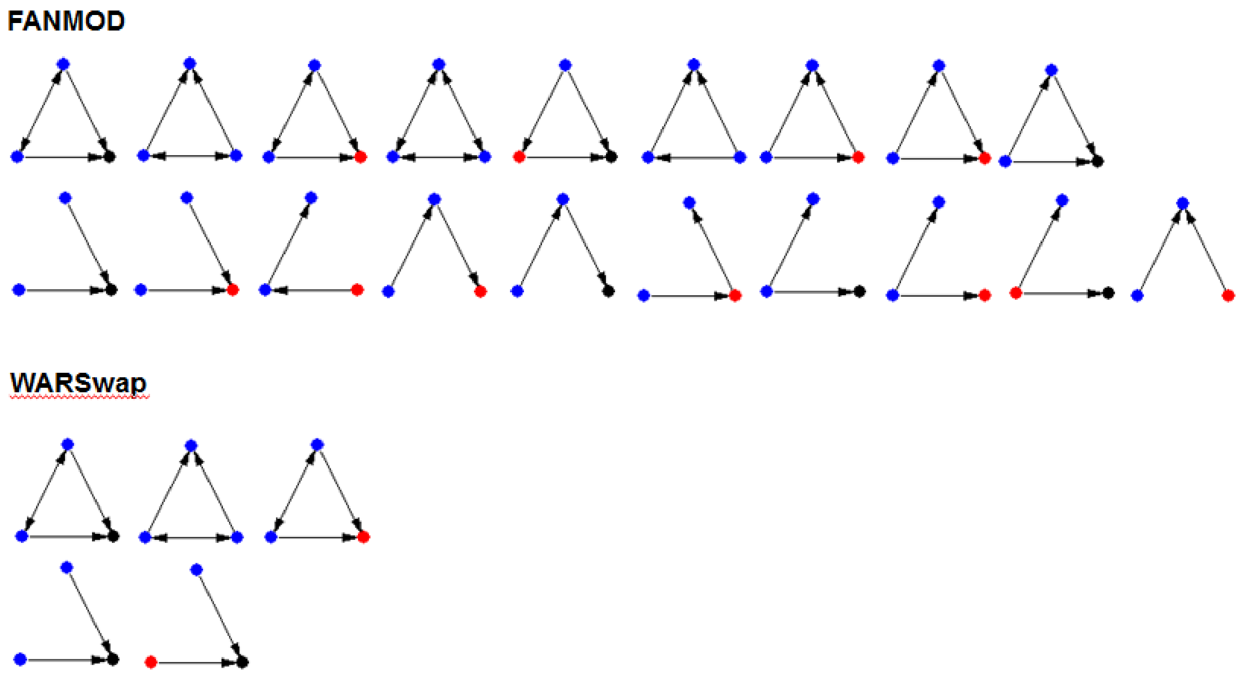

Supp. Figure S2C: Results analogous to Supp. Figure S1, here the TAIR9 dataset was used (along with Arabidopsis specific PWMs) but with 1000nt promoter regions.

Figure S3.

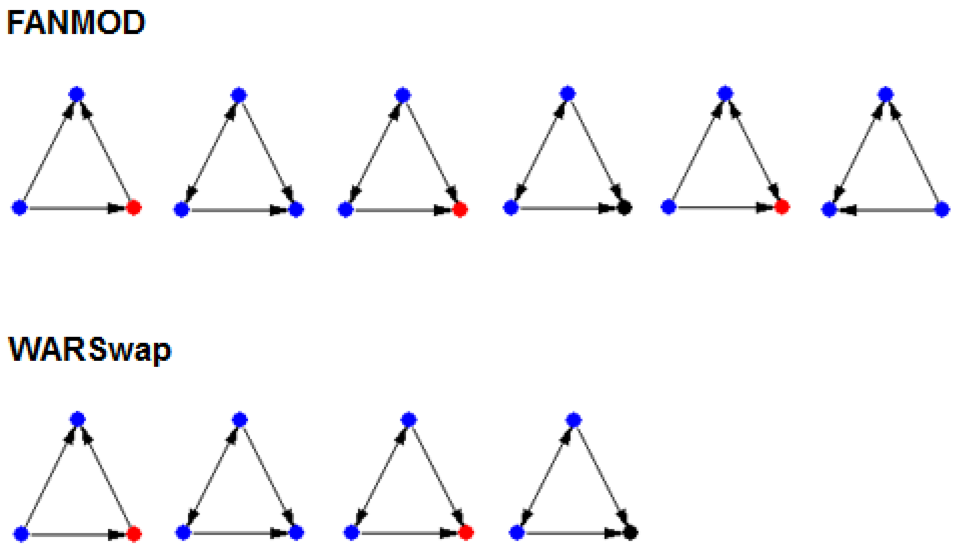

Supp. Figure S3: For both FANMOD and WaRSwap methods, we considered a 3-node subgraph to be a motif if it was over-represented in one or more of the biological networks with the simultaneous requirements of a p-value less than 0.01, a z-score greater than 2.0, and a standard deviation (reported in terms of number of observations) greater than 1. Resulting motifs where all 3 nodes have interactions (edges) between them are shown here. WaRSwap motifs are a proper subset of FANMOD motifs. TAIR10 dataset and Arabidopsis-specific PWMs were used.

Figure S4.

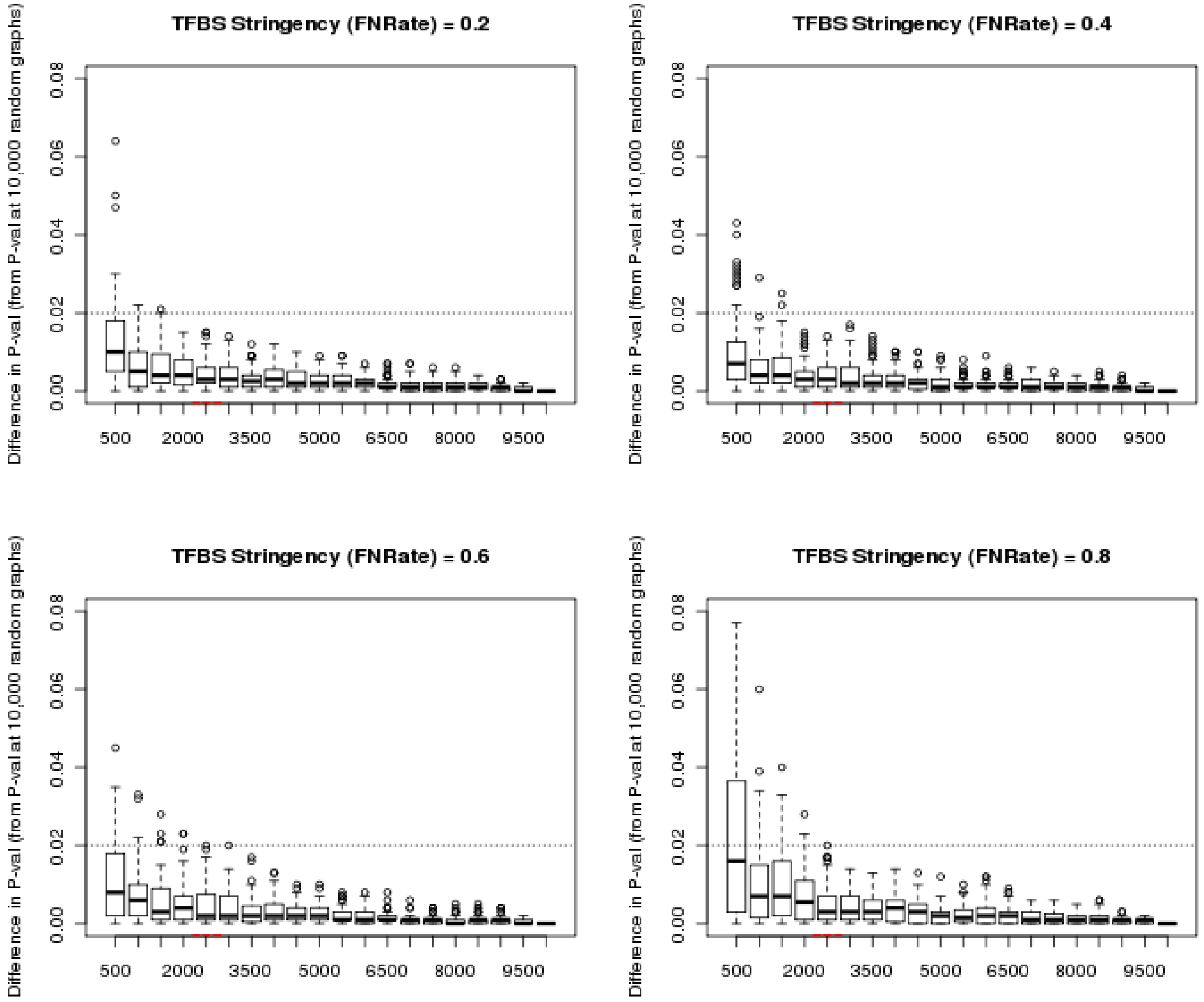

Supp. Figure S4: The boxplots below display the absolute differences in p-value when N=500,1000, …, 9,500 random networks are generated by WaRSwap vs 10,000 random networks. The dotted line in each plot represents a p-value difference of 0.02. 2500 random networks per background distribution was considered more than sufficient as all 3-node subgraph p-value differences fall below 0.02, therefore WaRSwap and FANMOD motif searches were compared using this number.

Additional File 2: Supplementary Dataset Description

Using the TAIR10 gene set and the Arabidopsis-only TF set (results from the main study), we identified thirteen instances of miRNA-mediated FFLs, eight 2-node bistable switches, and several hundred sustained-input switches targeting TFs and miRNAs such that every entity involved in the circuit has experimental support for gene expression within the same Arabidopsis root cell type. We provide an Excel Spreadsheet with these circuits, each reports the circuits for the highest level of TF and miRNA binding stringency for which the motif was found significant according to our study (these stringencies are detailed for each motif type in the spreadsheet pages). Evidence for cell-type specific TF expression was taken from the supplementary data of the "Arabidopsis Root Map" (Brady SM, Orlando DA, Lee JY, Wang JY, Koch J, Dinneny JR, Mace D, Ohler U, Benfey PN. 2007. A high-resolution root spatiotemporal map reveals dominant expression patterns. Science 318: 801-806), .CEL files were normalized using the RMA method in the Bioconductor package, a TF was conservatively considered expressed in a cell type if normalized expression value exceeded 5.0. Evidence for miRNA expression within the same Arabidopsis cell type was taken from the supplementary data of a recent Arabidopsis root cell-type specific RNA-Seq study (Breakfield N, Corcoran D, Petricka J, Shen J, Sae-Seaw J, Rubio-Somoza I, Weigel D, Ohler U, Benfey P. 2012. High-resolution experimental and computational profiling of tissue-specific known and novel miRNAs in Arabidopsis. Genome Research 22: 163-176). A miRNA was considered to be expressed if it was reported as expressed above the study-accepted RNA-Seq read baselines for each cell type. In separate sheets for each circuit type, the spreadsheet provides TAIR10 gene IDs and miRBase mature miRNA names for all entities in each circuit, along with the Arabdopsis root cell types where these entities were co-expressed.

Additional File 3: GOStat Analysis Output

We provide the complete output of our GOStat analysis, which includes the TAIR10 gene list considered, AdditionalFile3_GOStat_output.zip. This analysis is described in the "Developmental circuits are building blocks of the Arabidopsis gene network" section of the main text results.

Pseudocode for the WaRSwap core method

Given source nodes S_1, …, S_i, …, S_n and target nodes T_1, …, T_j, …, T_m (as shown in the Figure 1 example):

Sort source out nodes so that S_1, …, S_i, …, S_n are in descending order of out-degree magnitude.

For each source S_i,

Compute Sampling Weights:

For each target T_j,

Sampling Weight = cap(S_i) cap(T_j) [1 – deg(Si)deg(T_j)/6m]

If deg(S_i) ≤ # unsaturated target nodes,

Match source node Si with deg(S_i) target nodes T_j using draws weighted proportionally to Sampling Weights.

If deg(S_i) > # unsaturated target nodes,

Perform swaps:

Place edges from S_i to each target node T_j with available capacity

Update capacity of target nodes

Update sampling weights (target capacities have changed)

For each unplaced edge from S_i,

Draw a permutation of unsaturated target nodes of S_i (weighted by available capacity), T_{si_1}, …, T_{si_k}, …., T_{si_p},

Traverse the permutation until a node with an allowable swap is identified as follows:

For each unsaturated target node T_{si_k} in the permutation,

Identify source nodes S'_1, …, S'_q, …, S'_r that currently have assigned targets that are not targets of S_i, AND that do not target T_{si_k}.

Draw one of these source nodes S′ (weighted according to source out-degrees)

Identify targets of the selected swap source S′ that are NOT already targeted by the current source S_i; select one at random (label it $T_{s'}$).

If allowable swap was identified,

> Perform swap by replacing edge $S' \rightarrow T_{s'}$ with edge $S' \rightarrow T_{si\_k}$ and placing an edge from S_i to $T_{s'}$.
>
> Update sampling weights.
>
> Exit permutation traverse.

Graph uniformity implications of the choice to preserve in-degree distribution while allowing self-loops

The TF-TF layer of a graph is allowed to contain self-loops. As a result, target in-degree permutations produce different graph spaces—to each target in-degree permutation is associated a graph space of a certain (generally different) size. In order to preserve sampling uniformity of the entire graph composed of all layers, one would need to weight samples from each target in-degree permutation according to the (roughly estimated) size of the associated graph space. However, this would not preserve the distribution of target in-degrees within each layer; thus, it is generally not possible to preserve both perfect uniformity of sampling across the entire multi-layer graph and preserve target in-degree distribution within each layer if one or more layers contains self-loops. WaRSwap makes the specific choice to preserve target in-degree distribution within each layer, in keeping with the biological principle that both TF binding elements within gene promoters and miRNA target elements may arise and be destroyed in the natural process of genomic evolution (though it is reasonable to assume that the overall number of targets of a particular miRNA or TF remains the same). This is in contrast to methods such as edge-switching that preserve exact target in-degrees, modeling specific TF and miRNA target sites as static.

Additional File 4: R Code for WaRSwap

An implementation of the complete WaRSwap algorithm along with supporting scripts is provided as a zipped archive, AdditionalFile4_WaRSwap_code.zip.

Additional File 5: HTML-browsable motif output for WaRSwap and FANMOD

We make all motif output (including heatmaps, p-values, z-scores, and count standard deviations) available over the full grid of TF binding site and miRNA target datasets. We provide a web-browsable HTML file along with corresponding files and images for each dataset in our study as folders within the zipped archive AdditionalFile5_HTML_browsable_motif_output.zip, including:

WaRSwap output, TAIR10 dataset, Arabidopsis TFs, self-loops not considered: WaRSwap_ath_tair10

FANMOD output, TAIR10 dataset, Arabidopsis TFs, self-loops not considered: FANMOD_ath_tair10

WaRSwap output, TAIR10 dataset, Arabidopsis TFs, self-loops considered: WaRSwap_ath_tair10_withSelfLoops

WaRSwap output, TAIR10 dataset, Arabidopsis TFs, 2-loops with self-loops considered: WaRSwap_ath_TwoLoops_withSelfLoops

WaRSwap output, TAIR9 dataset, Arabidopsis TFs, self-loops not considered: WaRSwap_ath_tair9

FANMOD output, TAIR9 dataset, Arabidopsis TFs, self-loops not considered: FANMOD_ath_tair9

WaRSwap output, TAIR9 dataset, Comprehensive-Set TFs, self-loops not considered: WaRSwap_all_tair9

Additional Files 6,7: PWM Log-likelihood Score Distribution Plots

For both the Arabidopsis-only and Comprehensive PWM sets, we computed the distribution of log-likelihood scores of sequences drawn from the background (all TAIR protein coding gene upstream sequences of length 1000) , along with the distribution of log-likelihood scores from the PWM itself. We provide plots of these score distributions in zipped file archives AdditionalFile6_ath_pwms_hists and AdditionalFile7_all_pwms_hists. Each plot shows the PWM score distribution in blue, and the background score distribution in black. Consider for example the plot below:

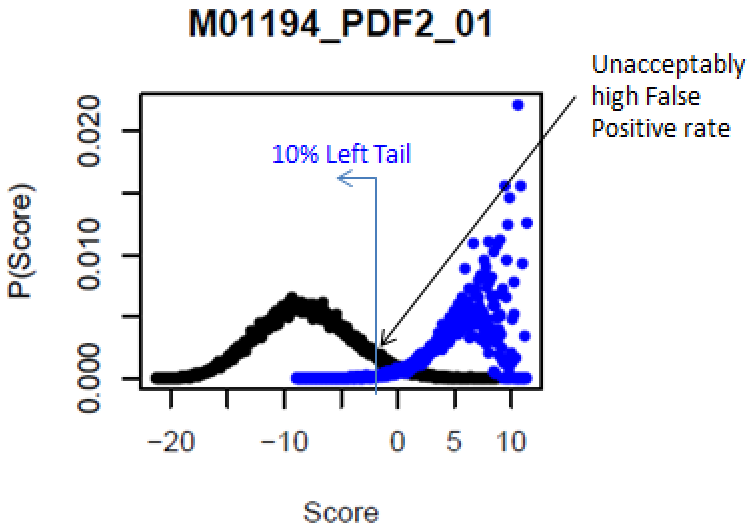

We can see that some PWMs do a poor job of distinguishing background sequences from those that match the PWM's binding profile—their score distributions overlap too heavily. We eliminated a PWM from our set if the distribution overlap was such that the 10% left tail of the PWM score distribution corresponded to a False Positive Rate (FP rate) of 0.005 or greater.

Now consider the following situation, where the two distributions are extremely well-separated:

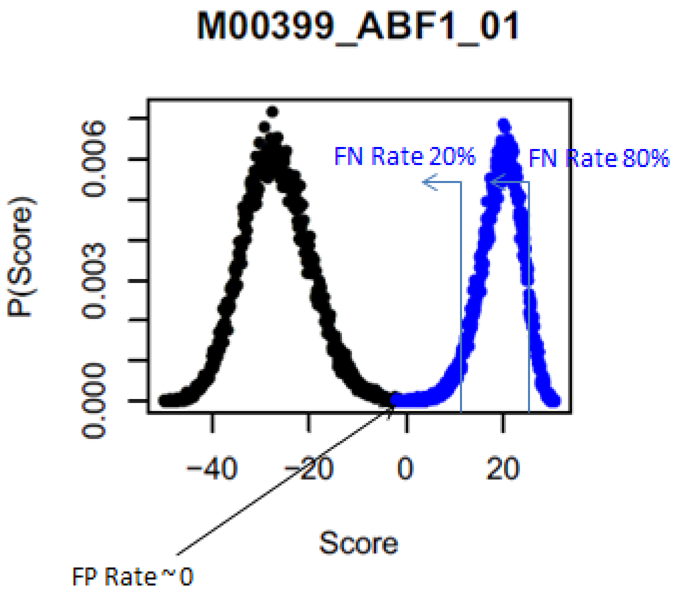

We can see that even for scores where the False Positive rate is essentially zero, many high-scoring sequence matches to the PWM are still possible. Thus, after eliminating PWMs where there was too much overlap, we still observed very high TF binding site hit rates in our promoter sets for most PWMs; we therefore used False Negative Rates of 20%, 40%, 60%, and 80% to insist on very stringent TF binding matches. Most PWMs in our set have score distributions like the one below:

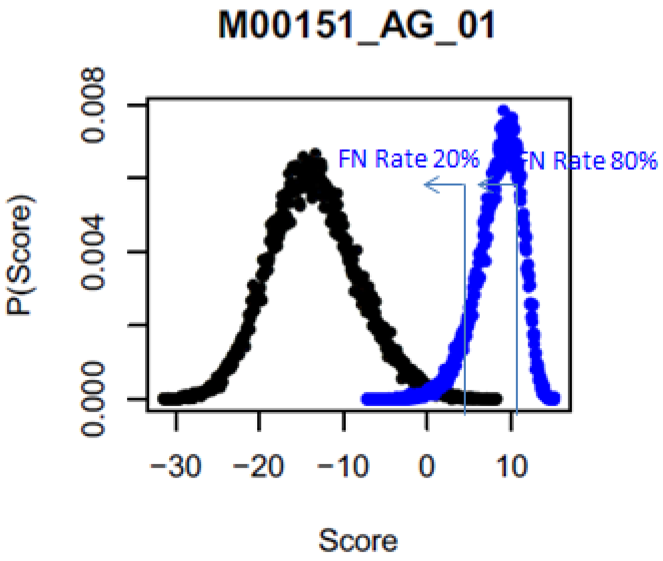

There is a small overlap between the PWM and background distributions, but FN Rates still do the best job of capturing progressively more stringent binding requirements at a low FP Rate.

Additional Files 8,9: PWM ROC Curve Plots

For both the Arabidopsis-only and Comprehensive PWM sets, we also computed ROC curves (plots of True Positive Rate vs False Positive Rate). We provide plots of these score distributions in zipped file archives AdditionalFile8_ath_pwms_roc_curves and AdditionalFile9_all_pwms_roc_curves. As a result of the PWM filtering procedure described immediately above, the PWMs used in our study all have an area under the ROC curve of "auROC" of nearly 1. This is clearly visible from the ROC curves, for example here is the ROC curve for the "typical" PWM M00151_AG_01 whose score distribution is shown in the section above:

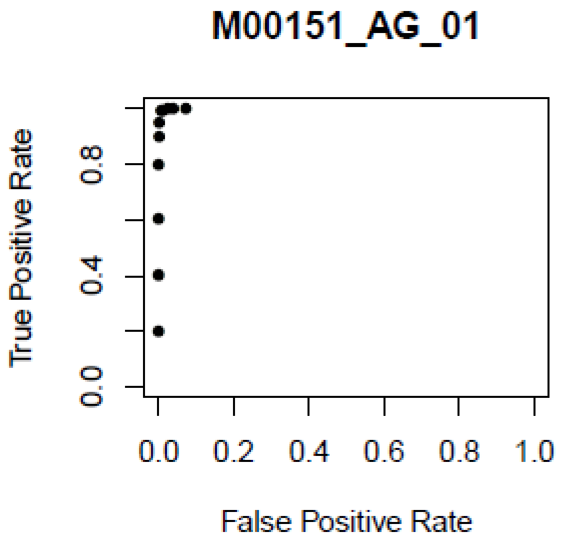

Average TF Binding Site hit rates per KB of sequence

We selected the FN Rates for our TF binding site sets of 20%, 40%, 60%, and 80% to produce increasingly strict requirements on TF binding stringency. These sets correspond to relatively low hit rates in our promoter sets. The average hit rates per TF per kilobase (KB) of promoter sequence from our sets was approximately 0.6, 0.2, 0.06, and 0.02 hits per KB for FN rates of 20%, 40%, 60%, and 80% respectively. So for example, at FN rate 40% (hit rate 0.2), on average we observed one TF binding site hit for approximately every 5 KB of sequence in our promoter sets.

Additional File 10: TAIR10 Protein Coding Gene Set

We curated the publically available TAIR10 gene set TAIR10_GFF3_genes.gff by restricting it only to genes identified in the gene descriptor field as a "protein_coding_gene", for a remaining total of 27,416 genes. We make this dataset available as zipped archive AdditionalFile10_TAIR10_GFF3_genes.protein_coding_genes.gff.zip

Degree variation and sampling uniformity

The series of examples below considers a progression of source and target degree variations using the same overall vertex configuration. We consider the same 4-source, 5-target configuration as in Figure 4B. We begin with a series of symmetric degree variations in examples sym1, sym2, and sym3. The sym1 example has edge configurations determined by giving an out-degree of two to all sources, and keeping target in-degrees as close to two as possible. This configuration has eight edges, and there are 204 possible such labeled graphs. The sym2 and sym3 examples increase this source out-degree uniformly to three and four, keeping target in-degrees as close to three and four as possible, respectively (at an out-degree of five for all sources and four for all targets, there is only one possible edge configuration as vertex degrees are saturated). In contrast, the progression of graphs asym1-asym4 provides a series of asymmetric variations with different examples of uneven source and target degree distributions. The outcomes for both progressions, plotted below, emphasize the trend that WaRSwap sampling improvements over FANMOD are seen as the number of possible graphs increases. As in Figure 4, each algorithm is run 10,000 times per example.

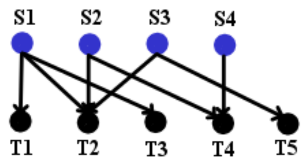

4-Source, 5-Target Graph
(This illustration displays the edge configuration of asym1, the graph in Figure 4B)

Graph Name		***S1*** / ***T1***	***S2*** / ***T2***	***S3*** / ***T3***	***S4*** / ***T4***	***T5***	***# Edges***	***# Possible Graphs***
**sym1**	*Source Out-Degrees*	2	2	2	2		8	204
	Target In-Degrees	2	2	2	1	1		
**sym2**	*Source Out-Degrees*	3	3	3	3		12	204
	Target In-Degrees	3	2	3	2	2		
**sym3**	*Source Out-Degrees*	4	4	4	4		16	24
	Target In-Degrees	3	3	4	3	3		
**asym1**	*Source Out-Degrees*	3	2	2	1		8	58
	Target In-Degrees	1	3	1	2	1		
**asym2**	*Source Out-Degrees*	4	3	2	1		10	49
	Target In-Degrees	2	3	2	2	1		
**asym3**	*Source Out-Degrees*	4	3	2	1		10	8
	Target In-Degrees	2	4	2	1	1		
**asym4**	*Source Out-Degrees*	4	4	1	1		10	4
	Target In-Degrees	1	3	3	2	1		

Symmetric variation example outcomes (sym1, sym2, sym3):

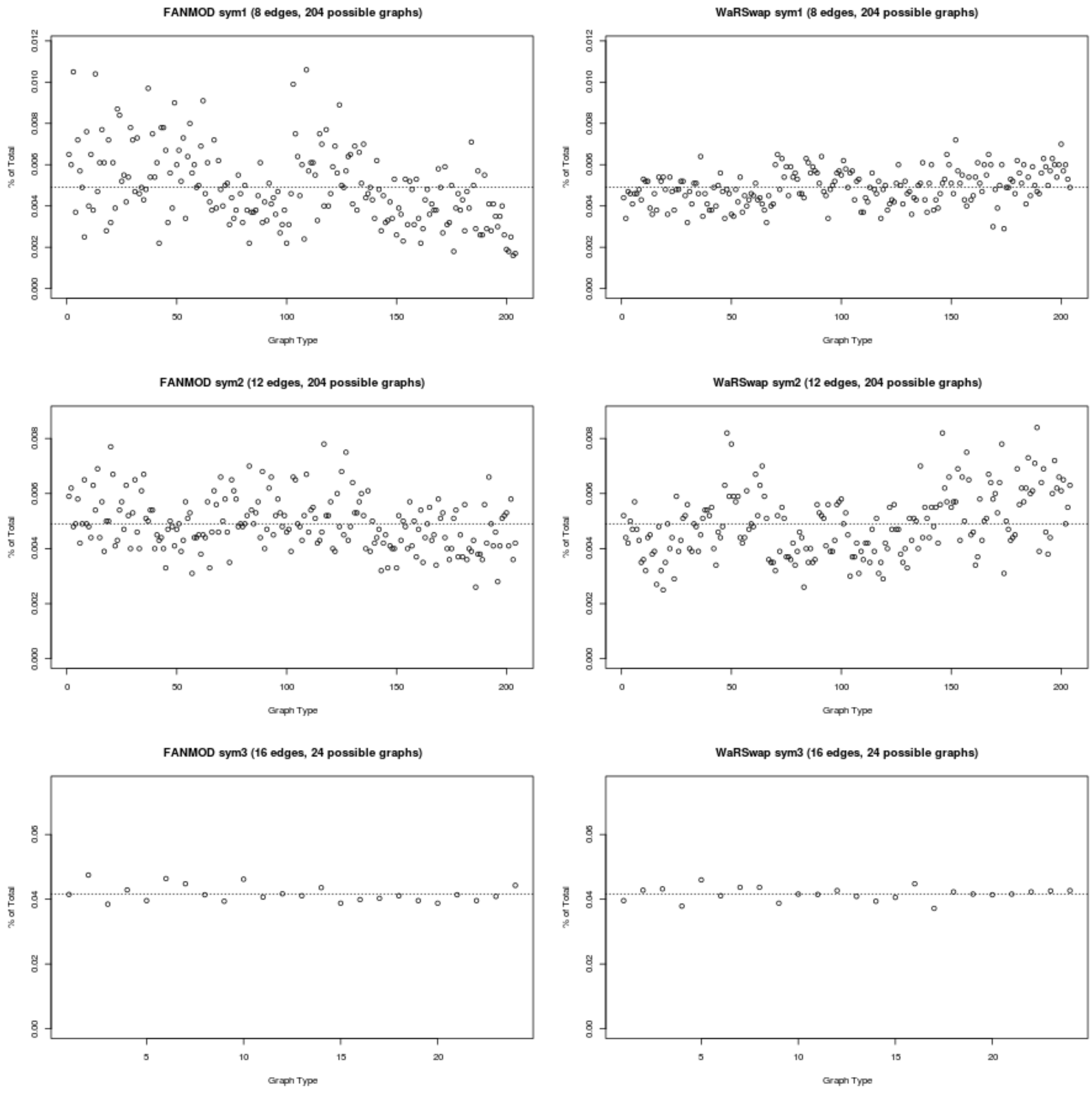

Asymmetric variation example outcomes (asym1, asym2, asym3, asym4):

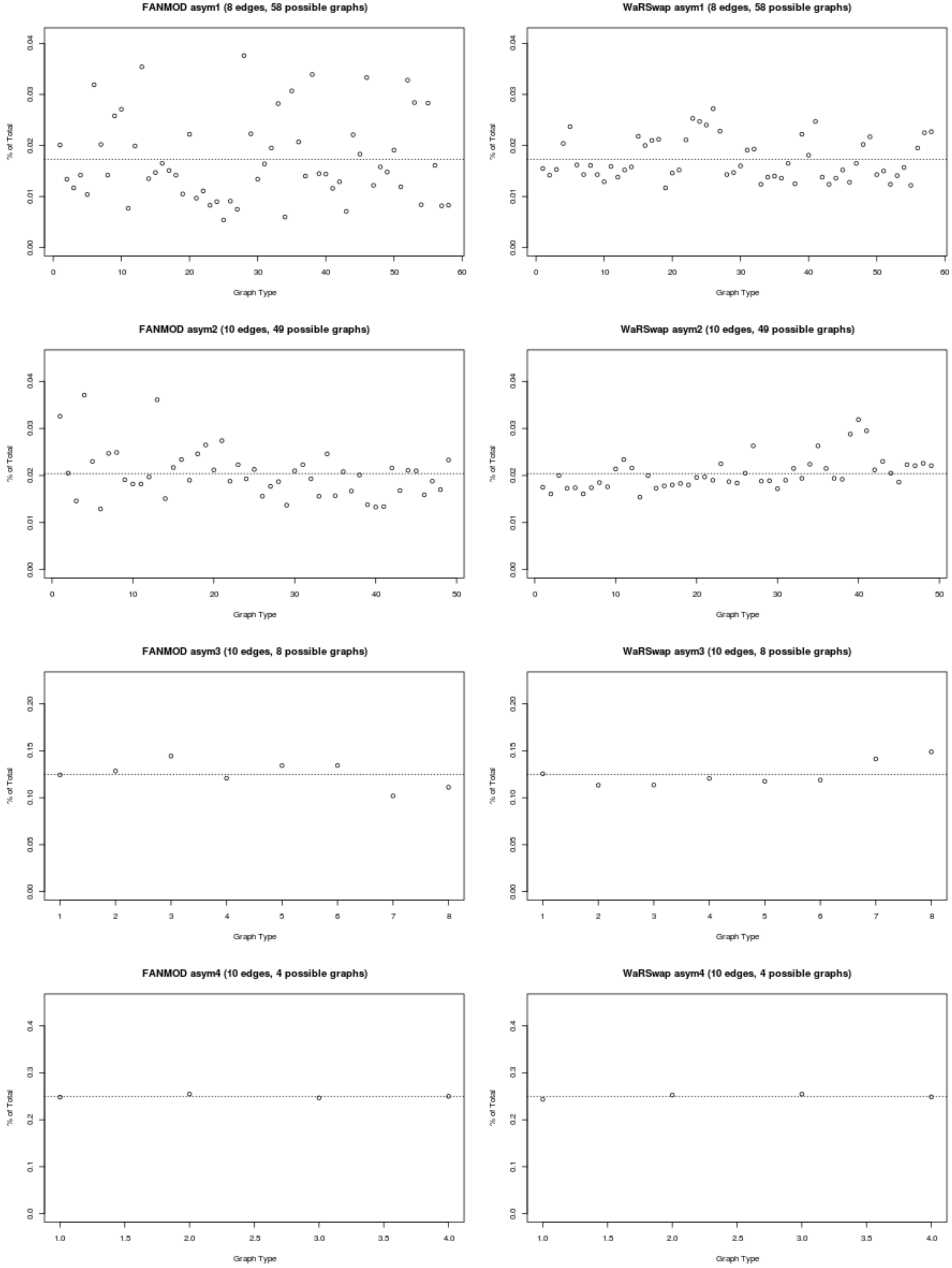

Supplement: Additional file 1 — Main Supplement. Main supplement, contains table of contents for this and all additional files, and detailed description of all additional files. Contains supplementary figures and text. [file gb-2013-14-8-r85-S1.PDF]
