# Supplementary material for: Sustained-input switches for transcription factors and microRNAs are central building blocks of eukaryotic gene circuits
Source: Genome Biol. 2013 Aug 23;14(8):R85. doi: 10.1186/gb-2013-14-8-r85 (PMC4054853; doi:10.1186/gb-2013-14-8-r85)
Supplement: Additional file 5 — HTML Browsable Motif Output. Zipped folder containing all WaRSwap and FANMOD motif output, viewable in a web browser. [file gb-2013-14-8-r85-S5.ZIP › HTML_browsable_motif_output/FANMOD_ath_tair10/sigs_FANMOD_TAIR10-2500.pvals.heatmaps.html/motif_id_14_010111002_tftype_ath_upstream_-3000_0.html]

```
BG_MODEL = FANMOD
MOTIF_ID = 14_010111002
TF_TYPE = ath
UPSTREAM = -3000_0


PVals
FNR = 0.2	FNR = 0.4	FNR = 0.6	FNR = 0.8
deltaG = 60	0.005	0.012	0.102	0.872
deltaG = 70	0	0.002	0.01	0.797
deltaG = 80	0	0.003	0.012	0.656

ZScores
FNR = 0.2	FNR = 0.4	FNR = 0.6	FNR = 0.8
deltaG = 60	5.621	3.479	1.642	-0.848
deltaG = 70	7.784	4.636	2.228	-0.863
deltaG = 80	8.387	4.929	2.632	-0.833

StDevs
FNR = 0.2	FNR = 0.4	FNR = 0.6	FNR = 0.8
deltaG = 60	89.022	130.529	228.817	277.702
deltaG = 70	67.649	108.895	205.474	275.011
deltaG = 80	39.953	66.787	116.842	171.086
```
