# Supplementary material for: Sustained-input switches for transcription factors and microRNAs are central building blocks of eukaryotic gene circuits
Source: Genome Biol. 2013 Aug 23;14(8):R85. doi: 10.1186/gb-2013-14-8-r85 (PMC4054853; doi:10.1186/gb-2013-14-8-r85)
Supplement: Additional file 5 — HTML Browsable Motif Output. Zipped folder containing all WaRSwap and FANMOD motif output, viewable in a web browser. [file gb-2013-14-8-r85-S5.ZIP › HTML_browsable_motif_output/FANMOD_ath_tair10/sigs_FANMOD_TAIR10-2500.pvals.heatmaps.html/motif_id_166_001101101_tftype_ath_upstream_-3000_0.html]

```
BG_MODEL = FANMOD
MOTIF_ID = 166_001101101
TF_TYPE = ath
UPSTREAM = -3000_0


PVals
FNR = 0.2	FNR = 0.4	FNR = 0.6	FNR = 0.8
deltaG = 60	0.383	0.274	0.037	0.495
deltaG = 70	0.309	0.208	0.033	0.452
deltaG = 80	0.246	0.214	0.006	0.348

ZScores
FNR = 0.2	FNR = 0.4	FNR = 0.6	FNR = 0.8
deltaG = 60	0.378	0.432	1.864	-0.666
deltaG = 70	0.724	0.633	2.001	-0.643
deltaG = 80	0.935	0.538	3.277	-0.47

StDevs
FNR = 0.2	FNR = 0.4	FNR = 0.6	FNR = 0.8
deltaG = 60	9.646	6.959	4.033	1.2
deltaG = 70	8.467	6.023	3.421	0.997
deltaG = 80	8.014	4.976	2.606	1.151
```
