# Supplementary material for: Sustained-input switches for transcription factors and microRNAs are central building blocks of eukaryotic gene circuits
Source: Genome Biol. 2013 Aug 23;14(8):R85. doi: 10.1186/gb-2013-14-8-r85 (PMC4054853; doi:10.1186/gb-2013-14-8-r85)
Supplement: Additional file 5 — HTML Browsable Motif Output. Zipped folder containing all WaRSwap and FANMOD motif output, viewable in a web browser. [file gb-2013-14-8-r85-S5.ZIP › HTML_browsable_motif_output/FANMOD_ath_tair10/sigs_FANMOD_TAIR10-2500.pvals.heatmaps.html/motif_id_38_000101101_tftype_ath_upstream_-3000_0.html]

```
BG_MODEL = FANMOD
MOTIF_ID = 38_000101101
TF_TYPE = ath
UPSTREAM = -3000_0


PVals
FNR = 0.2	FNR = 0.4	FNR = 0.6	FNR = 0.8
deltaG = 60	0.112	0.134	0.34	0.004
deltaG = 70	0.168	0.115	0.106	0.001
deltaG = 80	0.053	0.018	0.102	0

ZScores
FNR = 0.2	FNR = 0.4	FNR = 0.6	FNR = 0.8
deltaG = 60	1.172	1.038	0.353	2.838
deltaG = 70	0.936	1.166	1.183	3.344
deltaG = 80	1.557	2.064	1.233	3.435

StDevs
FNR = 0.2	FNR = 0.4	FNR = 0.6	FNR = 0.8
deltaG = 60	17.289	11.246	8.332	4.626
deltaG = 70	16.39	10.406	7.581	4.165
deltaG = 80	13.454	9.364	6.849	3.852
```
