# Supplementary material for: Sustained-input switches for transcription factors and microRNAs are central building blocks of eukaryotic gene circuits
Source: Genome Biol. 2013 Aug 23;14(8):R85. doi: 10.1186/gb-2013-14-8-r85 (PMC4054853; doi:10.1186/gb-2013-14-8-r85)
Supplement: Additional file 5 — HTML Browsable Motif Output. Zipped folder containing all WaRSwap and FANMOD motif output, viewable in a web browser. [file gb-2013-14-8-r85-S5.ZIP › HTML_browsable_motif_output/FANMOD_ath_tair10/sigs_FANMOD_TAIR10-2500.pvals.heatmaps.html/motif_id_46_011101001_tftype_ath_upstream_-3000_0.html]

```
BG_MODEL = FANMOD
MOTIF_ID = 46_011101001
TF_TYPE = ath
UPSTREAM = -3000_0


PVals
FNR = 0.2	FNR = 0.4	FNR = 0.6	FNR = 0.8
deltaG = 60	0.216	0.035	0.005	0.811
deltaG = 70	0.219	0.031	0.004	0.818
deltaG = 80	0.206	0.039	0.003	0.827

ZScores
FNR = 0.2	FNR = 0.4	FNR = 0.6	FNR = 0.8
deltaG = 60	0.767	1.785	2.583	-1.251
deltaG = 70	0.742	1.823	2.543	-1.29
deltaG = 80	0.811	1.796	2.607	-1.281

StDevs
FNR = 0.2	FNR = 0.4	FNR = 0.6	FNR = 0.8
deltaG = 60	264.722	286.037	96.881	17.216
deltaG = 70	260.883	284.408	97.739	17.234
deltaG = 80	260.47	290.007	97.778	17.144
```
