# Supplementary material for: Sustained-input switches for transcription factors and microRNAs are central building blocks of eukaryotic gene circuits
Source: Genome Biol. 2013 Aug 23;14(8):R85. doi: 10.1186/gb-2013-14-8-r85 (PMC4054853; doi:10.1186/gb-2013-14-8-r85)
Supplement: Additional file 5 — HTML Browsable Motif Output. Zipped folder containing all WaRSwap and FANMOD motif output, viewable in a web browser. [file gb-2013-14-8-r85-S5.ZIP › HTML_browsable_motif_output/FANMOD_ath_tair9/sigs_fanmodm-2000.pvals.heatmaps.html/motif_id_12_000001100_tftype_ath_upstream_-2000_0.html]

```
BG_MODEL = FANMOD
MOTIF_ID = 12_000001100
TF_TYPE = ath
UPSTREAM = -2000_0


PVals
FN_0.2	FN_0.4	FN_0.6	FN_0.8
dg_60.genes	0.827	0.972	0.814	0
dg_70.genes	0.828	0.97	0.821	0
dg_80.genes	0.841	0.976	0.789	0

ZScores
FN_0.2	FN_0.4	FN_0.6	FN_0.8
dg_60.genes	-0.926	-1.856	-0.893	2.203
dg_70.genes	-0.96	-1.9	-0.921	2.163
dg_80.genes	-0.995	-1.955	-0.852	2.213

StDevs
FN_0.2	FN_0.4	FN_0.6	FN_0.8
dg_60.genes	33.086	47.108	38.347	9.99
dg_70.genes	32.961	47.613	37.948	10.018
dg_80.genes	31.79	45.273	39.451	9.813
```
