# Supplementary material for: Sustained-input switches for transcription factors and microRNAs are central building blocks of eukaryotic gene circuits
Source: Genome Biol. 2013 Aug 23;14(8):R85. doi: 10.1186/gb-2013-14-8-r85 (PMC4054853; doi:10.1186/gb-2013-14-8-r85)
Supplement: Additional file 5 — HTML Browsable Motif Output. Zipped folder containing all WaRSwap and FANMOD motif output, viewable in a web browser. [file gb-2013-14-8-r85-S5.ZIP › HTML_browsable_motif_output/FANMOD_ath_tair9/sigs_fanmodm-2000.pvals.heatmaps.html/motif_id_12_000001101_tftype_ath_upstream_-1000_0.html]

```
BG_MODEL = FANMOD
MOTIF_ID = 12_000001101
TF_TYPE = ath
UPSTREAM = -1000_0


PVals
FN_0.2	FN_0.4	FN_0.6	FN_0.8
dg_60.genes	0.566	0.849	0	0
dg_70.genes	0.111	0.925	0	0
dg_80.genes	0.35	0.958	0	0

ZScores
FN_0.2	FN_0.4	FN_0.6	FN_0.8
dg_60.genes	-0.155	-1.076	2.117	1.202
dg_70.genes	1.172	-1.465	2.292	1.133
dg_80.genes	0.323	-1.776	1.808	0.935

StDevs
FN_0.2	FN_0.4	FN_0.6	FN_0.8
dg_60.genes	10.47	10.367	7.828	6.119
dg_70.genes	8.352	8.969	6.861	4.73
dg_80.genes	7.103	7.835	5.479	4.548
```
