# Supplementary material for: Sustained-input switches for transcription factors and microRNAs are central building blocks of eukaryotic gene circuits
Source: Genome Biol. 2013 Aug 23;14(8):R85. doi: 10.1186/gb-2013-14-8-r85 (PMC4054853; doi:10.1186/gb-2013-14-8-r85)
Supplement: Additional file 5 — HTML Browsable Motif Output. Zipped folder containing all WaRSwap and FANMOD motif output, viewable in a web browser. [file gb-2013-14-8-r85-S5.ZIP › HTML_browsable_motif_output/FANMOD_ath_tair9/sigs_fanmodm-2000.pvals.heatmaps.html/motif_id_12_000100011_tftype_ath_upstream_-1000_0.html]

```
BG_MODEL = FANMOD
MOTIF_ID = 12_000100011
TF_TYPE = ath
UPSTREAM = -1000_0


PVals
FN_0.2	FN_0.4	FN_0.6	FN_0.8
dg_60.genes	0.196	0.726	0.002	0
dg_70.genes	0.145	0.706	0.002	0
dg_80.genes	0.076	0.478	0.006	0

ZScores
FN_0.2	FN_0.4	FN_0.6	FN_0.8
dg_60.genes	0.928	-0.616	2.279	1.33
dg_70.genes	1.154	-0.543	2.218	1.229
dg_80.genes	1.634	-0.015	1.731	1.075

StDevs
FN_0.2	FN_0.4	FN_0.6	FN_0.8
dg_60.genes	25.775	18.888	8.804	2.467
dg_70.genes	24.164	18.852	8.429	2.315
dg_80.genes	17.345	14.239	6.585	1.918
```
