# Supplementary material for: Sustained-input switches for transcription factors and microRNAs are central building blocks of eukaryotic gene circuits
Source: Genome Biol. 2013 Aug 23;14(8):R85. doi: 10.1186/gb-2013-14-8-r85 (PMC4054853; doi:10.1186/gb-2013-14-8-r85)
Supplement: Additional file 5 — HTML Browsable Motif Output. Zipped folder containing all WaRSwap and FANMOD motif output, viewable in a web browser. [file gb-2013-14-8-r85-S5.ZIP › HTML_browsable_motif_output/FANMOD_ath_tair9/sigs_fanmodm-2000.pvals.heatmaps.html/motif_id_12_001100001_tftype_ath_upstream_-1000_0.html]

```
BG_MODEL = FANMOD
MOTIF_ID = 12_001100001
TF_TYPE = ath
UPSTREAM = -1000_0


PVals
FN_0.2	FN_0.4	FN_0.6	FN_0.8
dg_60.genes	0.62	0.705	0.535	0
dg_70.genes	0.634	0.707	0.53	0
dg_80.genes	0.628	0.707	0.552	0

ZScores
FN_0.2	FN_0.4	FN_0.6	FN_0.8
dg_60.genes	-0.35	-0.584	-0.074	3.221
dg_70.genes	-0.368	-0.557	-0.066	3.267
dg_80.genes	-0.35	-0.579	-0.13	3.221

StDevs
FN_0.2	FN_0.4	FN_0.6	FN_0.8
dg_60.genes	238.724	233.697	87.274	16.562
dg_70.genes	235.509	225.146	86.186	16.23
dg_80.genes	241.106	230.815	87.417	16.789
```
