# Supplementary material for: Sustained-input switches for transcription factors and microRNAs are central building blocks of eukaryotic gene circuits
Source: Genome Biol. 2013 Aug 23;14(8):R85. doi: 10.1186/gb-2013-14-8-r85 (PMC4054853; doi:10.1186/gb-2013-14-8-r85)
Supplement: Additional file 5 — HTML Browsable Motif Output. Zipped folder containing all WaRSwap and FANMOD motif output, viewable in a web browser. [file gb-2013-14-8-r85-S5.ZIP › HTML_browsable_motif_output/FANMOD_ath_tair9/sigs_fanmodm-2000.pvals.heatmaps.html/motif_id_12_001100001_tftype_ath_upstream_-2000_0.html]

```
BG_MODEL = FANMOD
MOTIF_ID = 12_001100001
TF_TYPE = ath
UPSTREAM = -2000_0


PVals
FN_0.2	FN_0.4	FN_0.6	FN_0.8
dg_60.genes	0.627	0.83	0.704	0
dg_70.genes	0.638	0.843	0.706	0
dg_80.genes	0.648	0.831	0.693	0

ZScores
FN_0.2	FN_0.4	FN_0.6	FN_0.8
dg_60.genes	-0.342	-0.932	-0.548	4.086
dg_70.genes	-0.354	-0.995	-0.565	3.91
dg_80.genes	-0.39	-1.009	-0.521	4.016

StDevs
FN_0.2	FN_0.4	FN_0.6	FN_0.8
dg_60.genes	192.301	262.421	126.788	26.156
dg_70.genes	194.886	265.556	126.47	26.976
dg_80.genes	187.649	253.942	129.367	26.293
```
