# Supplementary material for: Sustained-input switches for transcription factors and microRNAs are central building blocks of eukaryotic gene circuits
Source: Genome Biol. 2013 Aug 23;14(8):R85. doi: 10.1186/gb-2013-14-8-r85 (PMC4054853; doi:10.1186/gb-2013-14-8-r85)
Supplement: Additional file 5 — HTML Browsable Motif Output. Zipped folder containing all WaRSwap and FANMOD motif output, viewable in a web browser. [file gb-2013-14-8-r85-S5.ZIP › HTML_browsable_motif_output/FANMOD_ath_tair9/sigs_fanmodm-2000.pvals.heatmaps.html/motif_id_12_001100002_tftype_ath_upstream_-2000_0.html]

```
BG_MODEL = FANMOD
MOTIF_ID = 12_001100002
TF_TYPE = ath
UPSTREAM = -2000_0


PVals
FN_0.2	FN_0.4	FN_0.6	FN_0.8
dg_60.genes	0.427	0.767	0.8	0
dg_70.genes	0.436	0.784	0.805	0
dg_80.genes	0.436	0.778	0.78	0

ZScores
FN_0.2	FN_0.4	FN_0.6	FN_0.8
dg_60.genes	0.141	-0.72	-0.832	2.877
dg_70.genes	0.125	-0.789	-0.843	2.81
dg_80.genes	0.118	-0.791	-0.793	2.897

StDevs
FN_0.2	FN_0.4	FN_0.6	FN_0.8
dg_60.genes	28445.605	36663.839	17544.269	3095.959
dg_70.genes	28735.521	37175.678	17501.043	3116.851
dg_80.genes	27558.794	35360.003	17845.2	3038.227
```
