# Supplementary material for: Sustained-input switches for transcription factors and microRNAs are central building blocks of eukaryotic gene circuits
Source: Genome Biol. 2013 Aug 23;14(8):R85. doi: 10.1186/gb-2013-14-8-r85 (PMC4054853; doi:10.1186/gb-2013-14-8-r85)
Supplement: Additional file 5 — HTML Browsable Motif Output. Zipped folder containing all WaRSwap and FANMOD motif output, viewable in a web browser. [file gb-2013-14-8-r85-S5.ZIP › HTML_browsable_motif_output/FANMOD_ath_tair9/sigs_fanmodm-2000.pvals.heatmaps.html/motif_id_166_010100110_tftype_ath_upstream_-2000_0.html]

```
BG_MODEL = FANMOD
MOTIF_ID = 166_010100110
TF_TYPE = ath
UPSTREAM = -2000_0


PVals
FN_0.2	FN_0.4	FN_0.6	FN_0.8
dg_60.genes	0.019	0.196	0	0.074
dg_70.genes	0.017	0.2	0.001	0.067
dg_80.genes	0.018	0.188	0.001	0.065

ZScores
FN_0.2	FN_0.4	FN_0.6	FN_0.8
dg_60.genes	2.185	0.81	3.37	-0.261
dg_70.genes	2.176	0.806	3.391	-0.249
dg_80.genes	2.133	0.84	3.361	-0.25

StDevs
FN_0.2	FN_0.4	FN_0.6	FN_0.8
dg_60.genes	22.106	13.358	5.156	0.349
dg_70.genes	21.799	13.542	5.144	0.348
dg_80.genes	22.024	13.691	5.085	0.298
```
