# Supplementary material for: Sustained-input switches for transcription factors and microRNAs are central building blocks of eukaryotic gene circuits
Source: Genome Biol. 2013 Aug 23;14(8):R85. doi: 10.1186/gb-2013-14-8-r85 (PMC4054853; doi:10.1186/gb-2013-14-8-r85)
Supplement: Additional file 5 — HTML Browsable Motif Output. Zipped folder containing all WaRSwap and FANMOD motif output, viewable in a web browser. [file gb-2013-14-8-r85-S5.ZIP › HTML_browsable_motif_output/FANMOD_ath_tair9/sigs_fanmodm-2000.pvals.heatmaps.html/motif_id_166_010100110_tftype_ath_upstream_-3000_0.html]

```
BG_MODEL = FANMOD
MOTIF_ID = 166_010100110
TF_TYPE = ath
UPSTREAM = -3000_0


PVals
FN_0.2	FN_0.4	FN_0.6	FN_0.8
dg_60.genes	0.072	0.323	0	0.102
dg_70.genes	0.076	0.302	0	0.107
dg_80.genes	0.083	0.323	0	0.114

ZScores
FN_0.2	FN_0.4	FN_0.6	FN_0.8
dg_60.genes	1.468	0.404	4.09	0.733
dg_70.genes	1.409	0.481	4.096	0.727
dg_80.genes	1.414	0.4	4.078	0.72

StDevs
FN_0.2	FN_0.4	FN_0.6	FN_0.8
dg_60.genes	20.089	13.911	5.334	0.76
dg_70.genes	21.316	14	5.284	0.755
dg_80.genes	20.888	13.654	5.329	0.756
```
