# Supplementary material for: Sustained-input switches for transcription factors and microRNAs are central building blocks of eukaryotic gene circuits
Source: Genome Biol. 2013 Aug 23;14(8):R85. doi: 10.1186/gb-2013-14-8-r85 (PMC4054853; doi:10.1186/gb-2013-14-8-r85)
Supplement: Additional file 5 — HTML Browsable Motif Output. Zipped folder containing all WaRSwap and FANMOD motif output, viewable in a web browser. [file gb-2013-14-8-r85-S5.ZIP › HTML_browsable_motif_output/FANMOD_ath_tair9/sigs_fanmodm-2000.pvals.heatmaps.html/motif_id_238_011101110_tftype_ath_upstream_-1000_0.html]

```
BG_MODEL = FANMOD
MOTIF_ID = 238_011101110
TF_TYPE = ath
UPSTREAM = -1000_0


PVals
FN_0.2	FN_0.4	FN_0.6	FN_0.8
dg_60.genes	0.042	0.008	0.118	1
dg_70.genes	0.031	0.009	0.124	1
dg_80.genes	0.036	0.007	0.112	1

ZScores
FN_0.2	FN_0.4	FN_0.6	FN_0.8
dg_60.genes	1.759	2.594	-0.348	NA
dg_70.genes	1.792	2.572	-0.368	NA
dg_80.genes	1.792	2.656	-0.341	NA

StDevs
FN_0.2	FN_0.4	FN_0.6	FN_0.8
dg_60.genes	20.668	4.773	0.369	NA
dg_70.genes	20.309	4.771	0.354	NA
dg_80.genes	20.198	4.611	0.36	NA
```
