# Supplementary material for: Sustained-input switches for transcription factors and microRNAs are central building blocks of eukaryotic gene circuits
Source: Genome Biol. 2013 Aug 23;14(8):R85. doi: 10.1186/gb-2013-14-8-r85 (PMC4054853; doi:10.1186/gb-2013-14-8-r85)
Supplement: Additional file 5 — HTML Browsable Motif Output. Zipped folder containing all WaRSwap and FANMOD motif output, viewable in a web browser. [file gb-2013-14-8-r85-S5.ZIP › HTML_browsable_motif_output/FANMOD_ath_tair9/sigs_fanmodm-2000.pvals.heatmaps.html/motif_id_238_011101110_tftype_ath_upstream_-2000_0.html]

```
BG_MODEL = FANMOD
MOTIF_ID = 238_011101110
TF_TYPE = ath
UPSTREAM = -2000_0


PVals
FN_0.2	FN_0.4	FN_0.6	FN_0.8
dg_60.genes	0.567	0.002	0.137	1
dg_70.genes	0.577	0.006	0.155	1
dg_80.genes	0.585	0.003	0.147	1

ZScores
FN_0.2	FN_0.4	FN_0.6	FN_0.8
dg_60.genes	-0.22	2.815	0.841	NA
dg_70.genes	-0.241	2.762	0.772	NA
dg_80.genes	-0.229	2.851	0.791	NA

StDevs
FN_0.2	FN_0.4	FN_0.6	FN_0.8
dg_60.genes	22.86	9.63	2.092	NA
dg_70.genes	22.014	9.978	2.176	NA
dg_80.genes	22.413	9.67	2.153	NA
```
