# Supplementary material for: Sustained-input switches for transcription factors and microRNAs are central building blocks of eukaryotic gene circuits
Source: Genome Biol. 2013 Aug 23;14(8):R85. doi: 10.1186/gb-2013-14-8-r85 (PMC4054853; doi:10.1186/gb-2013-14-8-r85)
Supplement: Additional file 5 — HTML Browsable Motif Output. Zipped folder containing all WaRSwap and FANMOD motif output, viewable in a web browser. [file gb-2013-14-8-r85-S5.ZIP › HTML_browsable_motif_output/FANMOD_ath_tair9/sigs_fanmodm-2000.pvals.heatmaps.html/motif_id_238_011101110_tftype_ath_upstream_-3000_0.html]

```
BG_MODEL = FANMOD
MOTIF_ID = 238_011101110
TF_TYPE = ath
UPSTREAM = -3000_0


PVals
FN_0.2	FN_0.4	FN_0.6	FN_0.8
dg_60.genes	0.203	0.001	0.029	0.001
dg_70.genes	0.192	0.001	0.027	0.001
dg_80.genes	0.209	0.001	0.032	0.001

ZScores
FN_0.2	FN_0.4	FN_0.6	FN_0.8
dg_60.genes	0.849	3.347	2.032	-0.022
dg_70.genes	0.85	3.273	2.082	-0.032
dg_80.genes	0.8	3.404	1.989	-0.022

StDevs
FN_0.2	FN_0.4	FN_0.6	FN_0.8
dg_60.genes	24.383	12.244	2.791	0.022
dg_70.genes	24.371	12.258	2.749	0.032
dg_80.genes	25.104	12.052	2.827	0.022
```
