# Supplementary material for: Sustained-input switches for transcription factors and microRNAs are central building blocks of eukaryotic gene circuits
Source: Genome Biol. 2013 Aug 23;14(8):R85. doi: 10.1186/gb-2013-14-8-r85 (PMC4054853; doi:10.1186/gb-2013-14-8-r85)
Supplement: Additional file 5 — HTML Browsable Motif Output. Zipped folder containing all WaRSwap and FANMOD motif output, viewable in a web browser. [file gb-2013-14-8-r85-S5.ZIP › HTML_browsable_motif_output/FANMOD_ath_tair9/sigs_fanmodm-2000.pvals.heatmaps.html/motif_id_36_000100100_tftype_ath_upstream_-2000_0.html]

```
BG_MODEL = FANMOD
MOTIF_ID = 36_000100100
TF_TYPE = ath
UPSTREAM = -2000_0


PVals
FN_0.2	FN_0.4	FN_0.6	FN_0.8
dg_60.genes	0.842	0.986	1	0
dg_70.genes	0.846	0.983	1	0
dg_80.genes	0.836	0.983	1	0

ZScores
FN_0.2	FN_0.4	FN_0.6	FN_0.8
dg_60.genes	-1.048	-2.144	-5.737	3.404
dg_70.genes	-1.057	-2.067	-5.885	3.359
dg_80.genes	-1.013	-2.159	-5.673	3.403

StDevs
FN_0.2	FN_0.4	FN_0.6	FN_0.8
dg_60.genes	21.741	24.984	13.667	3.893
dg_70.genes	20.9	25.482	13.36	3.959
dg_80.genes	21.521	24.959	13.704	3.887
```
