# Supplementary material for: Sustained-input switches for transcription factors and microRNAs are central building blocks of eukaryotic gene circuits
Source: Genome Biol. 2013 Aug 23;14(8):R85. doi: 10.1186/gb-2013-14-8-r85 (PMC4054853; doi:10.1186/gb-2013-14-8-r85)
Supplement: Additional file 5 — HTML Browsable Motif Output. Zipped folder containing all WaRSwap and FANMOD motif output, viewable in a web browser. [file gb-2013-14-8-r85-S5.ZIP › HTML_browsable_motif_output/FANMOD_ath_tair9/sigs_fanmodm-2000.pvals.heatmaps.html/motif_id_36_000100101_tftype_ath_upstream_-1000_0.html]

```
BG_MODEL = FANMOD
MOTIF_ID = 36_000100101
TF_TYPE = ath
UPSTREAM = -1000_0


PVals
FN_0.2	FN_0.4	FN_0.6	FN_0.8
dg_60.genes	0.989	1	0.021	0
dg_70.genes	0.958	1	0.005	0
dg_80.genes	0.982	1	0.009	0

ZScores
FN_0.2	FN_0.4	FN_0.6	FN_0.8
dg_60.genes	-2.35	-3.172	1.819	0.82
dg_70.genes	-1.697	-3.5	2.292	0.792
dg_80.genes	-2.076	-3.949	1.907	0.683

StDevs
FN_0.2	FN_0.4	FN_0.6	FN_0.8
dg_60.genes	11.292	6.99	2.842	1.335
dg_70.genes	9.95	6.524	2.829	1.352
dg_80.genes	8.675	5.84	1.805	0.714
```
