# Supplementary material for: Sustained-input switches for transcription factors and microRNAs are central building blocks of eukaryotic gene circuits
Source: Genome Biol. 2013 Aug 23;14(8):R85. doi: 10.1186/gb-2013-14-8-r85 (PMC4054853; doi:10.1186/gb-2013-14-8-r85)
Supplement: Additional file 5 — HTML Browsable Motif Output. Zipped folder containing all WaRSwap and FANMOD motif output, viewable in a web browser. [file gb-2013-14-8-r85-S5.ZIP › HTML_browsable_motif_output/FANMOD_ath_tair9/sigs_fanmodm-2000.pvals.heatmaps.html/motif_id_36_001001001_tftype_ath_upstream_-1000_0.html]

```
BG_MODEL = FANMOD
MOTIF_ID = 36_001001001
TF_TYPE = ath
UPSTREAM = -1000_0


PVals
FN_0.2	FN_0.4	FN_0.6	FN_0.8
dg_60.genes	0.639	1	1	0
dg_70.genes	0.641	1	1	0
dg_80.genes	0.648	1	1	0

ZScores
FN_0.2	FN_0.4	FN_0.6	FN_0.8
dg_60.genes	-0.418	-3.668	-4.029	4.374
dg_70.genes	-0.421	-3.576	-4.061	4.5
dg_80.genes	-0.436	-3.77	-4.151	4.363

StDevs
FN_0.2	FN_0.4	FN_0.6	FN_0.8
dg_60.genes	89.355	99.982	25.631	10.026
dg_70.genes	91.98	102.189	25.581	9.42
dg_80.genes	94.119	98.042	25.571	9.542
```
