# Supplementary material for: Sustained-input switches for transcription factors and microRNAs are central building blocks of eukaryotic gene circuits
Source: Genome Biol. 2013 Aug 23;14(8):R85. doi: 10.1186/gb-2013-14-8-r85 (PMC4054853; doi:10.1186/gb-2013-14-8-r85)
Supplement: Additional file 5 — HTML Browsable Motif Output. Zipped folder containing all WaRSwap and FANMOD motif output, viewable in a web browser. [file gb-2013-14-8-r85-S5.ZIP › HTML_browsable_motif_output/FANMOD_ath_tair9/sigs_fanmodm-2000.pvals.heatmaps.html/motif_id_36_001001001_tftype_ath_upstream_-2000_0.html]

```
BG_MODEL = FANMOD
MOTIF_ID = 36_001001001
TF_TYPE = ath
UPSTREAM = -2000_0


PVals
FN_0.2	FN_0.4	FN_0.6	FN_0.8
dg_60.genes	0.835	0.433	1	0
dg_70.genes	0.84	0.418	1	0
dg_80.genes	0.843	0.439	1	0

ZScores
FN_0.2	FN_0.4	FN_0.6	FN_0.8
dg_60.genes	-1.002	0.165	-4.146	5.404
dg_70.genes	-1.015	0.172	-4.039	5.311
dg_80.genes	-1.017	0.143	-4.009	5.293

StDevs
FN_0.2	FN_0.4	FN_0.6	FN_0.8
dg_60.genes	67.488	127.691	43.663	15.94
dg_70.genes	65.723	128.658	45.369	15.874
dg_80.genes	67.182	128.522	44.719	15.708
```
