# Supplementary material for: Sustained-input switches for transcription factors and microRNAs are central building blocks of eukaryotic gene circuits
Source: Genome Biol. 2013 Aug 23;14(8):R85. doi: 10.1186/gb-2013-14-8-r85 (PMC4054853; doi:10.1186/gb-2013-14-8-r85)
Supplement: Additional file 5 — HTML Browsable Motif Output. Zipped folder containing all WaRSwap and FANMOD motif output, viewable in a web browser. [file gb-2013-14-8-r85-S5.ZIP › HTML_browsable_motif_output/FANMOD_ath_tair9/sigs_fanmodm-2000.pvals.heatmaps.html/motif_id_36_001001002_tftype_ath_upstream_-1000_0.html]

```
BG_MODEL = FANMOD
MOTIF_ID = 36_001001002
TF_TYPE = ath
UPSTREAM = -1000_0


PVals
FN_0.2	FN_0.4	FN_0.6	FN_0.8
dg_60.genes	0.507	1	1	0
dg_70.genes	0.519	1	1	0
dg_80.genes	0.534	1	1	0

ZScores
FN_0.2	FN_0.4	FN_0.6	FN_0.8
dg_60.genes	-0.091	-3.865	-4.809	5.367
dg_70.genes	-0.101	-3.781	-4.864	5.504
dg_80.genes	-0.118	-3.904	-4.846	5.471

StDevs
FN_0.2	FN_0.4	FN_0.6	FN_0.8
dg_60.genes	12849.596	13272.577	2997.213	576.367
dg_70.genes	12881.262	13488.571	2956.388	564.643
dg_80.genes	13254.471	13097.515	2951.584	563.239
```
