# Supplementary material for: Sustained-input switches for transcription factors and microRNAs are central building blocks of eukaryotic gene circuits
Source: Genome Biol. 2013 Aug 23;14(8):R85. doi: 10.1186/gb-2013-14-8-r85 (PMC4054853; doi:10.1186/gb-2013-14-8-r85)
Supplement: Additional file 5 — HTML Browsable Motif Output. Zipped folder containing all WaRSwap and FANMOD motif output, viewable in a web browser. [file gb-2013-14-8-r85-S5.ZIP › HTML_browsable_motif_output/FANMOD_ath_tair9/sigs_fanmodm-2000.pvals.heatmaps.html/motif_id_36_001001002_tftype_ath_upstream_-2000_0.html]

```
BG_MODEL = FANMOD
MOTIF_ID = 36_001001002
TF_TYPE = ath
UPSTREAM = -2000_0


PVals
FN_0.2	FN_0.4	FN_0.6	FN_0.8
dg_60.genes	0.844	0.628	1	0
dg_70.genes	0.85	0.624	1	0
dg_80.genes	0.858	0.642	1	0

ZScores
FN_0.2	FN_0.4	FN_0.6	FN_0.8
dg_60.genes	-1.048	-0.362	-5.924	5.898
dg_70.genes	-1.064	-0.341	-5.705	5.893
dg_80.genes	-1.065	-0.373	-5.792	5.857

StDevs
FN_0.2	FN_0.4	FN_0.6	FN_0.8
dg_60.genes	9413.062	16466.568	4979.396	968.906
dg_70.genes	9214.13	16615.295	5200.562	966.698
dg_80.genes	9270.881	16421.306	5075.099	972.052
```
