# Supplementary material for: Sustained-input switches for transcription factors and microRNAs are central building blocks of eukaryotic gene circuits
Source: Genome Biol. 2013 Aug 23;14(8):R85. doi: 10.1186/gb-2013-14-8-r85 (PMC4054853; doi:10.1186/gb-2013-14-8-r85)
Supplement: Additional file 5 — HTML Browsable Motif Output. Zipped folder containing all WaRSwap and FANMOD motif output, viewable in a web browser. [file gb-2013-14-8-r85-S5.ZIP › HTML_browsable_motif_output/FANMOD_ath_tair9/sigs_fanmodm-2000.pvals.heatmaps.html/motif_id_38_000100110_tftype_ath_upstream_-1000_0.html]

```
BG_MODEL = FANMOD
MOTIF_ID = 38_000100110
TF_TYPE = ath
UPSTREAM = -1000_0


PVals
FN_0.2	FN_0.4	FN_0.6	FN_0.8
dg_60.genes	0.976	0.105	0.006	0.8
dg_70.genes	0.984	0.095	0.009	0.796
dg_80.genes	0.972	0.109	0.007	0.779

ZScores
FN_0.2	FN_0.4	FN_0.6	FN_0.8
dg_60.genes	-2.057	1.251	2.722	-1.056
dg_70.genes	-2.114	1.293	2.703	-1.041
dg_80.genes	-1.996	1.264	2.649	-0.998

StDevs
FN_0.2	FN_0.4	FN_0.6	FN_0.8
dg_60.genes	60.937	45.215	14.287	2.597
dg_70.genes	59.443	44.683	14.398	2.571
dg_80.genes	61.602	44.61	14.472	2.54
```
