# Supplementary material for: Sustained-input switches for transcription factors and microRNAs are central building blocks of eukaryotic gene circuits
Source: Genome Biol. 2013 Aug 23;14(8):R85. doi: 10.1186/gb-2013-14-8-r85 (PMC4054853; doi:10.1186/gb-2013-14-8-r85)
Supplement: Additional file 5 — HTML Browsable Motif Output. Zipped folder containing all WaRSwap and FANMOD motif output, viewable in a web browser. [file gb-2013-14-8-r85-S5.ZIP › HTML_browsable_motif_output/FANMOD_ath_tair9/sigs_fanmodm-2000.pvals.heatmaps.html/motif_id_38_000101101_tftype_ath_upstream_-1000_0.html]

```
BG_MODEL = FANMOD
MOTIF_ID = 38_000101101
TF_TYPE = ath
UPSTREAM = -1000_0


PVals
FN_0.2	FN_0.4	FN_0.6	FN_0.8
dg_60.genes	0.003	0.002	0.384	0.172
dg_70.genes	0.017	0.002	0.796	0.154
dg_80.genes	0.013	0	0.69	0.043

ZScores
FN_0.2	FN_0.4	FN_0.6	FN_0.8
dg_60.genes	2.632	2.746	-0.138	-0.425
dg_70.genes	2.054	3.08	-1.222	-0.4
dg_80.genes	2.144	3.32	-1.098	-0.21

StDevs
FN_0.2	FN_0.4	FN_0.6	FN_0.8
dg_60.genes	11.79	7.279	1.519	0.511
dg_70.genes	10.401	6.88	1.418	0.452
dg_80.genes	9.193	6.226	1.022	0.214
```
