# Supplementary material for: Sustained-input switches for transcription factors and microRNAs are central building blocks of eukaryotic gene circuits
Source: Genome Biol. 2013 Aug 23;14(8):R85. doi: 10.1186/gb-2013-14-8-r85 (PMC4054853; doi:10.1186/gb-2013-14-8-r85)
Supplement: Additional file 5 — HTML Browsable Motif Output. Zipped folder containing all WaRSwap and FANMOD motif output, viewable in a web browser. [file gb-2013-14-8-r85-S5.ZIP › HTML_browsable_motif_output/FANMOD_ath_tair9/sigs_fanmodm-2000.pvals.heatmaps.html/motif_id_38_000101101_tftype_ath_upstream_-2000_0.html]

```
BG_MODEL = FANMOD
MOTIF_ID = 38_000101101
TF_TYPE = ath
UPSTREAM = -2000_0


PVals
FN_0.2	FN_0.4	FN_0.6	FN_0.8
dg_60.genes	0.004	0.006	0.002	0.002
dg_70.genes	0.017	0.004	0.001	0
dg_80.genes	0.006	0.001	0	0

ZScores
FN_0.2	FN_0.4	FN_0.6	FN_0.8
dg_60.genes	2.502	2.507	3.116	3.084
dg_70.genes	1.937	2.645	3.275	4.22
dg_80.genes	2.365	3.258	4.13	3.465

StDevs
FN_0.2	FN_0.4	FN_0.6	FN_0.8
dg_60.genes	14.364	8.02	5.484	3.294
dg_70.genes	13.507	7.604	5.02	2.571
dg_80.genes	10.68	6.933	4.627	2.717
```
