# Supplementary material for: Sustained-input switches for transcription factors and microRNAs are central building blocks of eukaryotic gene circuits
Source: Genome Biol. 2013 Aug 23;14(8):R85. doi: 10.1186/gb-2013-14-8-r85 (PMC4054853; doi:10.1186/gb-2013-14-8-r85)
Supplement: Additional file 5 — HTML Browsable Motif Output. Zipped folder containing all WaRSwap and FANMOD motif output, viewable in a web browser. [file gb-2013-14-8-r85-S5.ZIP › HTML_browsable_motif_output/FANMOD_ath_tair9/sigs_fanmodm-2000.pvals.heatmaps.html/motif_id_38_000101101_tftype_ath_upstream_-3000_0.html]

```
BG_MODEL = FANMOD
MOTIF_ID = 38_000101101
TF_TYPE = ath
UPSTREAM = -3000_0


PVals
FN_0.2	FN_0.4	FN_0.6	FN_0.8
dg_60.genes	0.006	0.012	0.073	0.016
dg_70.genes	0.029	0.013	0.038	0.001
dg_80.genes	0.019	0.004	0.037	0.001

ZScores
FN_0.2	FN_0.4	FN_0.6	FN_0.8
dg_60.genes	2.378	2.186	1.436	2.176
dg_70.genes	1.909	2.118	1.686	3.137
dg_80.genes	2.066	2.477	1.759	2.563

StDevs
FN_0.2	FN_0.4	FN_0.6	FN_0.8
dg_60.genes	14.449	9.635	7.226	3.645
dg_70.genes	13.48	9.188	6.864	2.804
dg_80.genes	10.834	8.113	6.298	2.919
```
