# Supplementary material for: Sustained-input switches for transcription factors and microRNAs are central building blocks of eukaryotic gene circuits
Source: Genome Biol. 2013 Aug 23;14(8):R85. doi: 10.1186/gb-2013-14-8-r85 (PMC4054853; doi:10.1186/gb-2013-14-8-r85)
Supplement: Additional file 5 — HTML Browsable Motif Output. Zipped folder containing all WaRSwap and FANMOD motif output, viewable in a web browser. [file gb-2013-14-8-r85-S5.ZIP › HTML_browsable_motif_output/FANMOD_ath_tair9/sigs_fanmodm-2000.pvals.heatmaps.html/motif_id_38_001101001_tftype_ath_upstream_-1000_0.html]

```
BG_MODEL = FANMOD
MOTIF_ID = 38_001101001
TF_TYPE = ath
UPSTREAM = -1000_0


PVals
FN_0.2	FN_0.4	FN_0.6	FN_0.8
dg_60.genes	0.932	0.503	0	1
dg_70.genes	0.944	0.509	0	1
dg_80.genes	0.937	0.511	0.001	1

ZScores
FN_0.2	FN_0.4	FN_0.6	FN_0.8
dg_60.genes	-1.521	-0.011	3.424	-3.279
dg_70.genes	-1.529	-0.023	3.356	-3.299
dg_80.genes	-1.553	-0.041	3.282	-3.185

StDevs
FN_0.2	FN_0.4	FN_0.6	FN_0.8
dg_60.genes	269.123	199.385	39.715	10.377
dg_70.genes	267.287	196.266	40.146	10.449
dg_80.genes	264.163	195.814	40.594	10.701
```
