# Supplementary material for: Sustained-input switches for transcription factors and microRNAs are central building blocks of eukaryotic gene circuits
Source: Genome Biol. 2013 Aug 23;14(8):R85. doi: 10.1186/gb-2013-14-8-r85 (PMC4054853; doi:10.1186/gb-2013-14-8-r85)
Supplement: Additional file 6 — PWM Log-likelihood Score Distributions, Arabidopsis Only Set. Zipped pdf file containing plots of foreground and background log-likelihood scoring distributions for each PWM in the Arabidopsis Only set. [file gb-2013-14-8-r85-S6.ZIP › ath_pwms_hists.pdf]

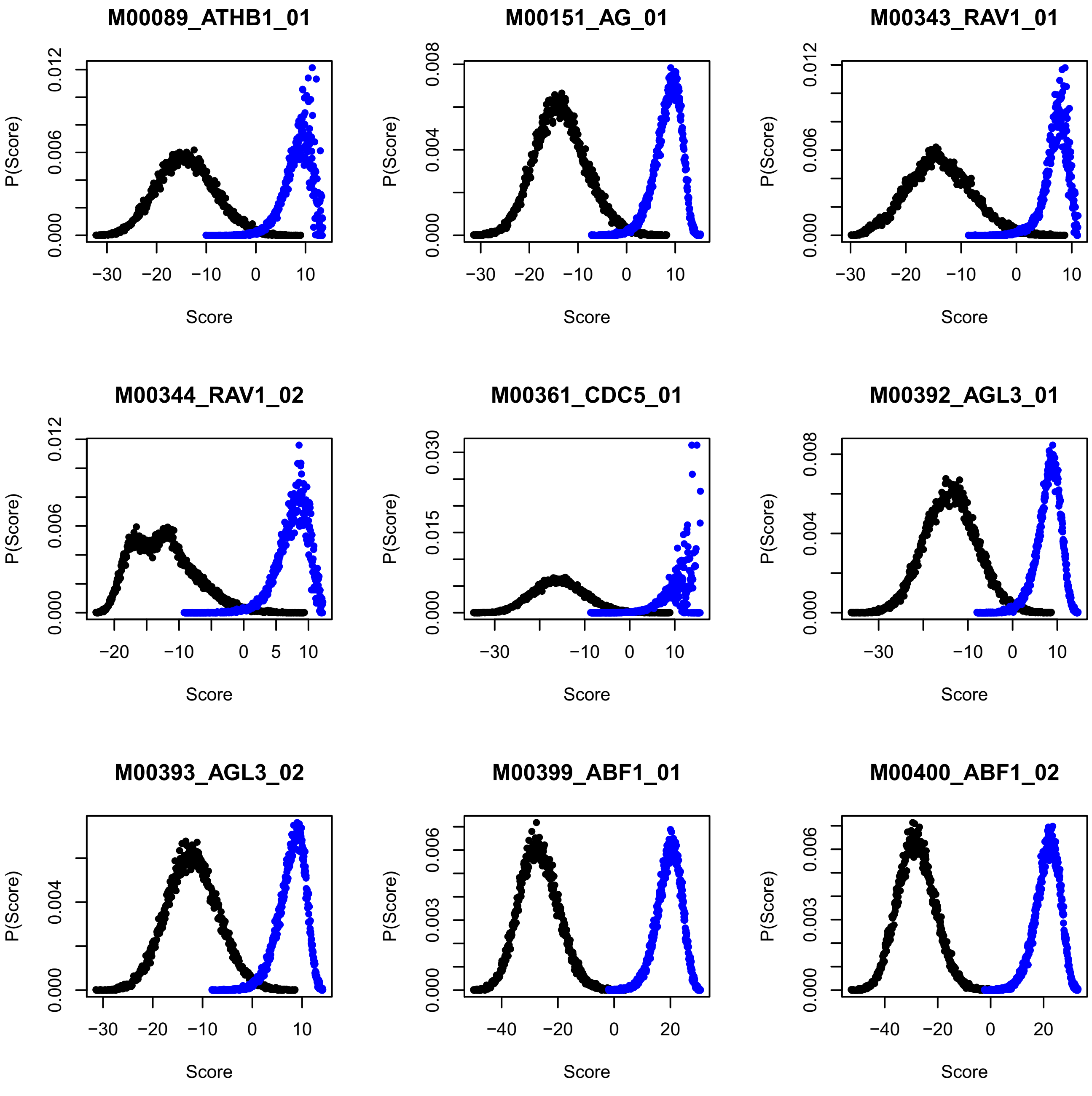
M00089_ATHB1_01
M00151_AG_01
M00343_RAV1_01
M00344_RAV1_02
M00361_CDC5_01
M00392_AGL3_01
M00393_AGL3_02
M00399_ABF1_01
M00400_ABF1_02
P(Score)
Score

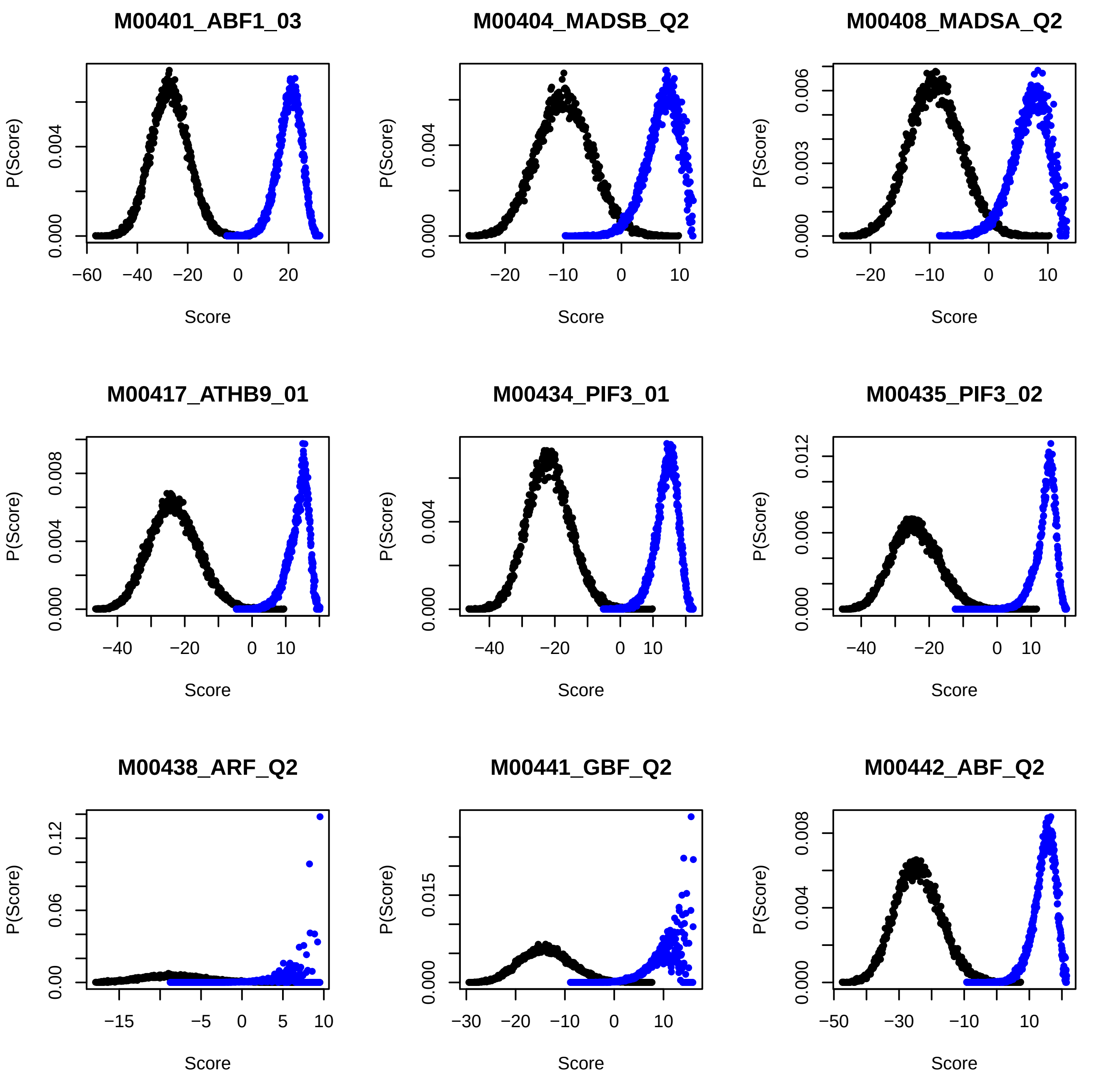

M00401_ABF1_03
P(Score)
0.000
0.004
−60
−40
−20
0
20
Score
M00404_MADSB_Q2
P(Score)
0.000
0.004
−20
−10
0
10
Score
M00408_MADSA_Q2
P(Score)
0.000
0.003
0.006
−20
−10
0
10
Score
M00417_ATHB9_01
P(Score)
0.000
0.004
0.008
−40
−20
0
10
Score
M00434_PIF3_01
P(Score)
0.000
0.004
−40
−20
0
10
Score
M00435_PIF3_02
P(Score)
0.000
0.006
0.012
−40
−20
0
10
Score
M00438_ARF_Q2
P(Score)
0.00
0.06
0.12
−15
−5
0
5
10
Score
M00441_GBF_Q2
P(Score)
0.000
0.015
−30
−20
−10
0
10
Score
M00442_ABF_Q2
P(Score)
0.000
0.004
0.008
−50
−30
−10
10
Score

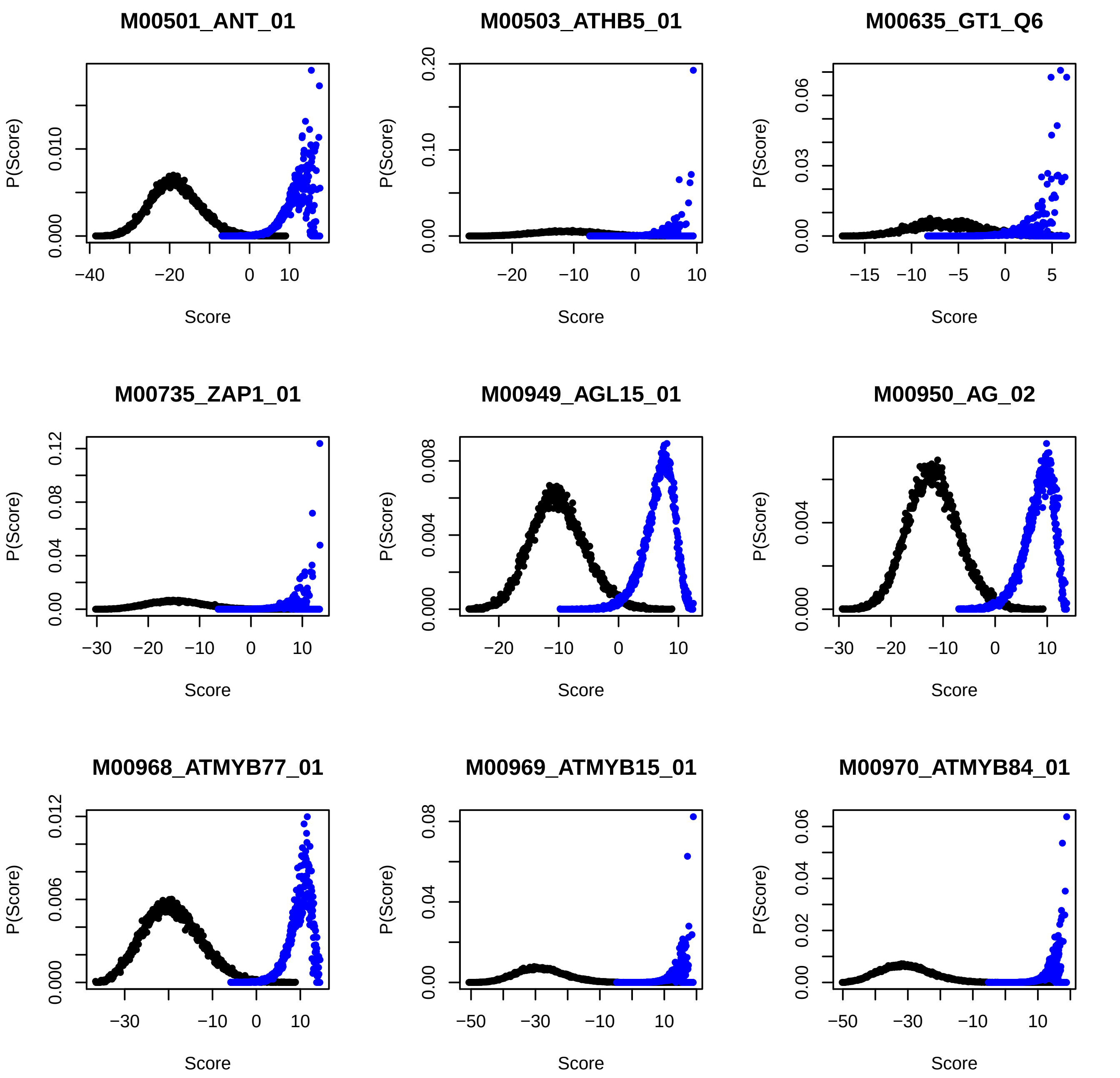
M00501_ANT_01
P(Score)
0.000
0.010
−40
−20
0
10
Score
M00503_ATHB5_01
P(Score)
0.00
0.10
0.20
−20
−10
0
10
Score
M00635_GT1_Q6
P(Score)
0.00
0.03
0.06
−15
−10
−5
0
5
Score
M00735_ZAP1_01
P(Score)
0.00
0.04
0.08
0.12
−30
−20
−10
0
10
Score
M00949_AGL15_01
P(Score)
0.000
0.004
0.008
−20
−10
0
10
Score
M00950_AG_02
P(Score)
0.000
0.004
−30
−20
−10
0
10
Score
M00968_ATMYB77_01
P(Score)
0.000
0.006
0.012
−30
−10
0
10
Score
M00969_ATMYB15_01
P(Score)
0.00
0.04
0.08
−50
−30
−10
10
Score
M00970_ATMYB84_01
P(Score)
0.00
0.02
0.04
0.06
−50
−30
−10
10
Score

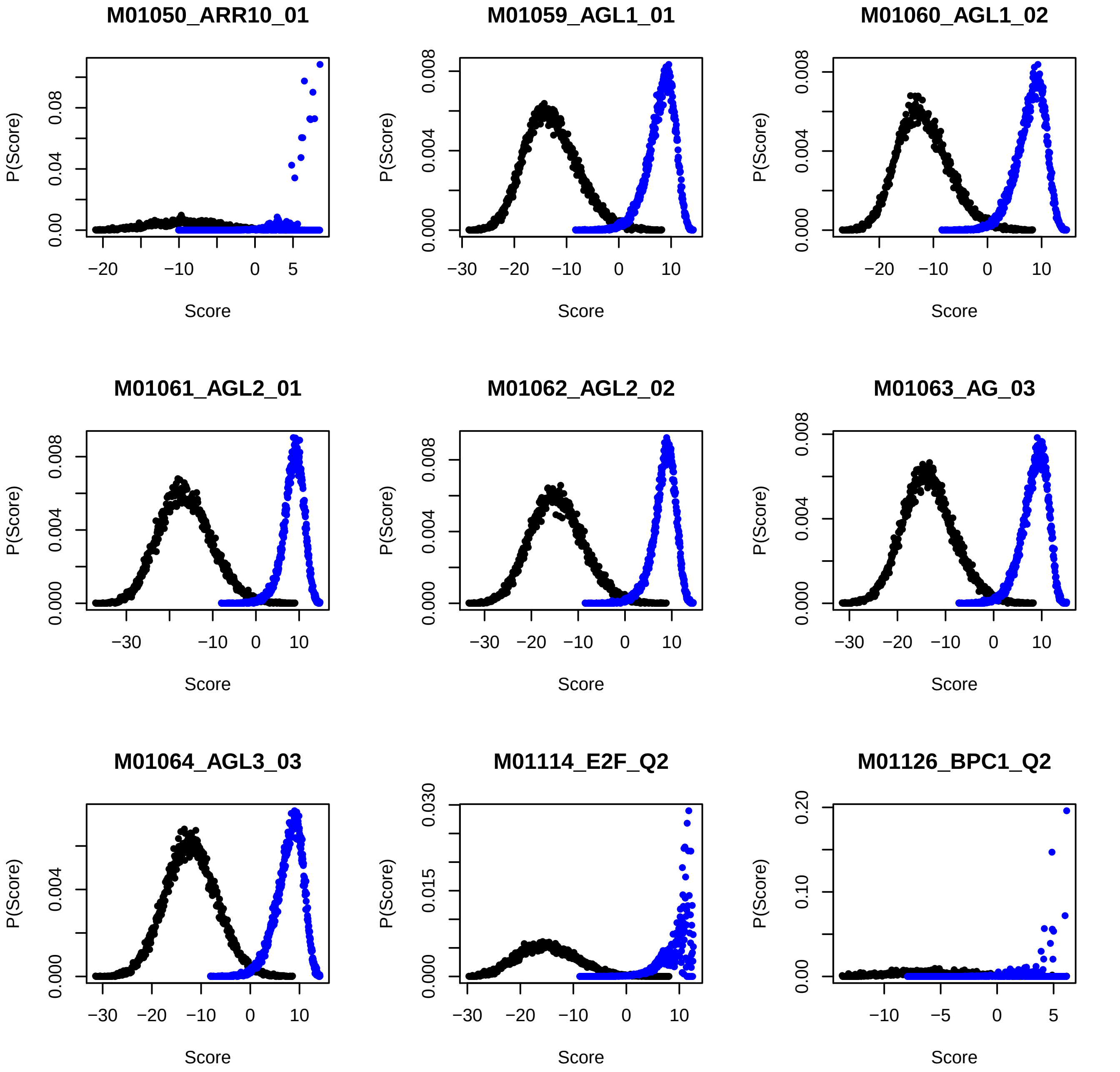
M01050_ARR10_01
P(Score)
0.00
0.04
0.08
−20
−10
0
5
Score
M01059_AGL1_01
P(Score)
0.000
0.004
0.008
−30
−20
−10
0
10
Score
M01060_AGL1_02
P(Score)
0.000
0.004
0.008
−20
−10
0
10
Score
M01061_AGL2_01
P(Score)
0.000
0.004
0.008
−30
−10
0
10
Score
M01062_AGL2_02
P(Score)
0.000
0.004
0.008
−30
−20
−10
0
10
Score
M01063_AG_03
P(Score)
0.000
0.004
0.008
−30
−20
−10
0
10
Score
M01064_AGL3_03
P(Score)
0.000
0.004
−30
−20
−10
0
10
Score
M01114_E2F_Q2
P(Score)
0.000
0.015
0.030
−30
−20
−10
0
10
Score
M01126_BPC1_Q2
P(Score)
0.00
0.10
0.20
−10
−5
0
5
Score

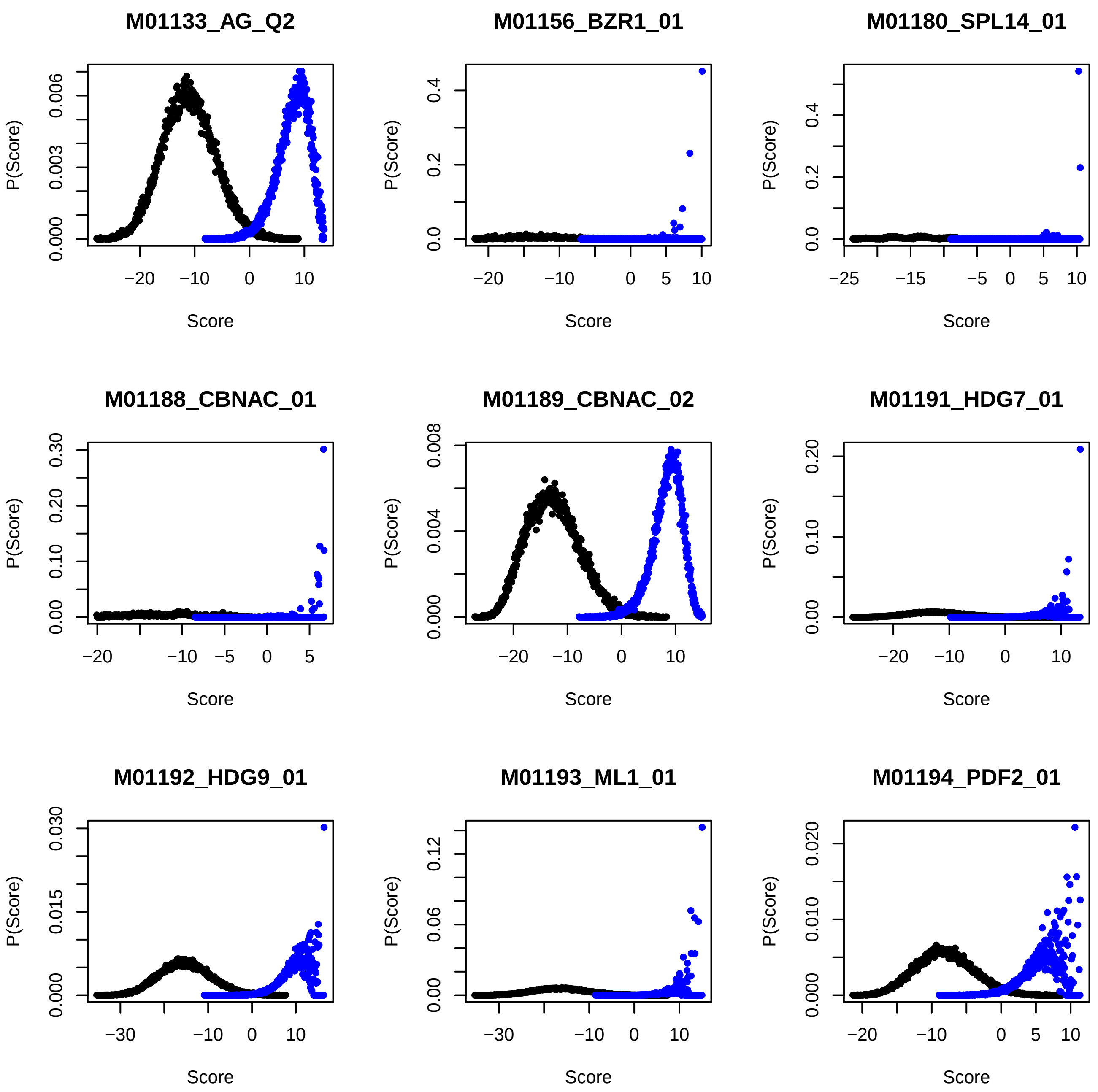
M01133_AG_Q2
M01156_BZR1_01
M01180_SPL14_01
M01188_CBNAC_01
M01189_CBNAC_02
M01191_HDG7_01
M01192_HDG9_01
M01193_ML1_01
M01194_PDF2_01
P(Score)
Score
