# Supplementary material for: Sustained-input switches for transcription factors and microRNAs are central building blocks of eukaryotic gene circuits
Source: Genome Biol. 2013 Aug 23;14(8):R85. doi: 10.1186/gb-2013-14-8-r85 (PMC4054853; doi:10.1186/gb-2013-14-8-r85)
Supplement: Additional file 7 — PWM Log-likelihood Score Distributions, Comprehensive Set. Zipped pdf file containing plots of foreground and background log-likelihood scoring distributions for each PWM in the Comprehensive set. [file gb-2013-14-8-r85-S7.ZIP › all_pwms_hists.pdf]

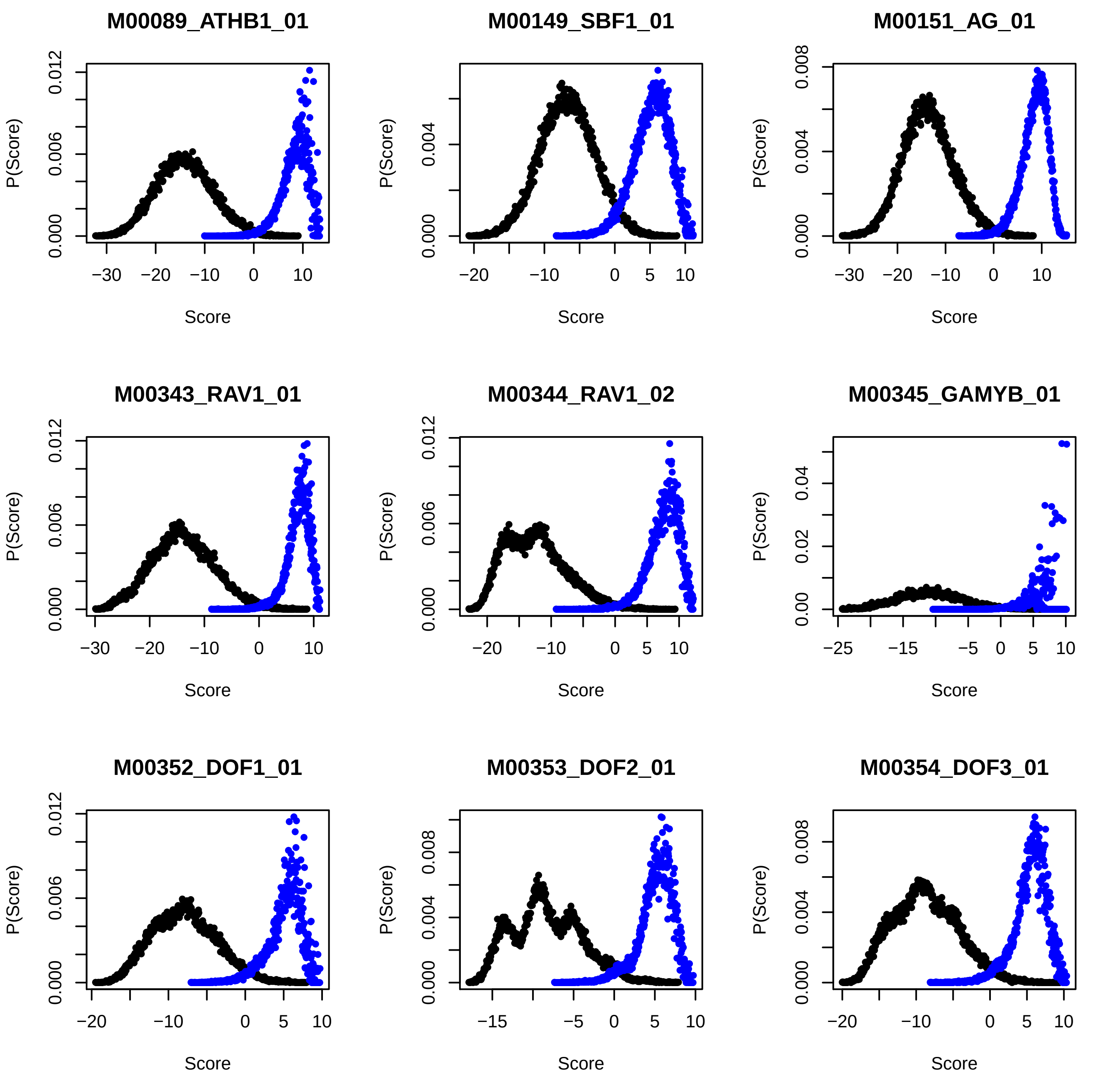
M00089_ATHB1_01
M00149_SBF1_01
M00151_AG_01
M00343_RAV1_01
M00344_RAV1_02
M00345_GAMYB_01
M00352_DOF1_01
M00353_DOF2_01
M00354_DOF3_01
P(Score)
Score

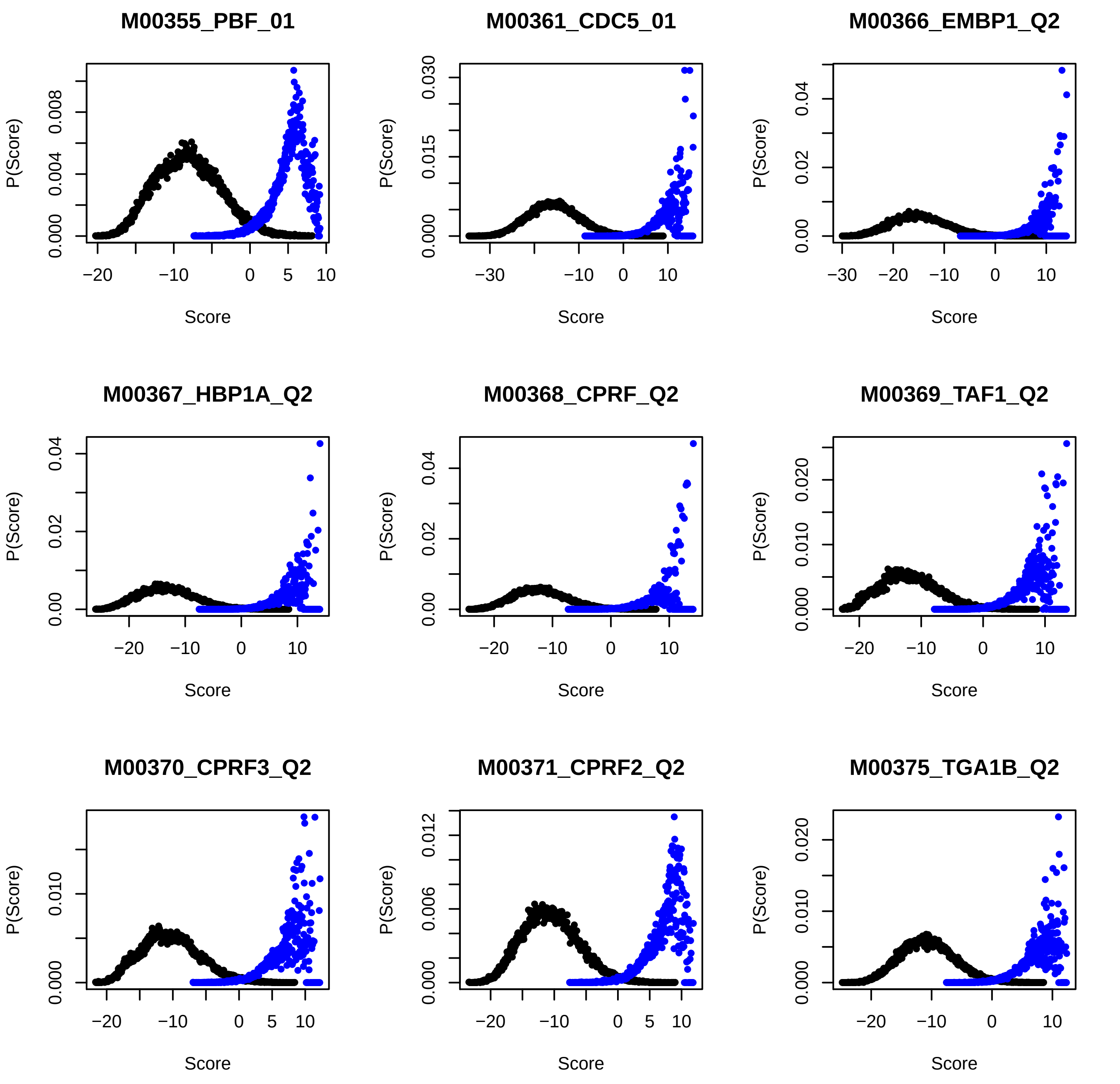
M00355_PBF_01
M00361_CDC5_01
M00366_EMBP1_Q2
M00367_HBP1A_Q2
M00368_CPRF_Q2
M00369_TAF1_Q2
M00370_CPRF3_Q2
M00371_CPRF2_Q2
M00375_TGA1B_Q2
P(Score)
Score

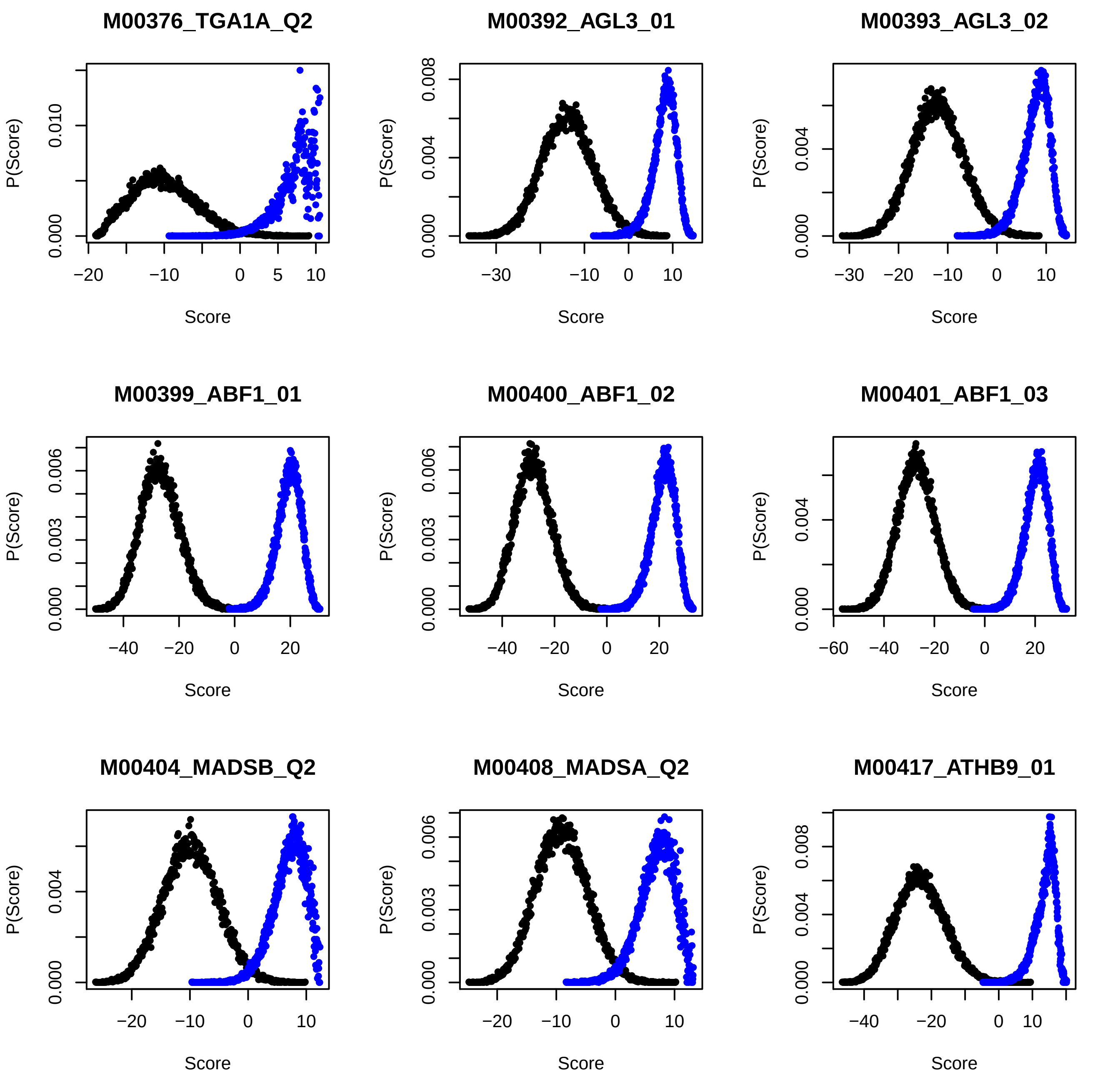
M00376_TGA1A_Q2
P(Score)
0.000
0.010
−20
−10
0
5
10
Score
M00392_AGL3_01
P(Score)
0.000
0.004
0.008
−30
−10
0
10
Score
M00393_AGL3_02
P(Score)
0.000
0.004
−30
−20
−10
0
10
Score
M00399_ABF1_01
P(Score)
0.000
0.003
0.006
−40
−20
0
20
Score
M00400_ABF1_02
P(Score)
0.000
0.003
0.006
−40
−20
0
20
Score
M00401_ABF1_03
P(Score)
0.000
0.004
−60
−40
−20
0
20
Score
M00404_MADSB_Q2
P(Score)
0.000
0.004
−20
−10
0
10
Score
M00408_MADSA_Q2
P(Score)
0.000
0.003
0.006
−20
−10
0
10
Score
M00417_ATHB9_01
P(Score)
0.000
0.004
0.008
−40
−20
0
10
Score

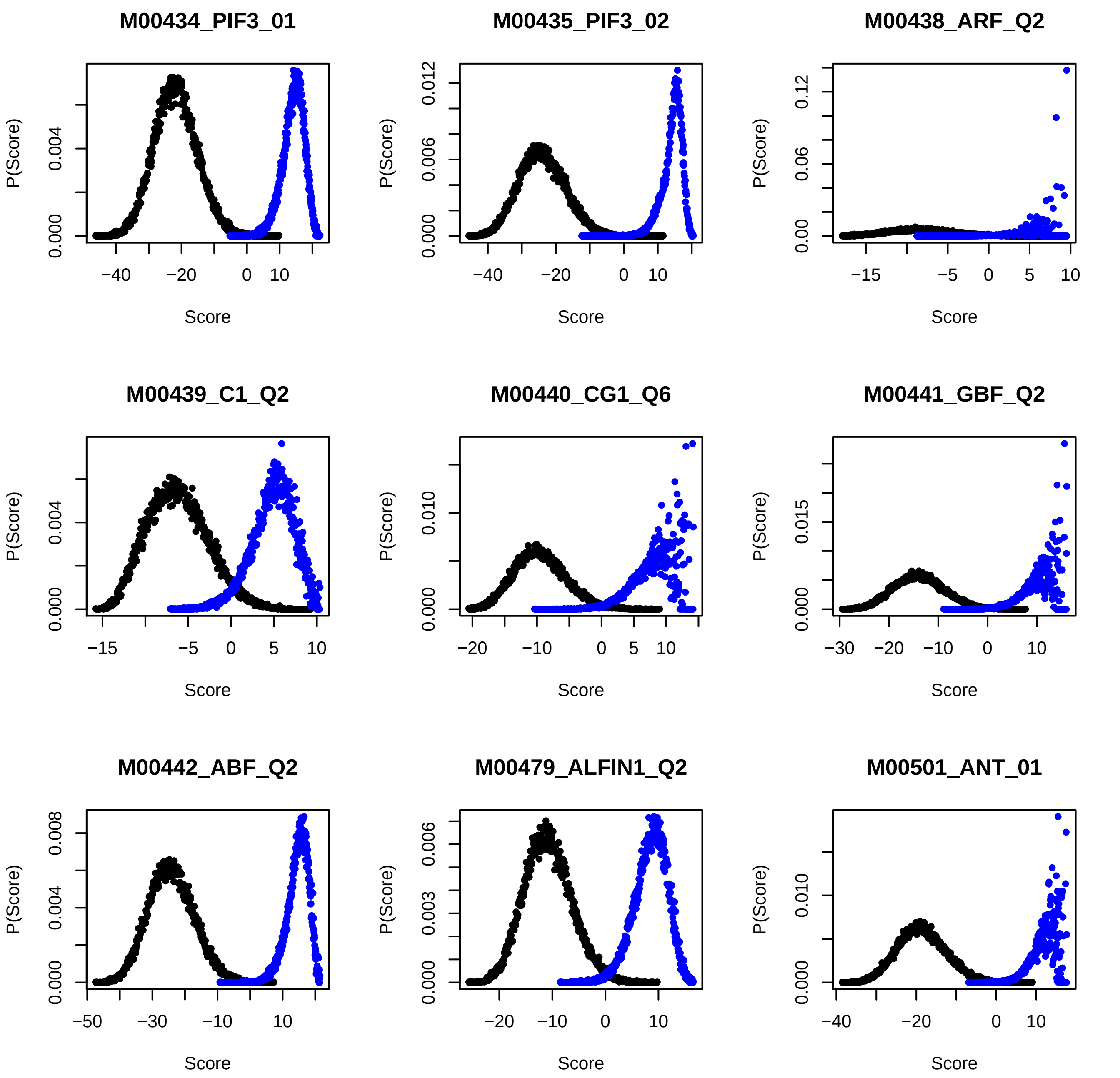

M00434_PIF3_01
P(Score)
0.000
0.004
−40
−20
0
10
Score
M00435_PIF3_02
P(Score)
0.000
0.006
0.012
−40
−20
0
10
Score
M00438_ARF_Q2
P(Score)
0.00
0.06
0.12
−15
−5
0
5
10
Score
M00439_C1_Q2
P(Score)
0.000
0.004
−15
−5
0
5
10
Score
M00440_CG1_Q6
P(Score)
0.000
0.010
−20
−10
0
5
10
Score
M00441_GBF_Q2
P(Score)
0.000
0.015
−30
−20
−10
0
10
Score
M00442_ABF_Q2
P(Score)
0.000
0.004
0.008
−50
−30
−10
10
Score
M00479_ALFIN1_Q2
P(Score)
0.000
0.003
0.006
−20
−10
0
10
Score
M00501_ANT_01
P(Score)
0.000
0.010
−40
−20
0
10
Score

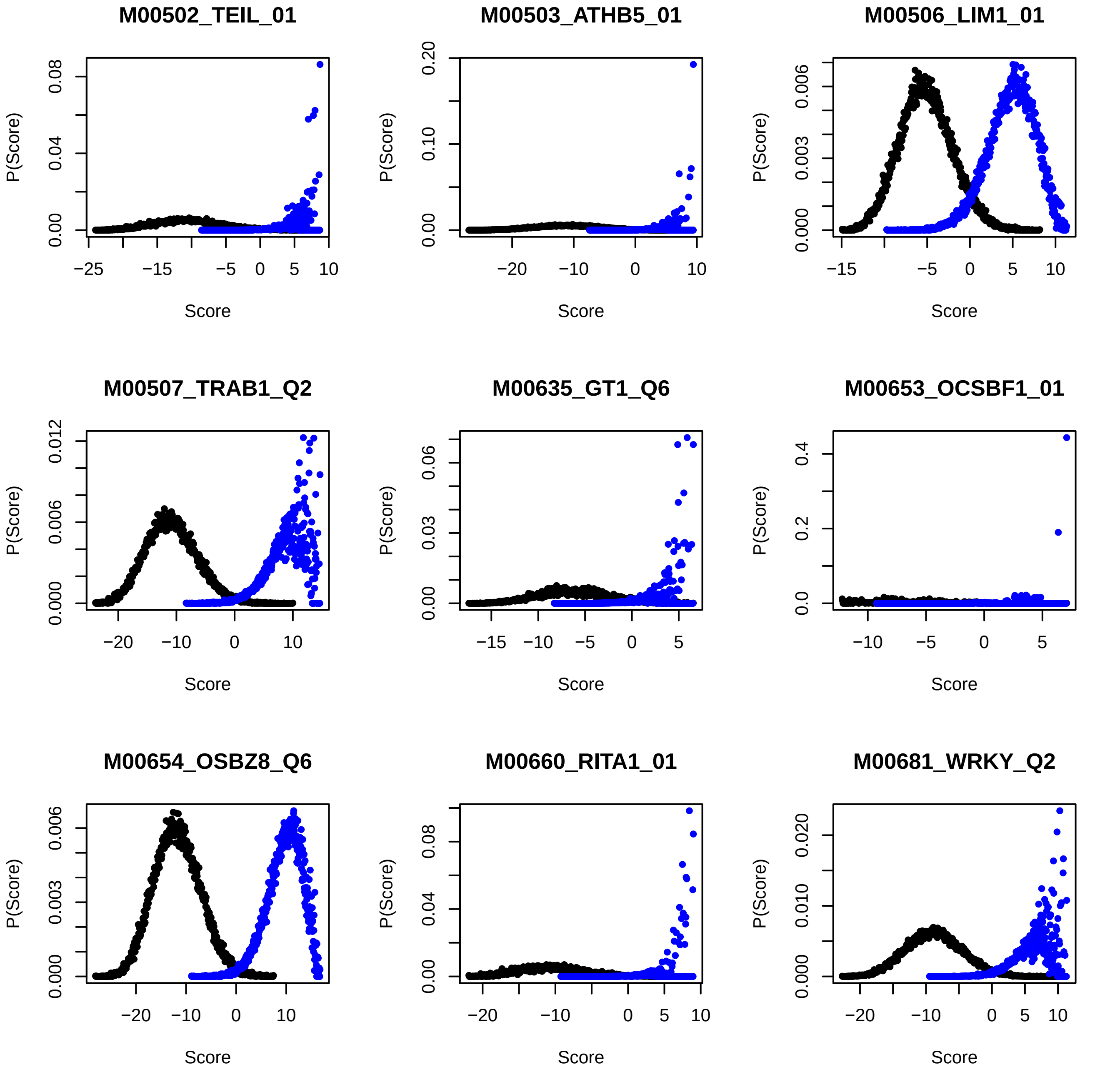
M00502_TEIL_01
M00503_ATHB5_01
M00506_LIM1_01
M00507_TRAB1_Q2
M00635_GT1_Q6
M00653_OCSBF1_01
M00654_OSBZ8_Q6
M00660_RITA1_01
M00681_WRKY_Q2
P(Score)
Score

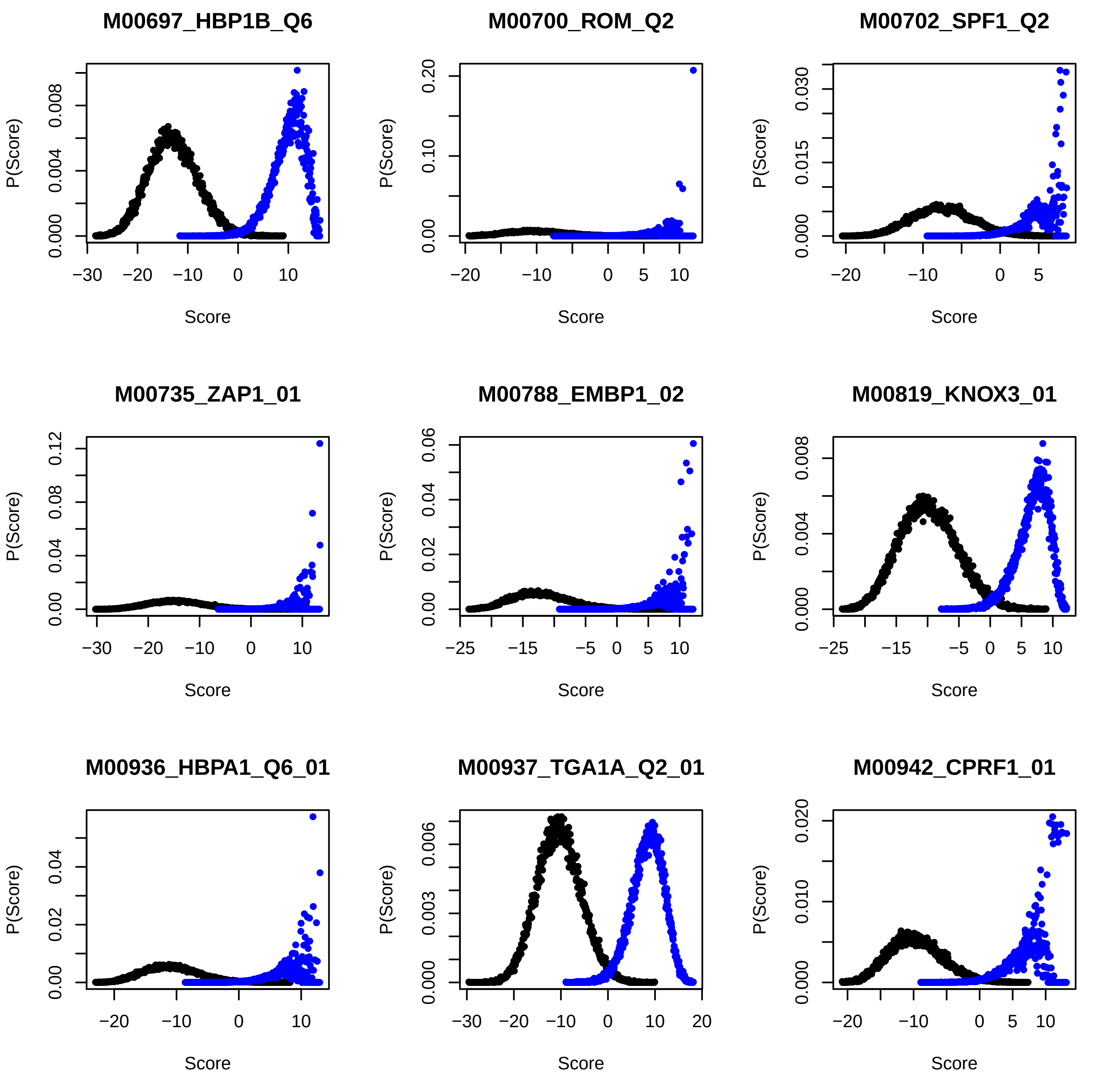
M00697_HBP1B_Q6
P(Score)
0.000
0.004
0.008
−30
−20
−10
0
10
Score
M00700_ROM_Q2
P(Score)
0.00
0.10
0.20
−20
−10
0
5
10
Score
M00702_SPF1_Q2
P(Score)
0.000
0.015
0.030
−20
−10
0
5
Score
M00735_ZAP1_01
P(Score)
0.00
0.04
0.08
0.12
−30
−20
−10
0
10
Score
M00788_EMBP1_02
P(Score)
0.00
0.02
0.04
0.06
−25
−15
−5
0
5
10
Score
M00819_KNOX3_01
P(Score)
0.000
0.004
0.008
−25
−15
−5
0
5
10
Score
M00936_HBPA1_Q6_01
P(Score)
0.00
0.02
0.04
−20
−10
0
10
Score
M00937_TGA1A_Q2_01
P(Score)
0.000
0.003
0.006
−30
−20
−10
0
10
20
Score
M00942_CPRF1_01
P(Score)
0.000
0.010
0.020
−20
−10
0
5
10
Score

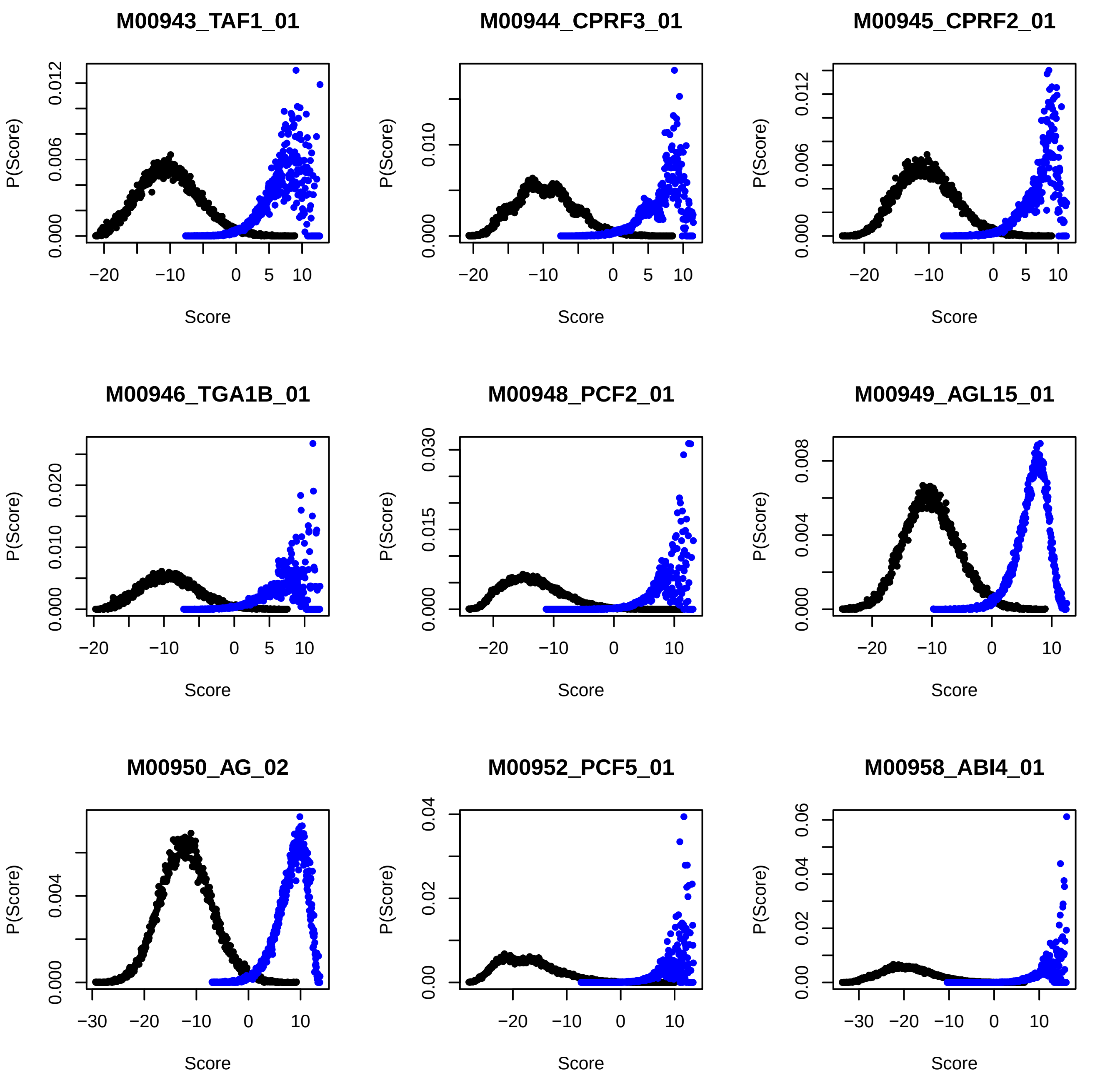
M00943_TAF1_01
M00944_CPRF3_01
M00945_CPRF2_01
M00946_TGA1B_01
M00948_PCF2_01
M00949_AGL15_01
M00950_AG_02
M00952_PCF5_01
M00958_ABI4_01
P(Score)
Score

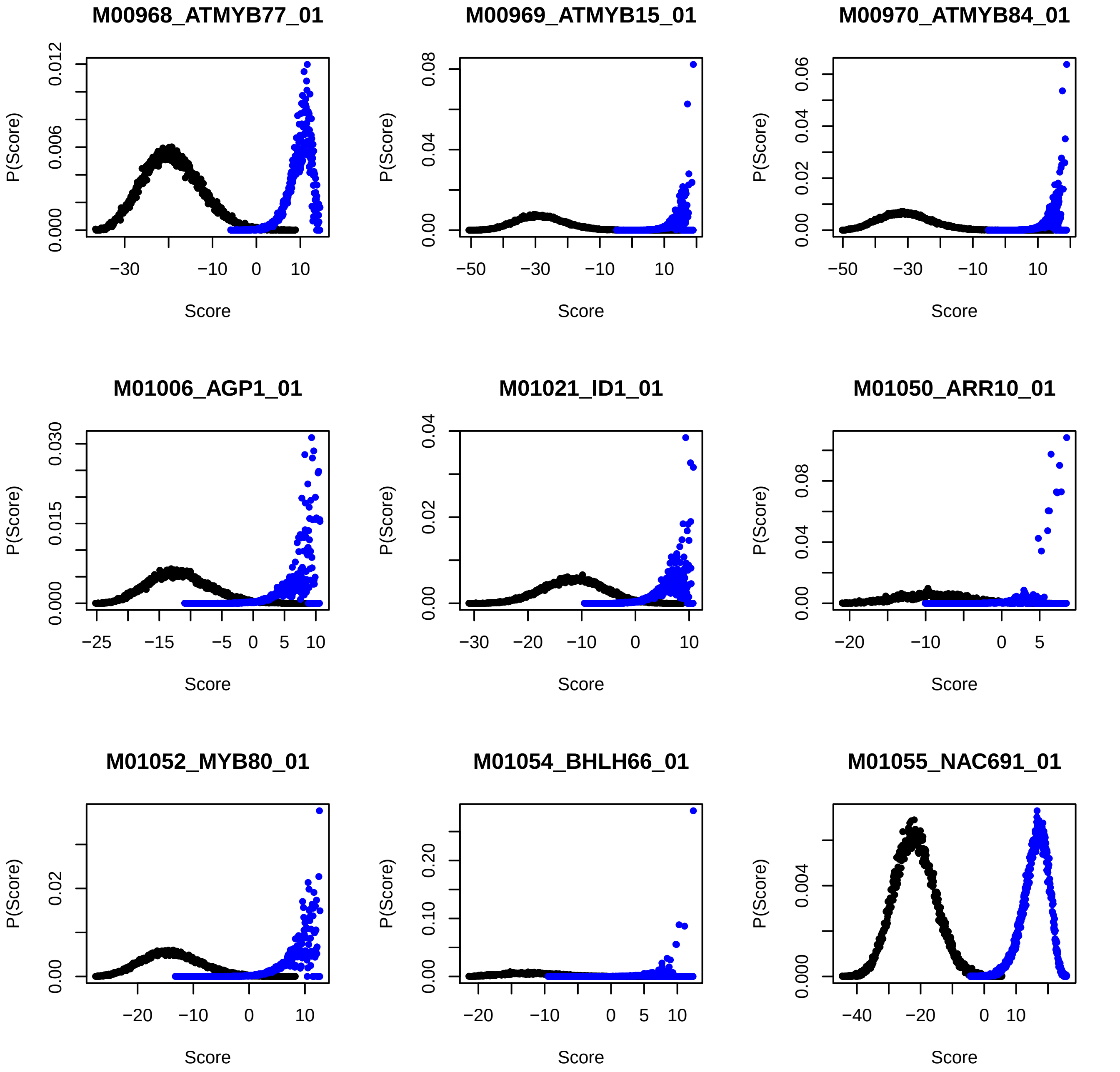

M00968_ATMYB77_01
P(Score)
0.000
0.006
0.012
−30
−10
0
10
Score
M00969_ATMYB15_01
P(Score)
0.00
0.04
0.08
−50
−30
−10
10
Score
M00970_ATMYB84_01
P(Score)
0.00
0.02
0.04
0.06
−50
−30
−10
10
Score
M01006_AGP1_01
P(Score)
0.000
0.015
0.030
−25
−15
−5
0
5
10
Score
M01021_ID1_01
P(Score)
0.00
0.02
0.04
−30
−20
−10
0
10
Score
M01050_ARR10_01
P(Score)
0.00
0.04
0.08
−20
−10
0
5
Score
M01052_MYB80_01
P(Score)
0.00
0.02
−20
−10
0
10
Score
M01054_BHLH66_01
P(Score)
0.00
0.10
0.20
−20
−10
0
5
10
Score
M01055_NAC691_01
P(Score)
0.000
0.004
−40
−20
0
10
Score

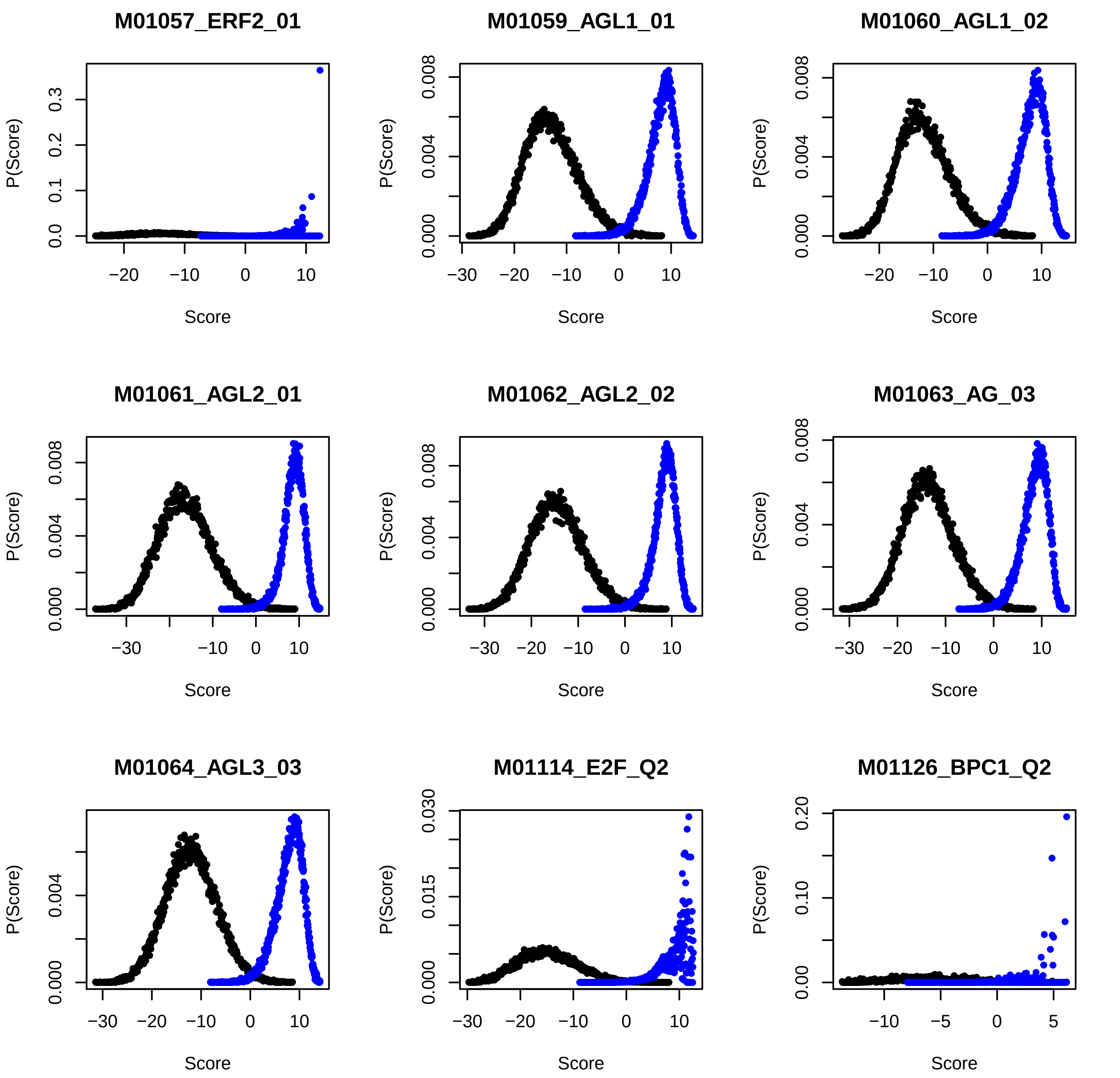
M01057_ERF2_01
M01059_AGL1_01
M01060_AGL1_02
M01061_AGL2_01
M01062_AGL2_02
M01063_AG_03
M01064_AGL3_03
M01114_E2F_Q2
M01126_BPC1_Q2
P(Score)
Score

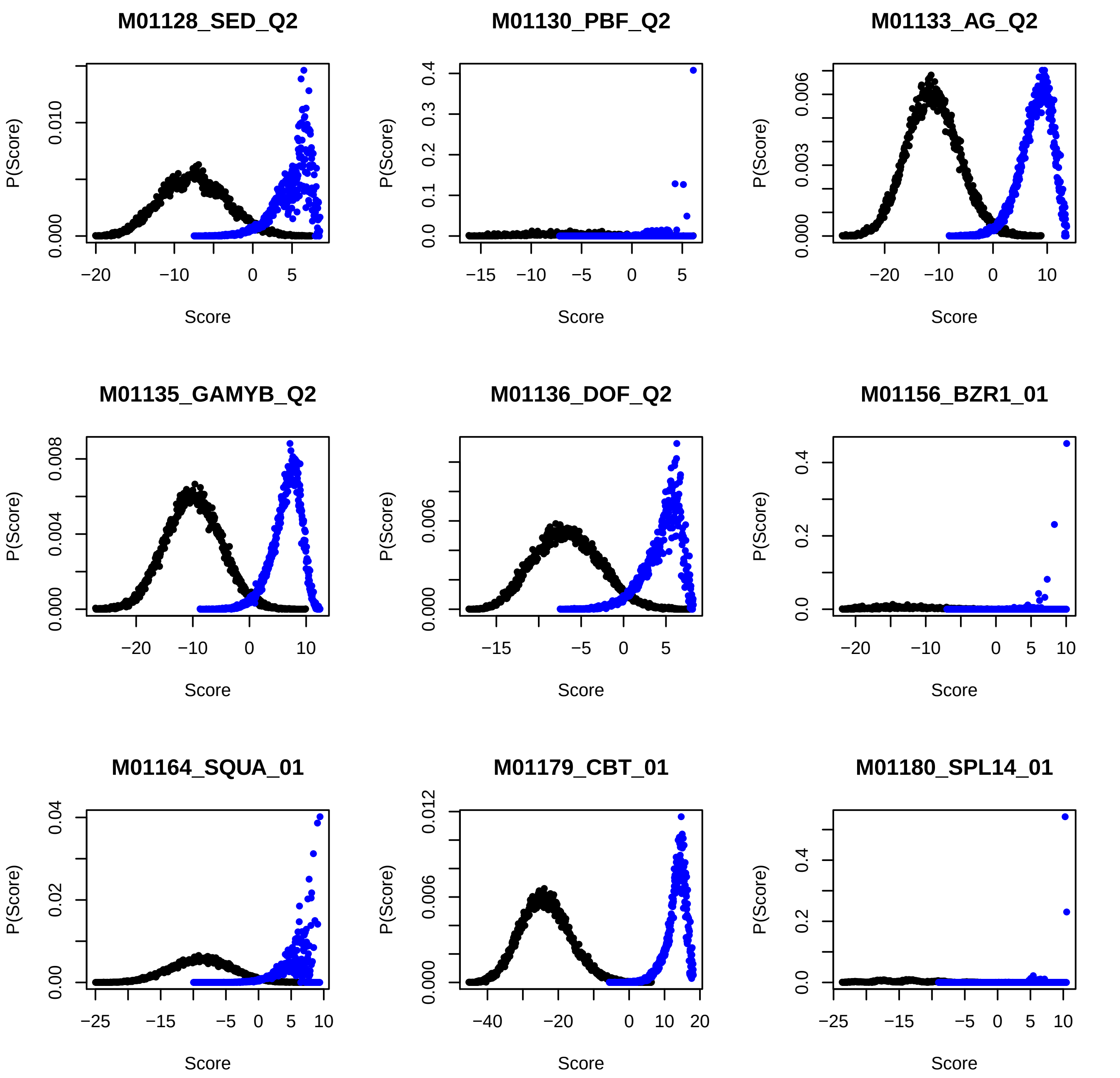
M01128_SED_Q2
P(Score)
0.000
0.010
−20
−10
0
5
Score
M01130_PBF_Q2
P(Score)
0.0
0.1
0.2
0.3
0.4
−15
−10
−5
0
5
Score
M01133_AG_Q2
P(Score)
0.000
0.003
0.006
−20
−10
0
10
Score
M01135_GAMYB_Q2
P(Score)
0.000
0.004
0.008
−20
−10
0
10
Score
M01136_DOF_Q2
P(Score)
0.000
0.006
−15
−5
0
5
Score
M01156_BZR1_01
P(Score)
0.0
0.2
0.4
−20
−10
0
5
10
Score
M01164_SQUA_01
P(Score)
0.00
0.02
0.04
−25
−15
−5
0
5
10
Score
M01179_CBT_01
P(Score)
0.000
0.006
0.012
−40
−20
0
10
20
Score
M01180_SPL14_01
P(Score)
0.0
0.2
0.4
−25
−15
−5
0
5
10
Score

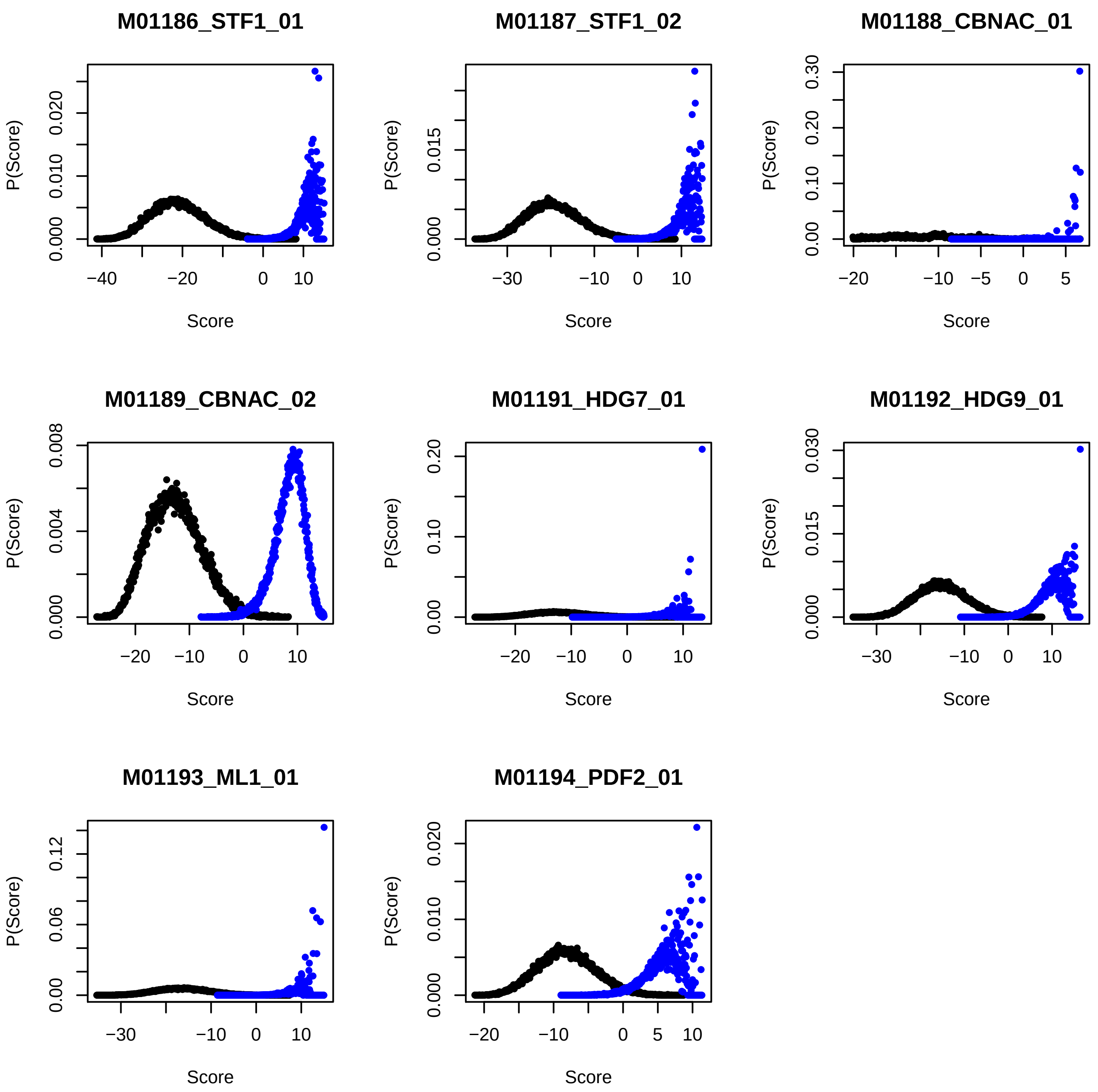
M01186_STF1_01
P(Score)
0.000
0.010
0.020
−40
−20
0
10
Score
M01187_STF1_02
P(Score)
0.000
0.015
−30
−10
0
10
Score
M01188_CBNAC_01
P(Score)
0.00
0.10
0.20
0.30
−20
−10
−5
0
5
Score
M01189_CBNAC_02
P(Score)
0.000
0.004
0.008
−20
−10
0
10
Score
M01191_HDG7_01
P(Score)
0.00
0.10
0.20
−20
−10
0
10
Score
M01192_HDG9_01
P(Score)
0.000
0.015
0.030
−30
−10
0
10
Score
M01193_ML1_01
P(Score)
0.00
0.06
0.12
−30
−10
0
10
Score
M01194_PDF2_01
P(Score)
0.000
0.010
0.020
−20
−10
0
5
10
Score
