# Supplementary material for: Sustained-input switches for transcription factors and microRNAs are central building blocks of eukaryotic gene circuits
Source: Genome Biol. 2013 Aug 23;14(8):R85. doi: 10.1186/gb-2013-14-8-r85 (PMC4054853; doi:10.1186/gb-2013-14-8-r85)
Supplement: Additional file 8 — PWM ROC Curves, Arabidopsis Only Set. Zipped pdf file containing ROC curve plots for each PWM in the Arabidopsis Only set. [file gb-2013-14-8-r85-S8.ZIP › ath_pwms_roc_curves.pdf]

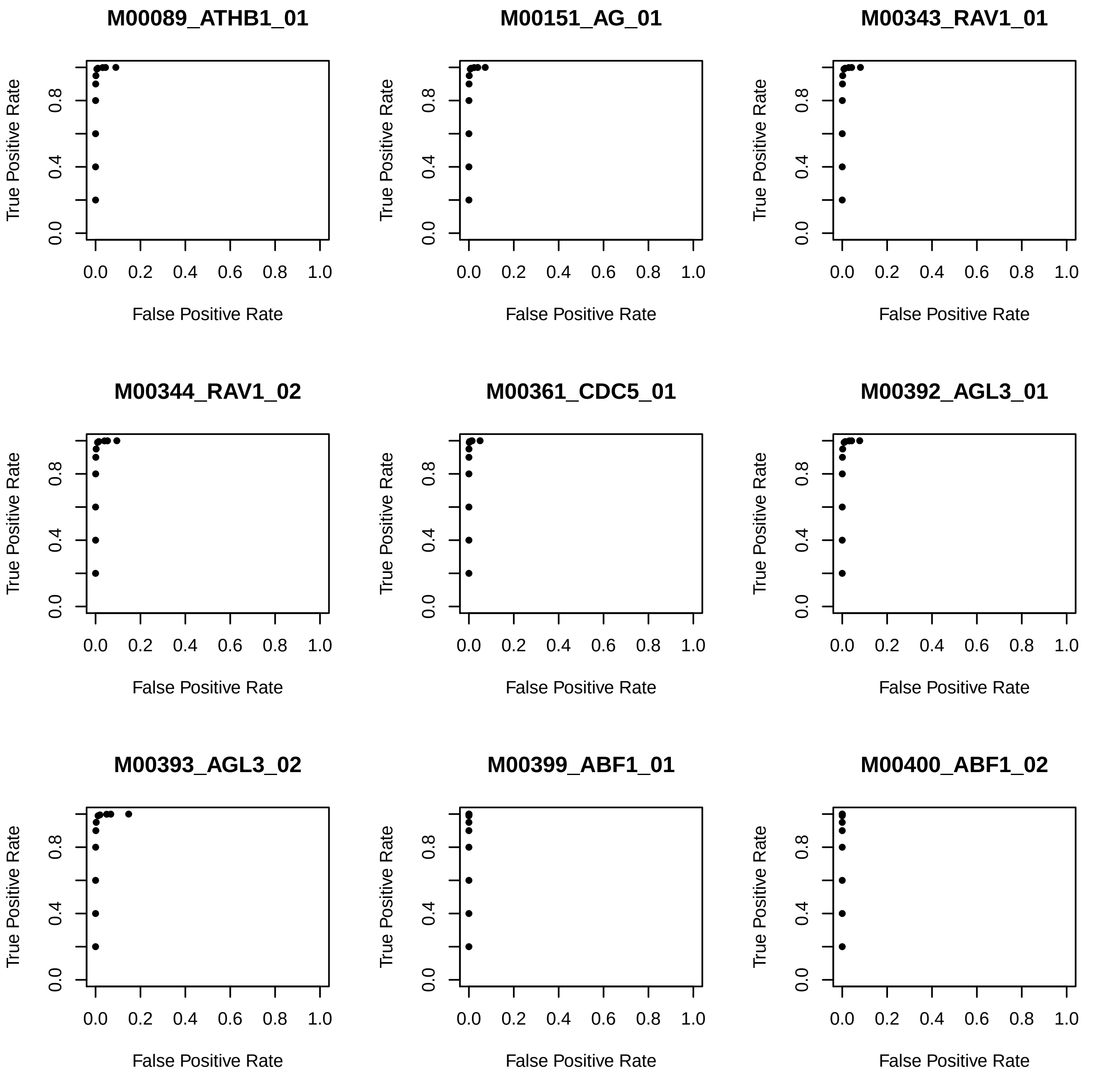
M00089_ATHB1_01
M00151_AG_01
M00343_RAV1_01
M00344_RAV1_02
M00361_CDC5_01
M00392_AGL3_01
M00393_AGL3_02
M00399_ABF1_01
M00400_ABF1_02
True Positive Rate
False Positive Rate
0.0
0.4
0.8
0.0
0.2
0.4
0.6
0.8
1.0

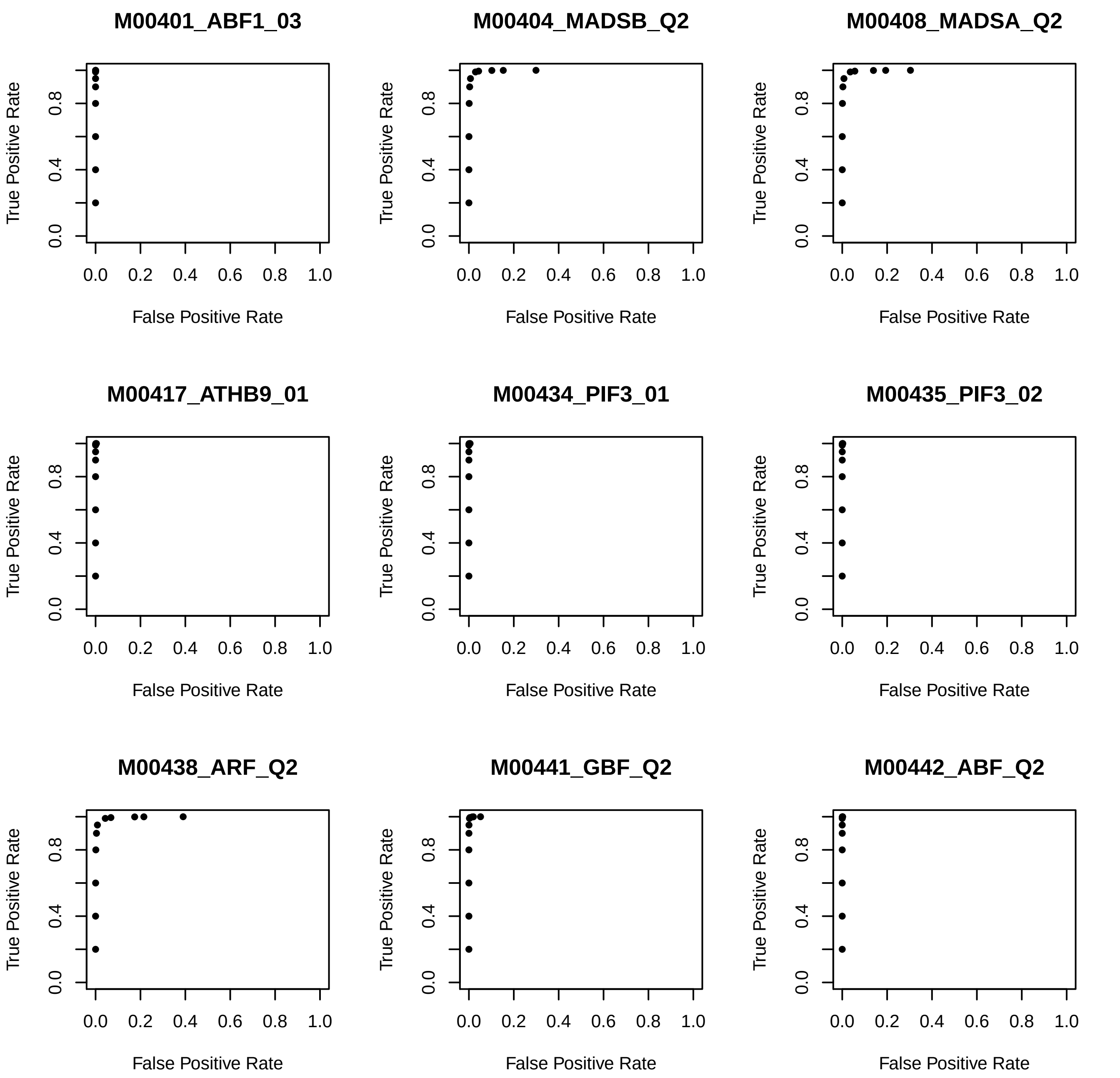
M00401_ABF1_03
M00404_MADSB_Q2
M00408_MADSA_Q2
M00417_ATHB9_01
M00434_PIF3_01
M00435_PIF3_02
M00438_ARF_Q2
M00441_GBF_Q2
M00442_ABF_Q2
True Positive Rate
False Positive Rate
0.0
0.2
0.4
0.6
0.8
1.0

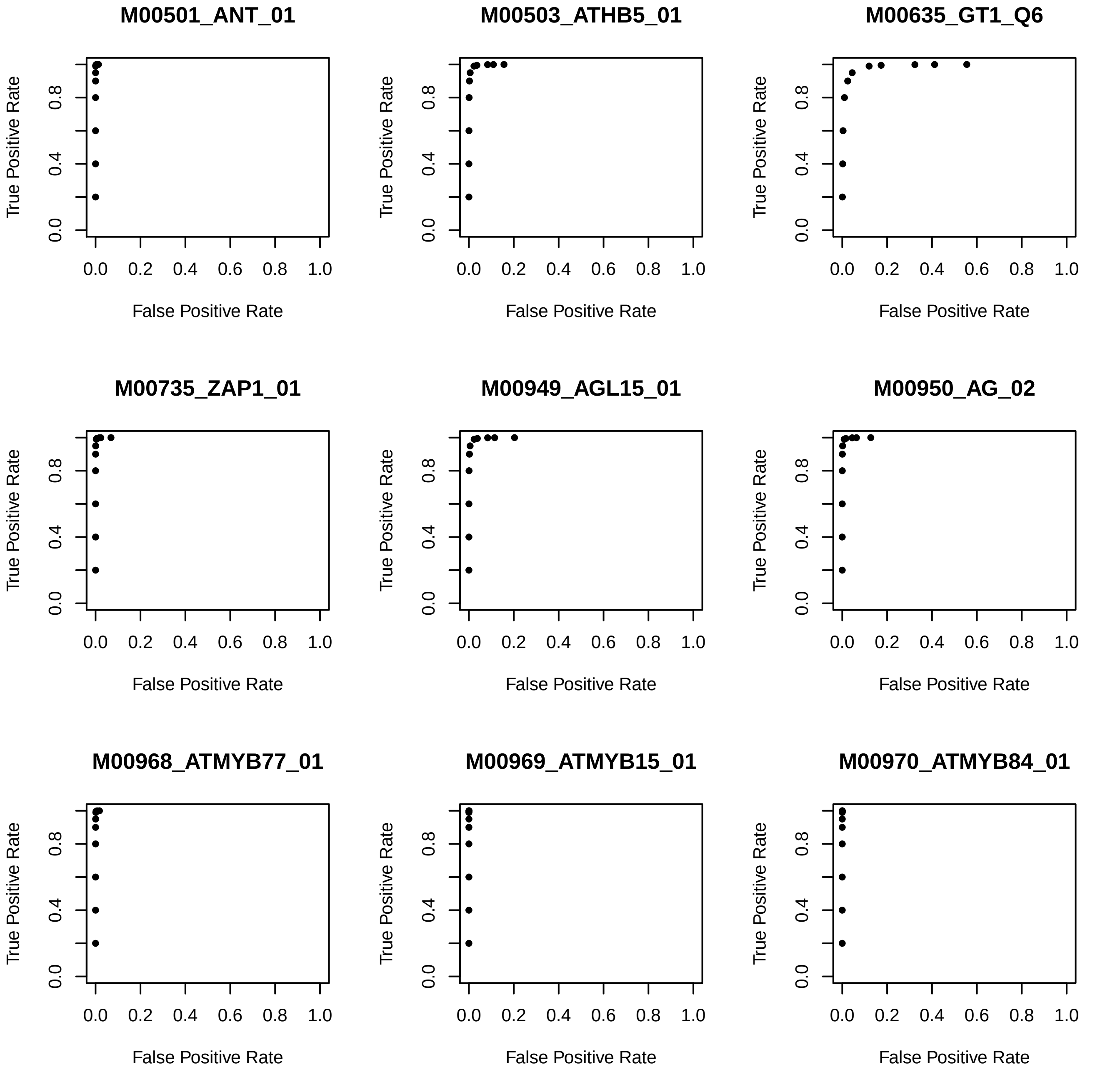
M00501_ANT_01
M00503_ATHB5_01
M00635_GT1_Q6
M00735_ZAP1_01
M00949_AGL15_01
M00950_AG_02
M00968_ATMYB77_01
M00969_ATMYB15_01
M00970_ATMYB84_01
True Positive Rate
False Positive Rate
0.0
0.2
0.4
0.6
0.8
1.0

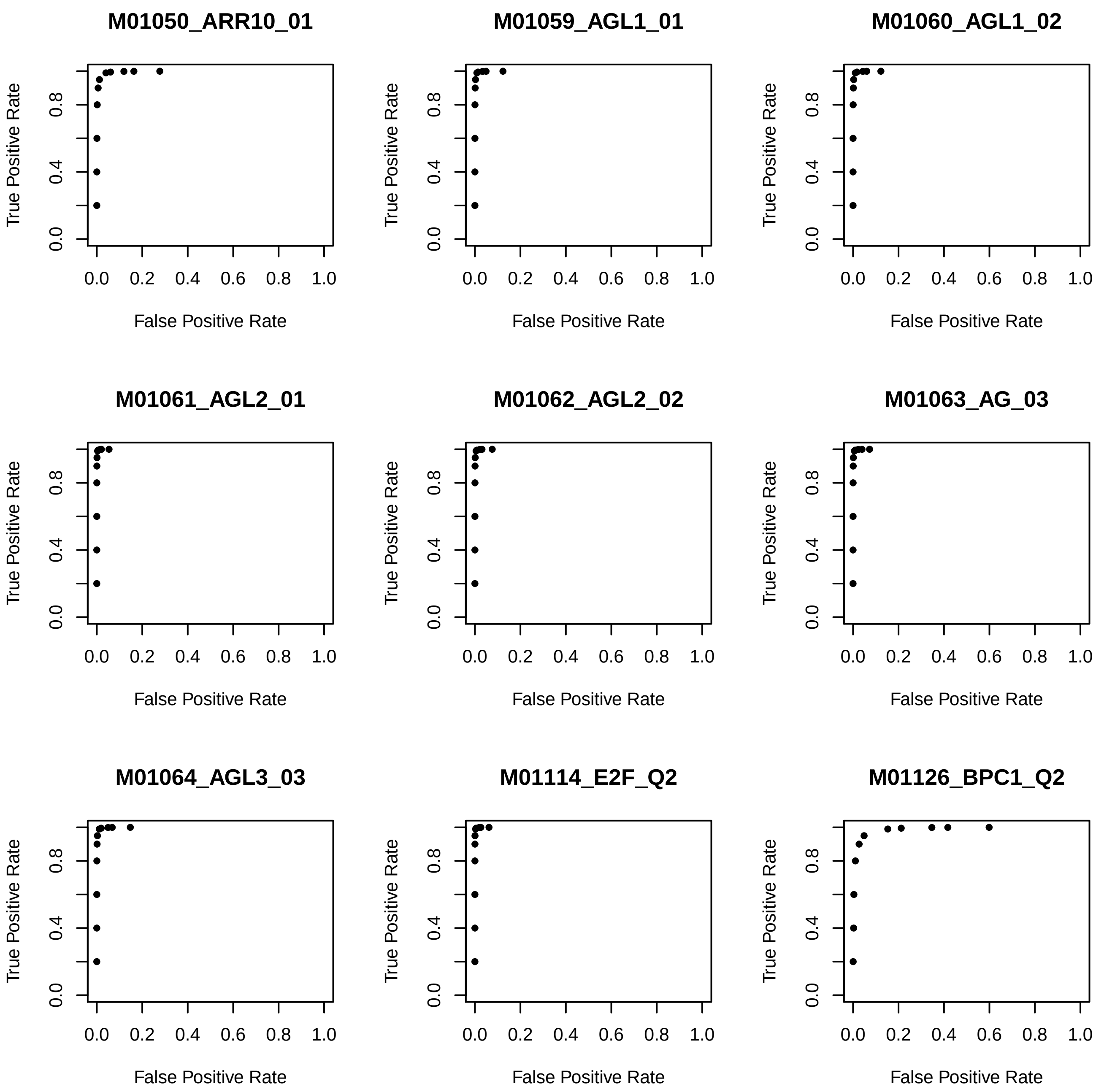

M01050_ARR10_01
M01059_AGL1_01
M01060_AGL1_02
M01061_AGL2_01
M01062_AGL2_02
M01063_AG_03
M01064_AGL3_03
M01114_E2F_Q2
M01126_BPC1_Q2
True Positive Rate
False Positive Rate
0.0
0.2
0.4
0.6
0.8
1.0

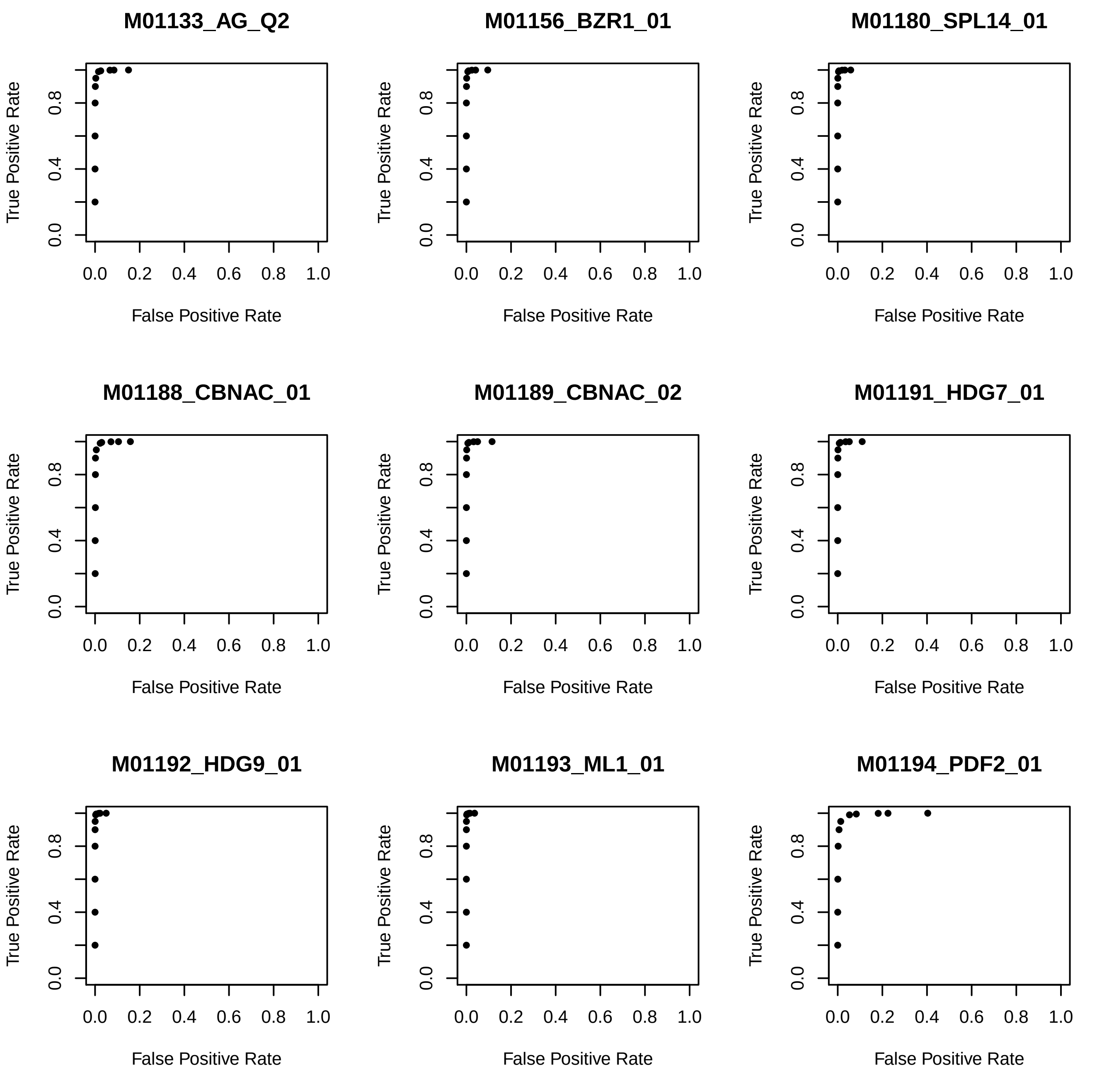

M01133_AG_Q2
M01156_BZR1_01
M01180_SPL14_01
M01188_CBNAC_01
M01189_CBNAC_02
M01191_HDG7_01
M01192_HDG9_01
M01193_ML1_01
M01194_PDF2_01
True Positive Rate
False Positive Rate
0.0
0.4
0.8
0.0
0.2
0.4
0.6
0.8
1.0
