# Supplementary material for: Sustained-input switches for transcription factors and microRNAs are central building blocks of eukaryotic gene circuits
Source: Genome Biol. 2013 Aug 23;14(8):R85. doi: 10.1186/gb-2013-14-8-r85 (PMC4054853; doi:10.1186/gb-2013-14-8-r85)
Supplement: Additional file 9 — PWM ROC Curves, Comprehensive Set. Zipped pdf file containing ROC curve plots for each PWM in the Comprehensive set. [file gb-2013-14-8-r85-S9.ZIP › all_pwms_roc_curves.pdf]

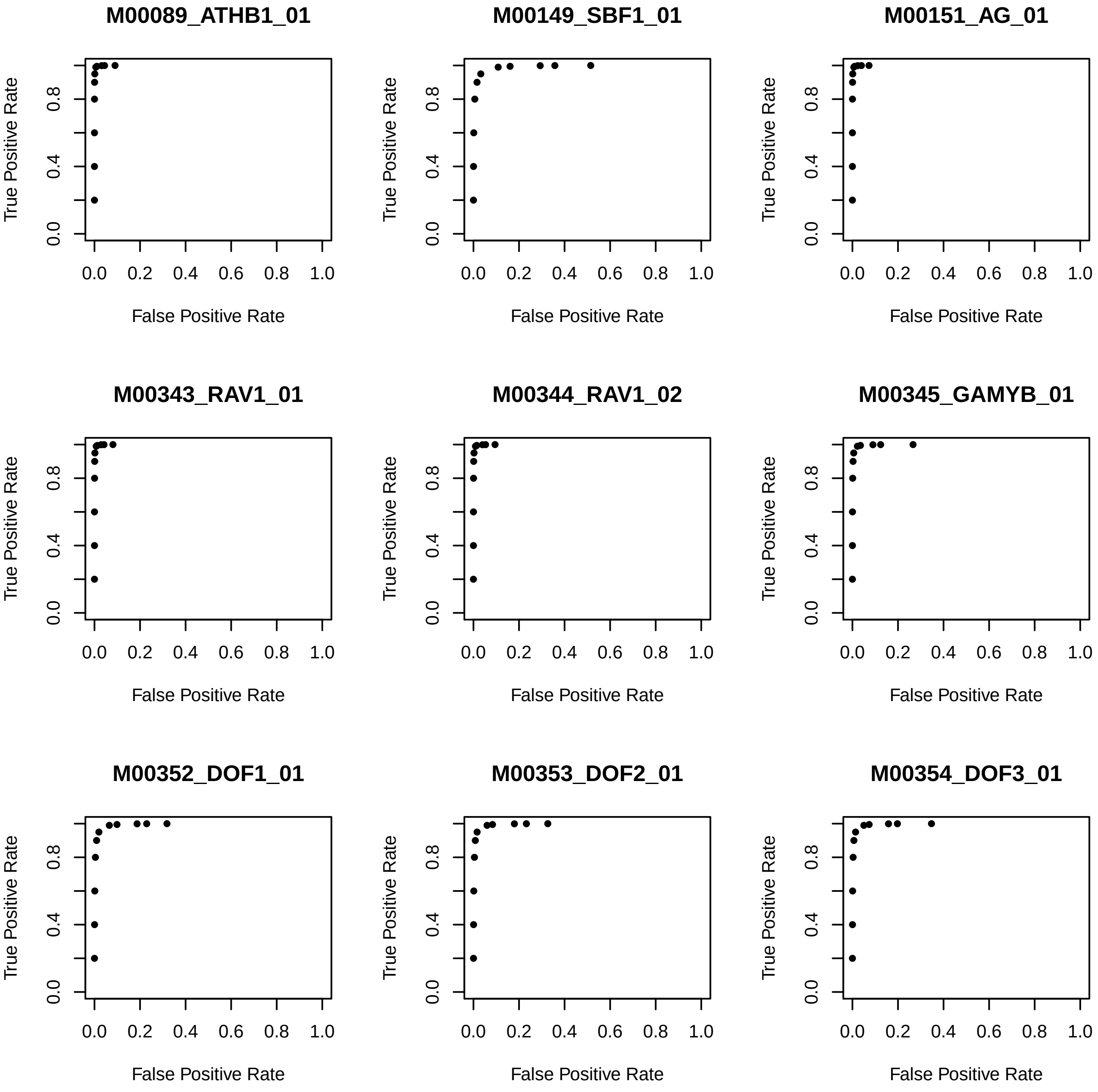
M00089_ATHB1_01
M00149_SBF1_01
M00151_AG_01
M00343_RAV1_01
M00344_RAV1_02
M00345_GAMYB_01
M00352_DOF1_01
M00353_DOF2_01
M00354_DOF3_01
True Positive Rate
False Positive Rate
0.0
0.4
0.8
0.0
0.2
0.4
0.6
0.8
1.0

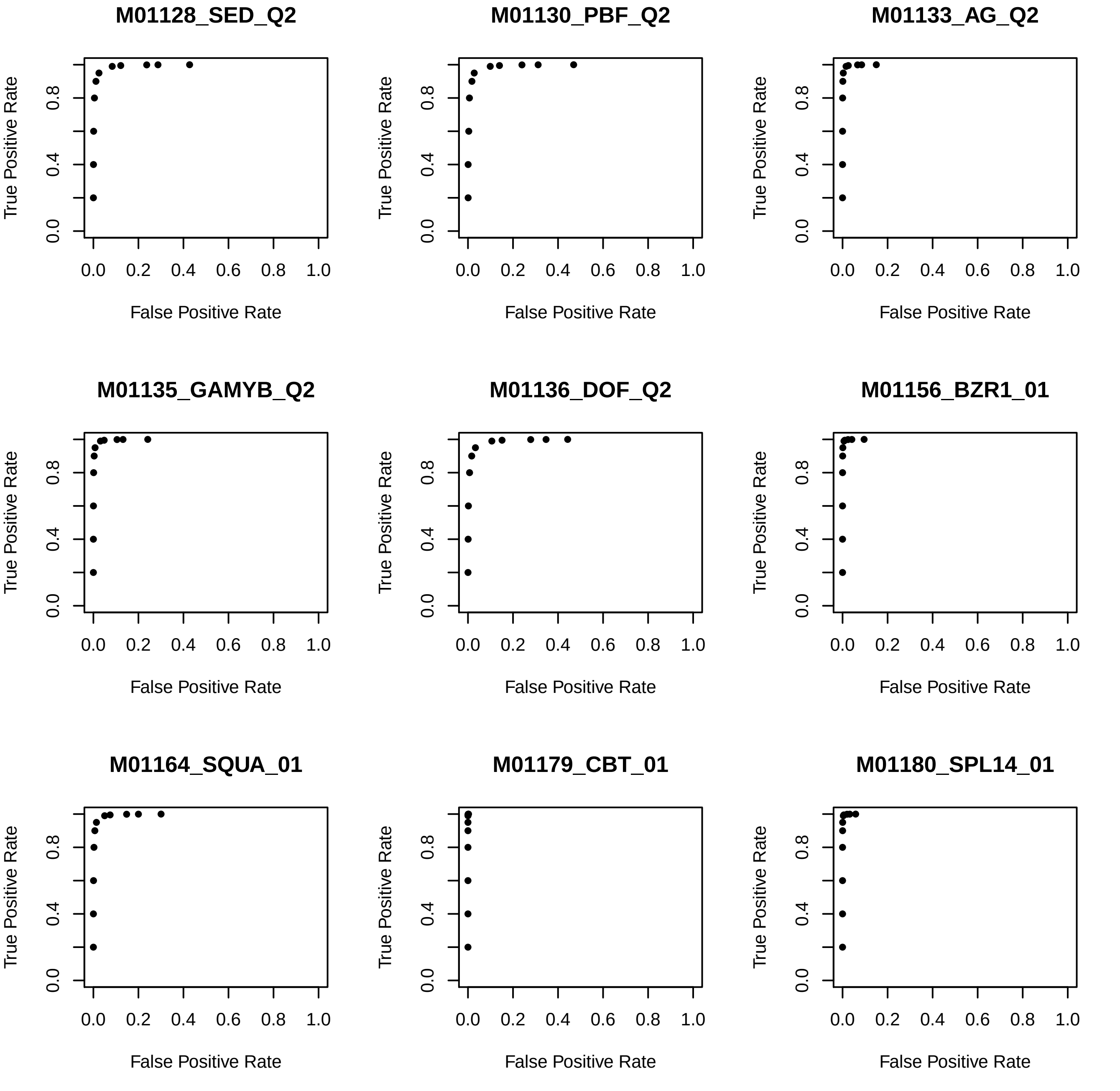
M01128_SED_Q2
M01130_PBF_Q2
M01133_AG_Q2
M01135_GAMYB_Q2
M01136_DOF_Q2
M01156_BZR1_01
M01164_SQUA_01
M01179_CBT_01
M01180_SPL14_01
True Positive Rate
False Positive Rate
0.0
0.2
0.4
0.6
0.8
1.0

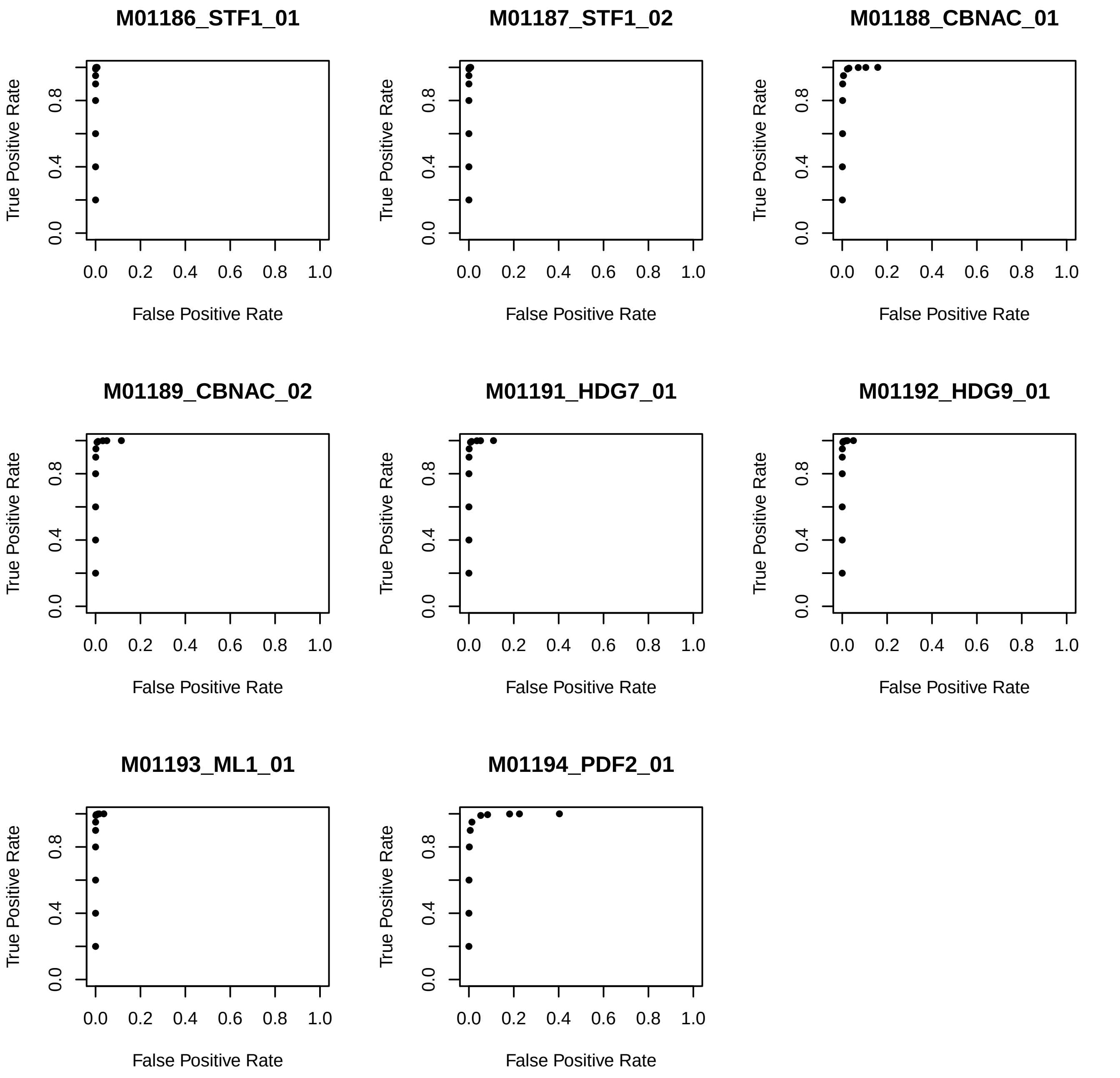
M01186_STF1_01
M01187_STF1_02
M01188_CBNAC_01
M01189_CBNAC_02
M01191_HDG7_01
M01192_HDG9_01
M01193_ML1_01
M01194_PDF2_01
True Positive Rate
False Positive Rate
0.0 0.4 0.8
0.0 0.2 0.4 0.6 0.8 1.0

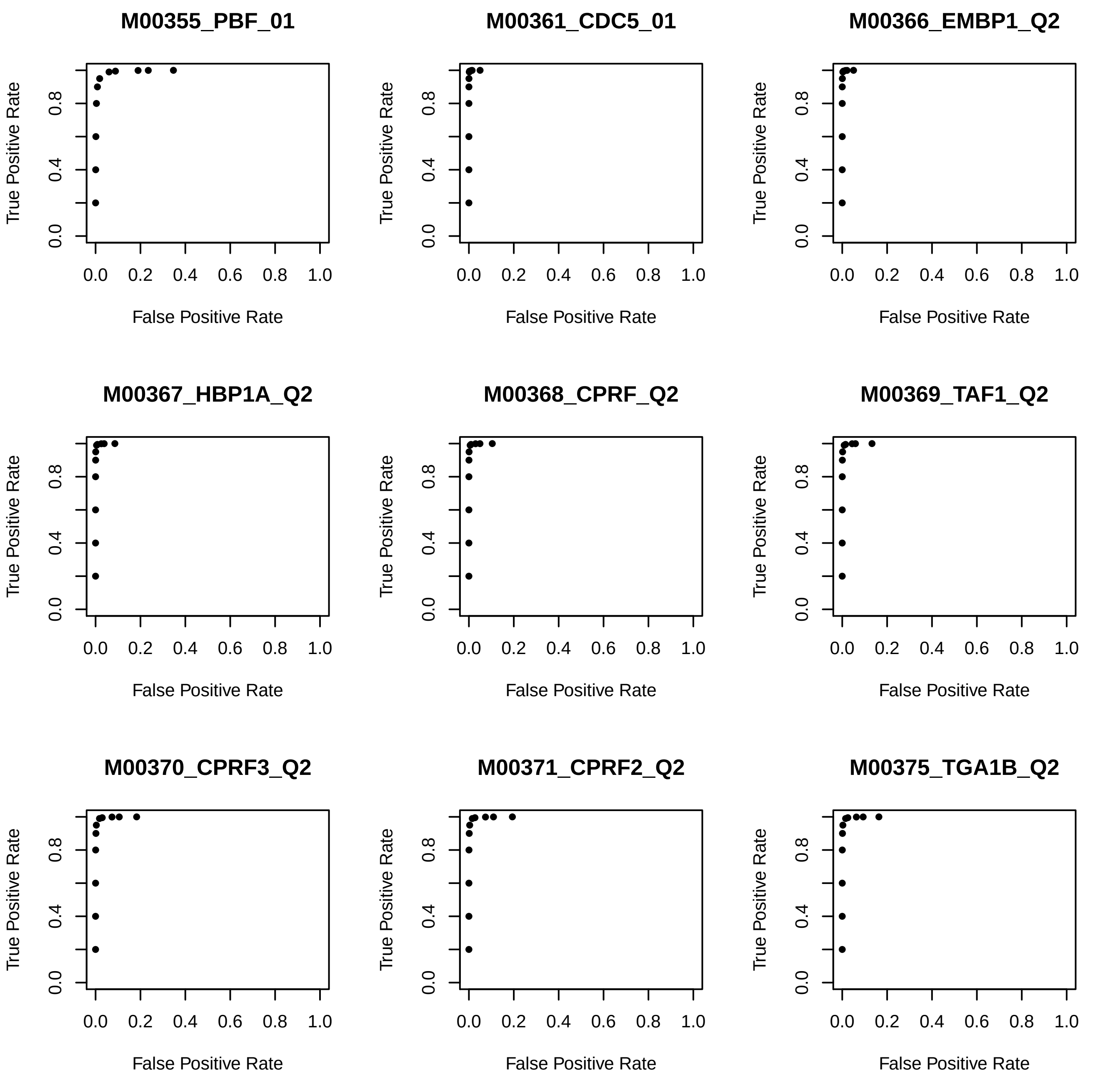
M00355_PBF_01
M00361_CDC5_01
M00366_EMBP1_Q2
M00367_HBP1A_Q2
M00368_CPRF_Q2
M00369_TAF1_Q2
M00370_CPRF3_Q2
M00371_CPRF2_Q2
M00375_TGA1B_Q2
True Positive Rate
False Positive Rate
0.0
0.2
0.4
0.6
0.8
1.0

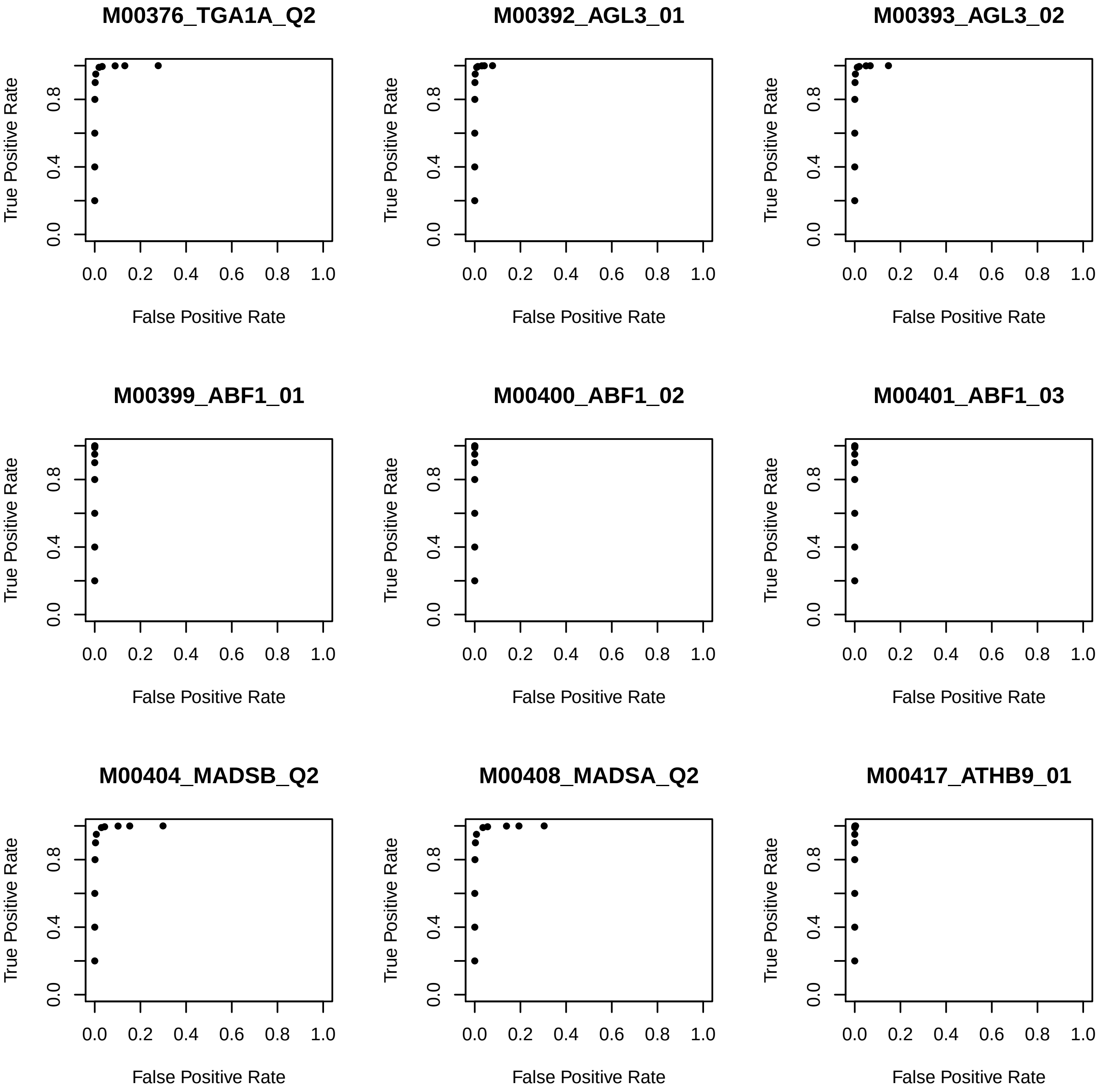
M00376_TGA1A_Q2
M00392_AGL3_01
M00393_AGL3_02
M00399_ABF1_01
M00400_ABF1_02
M00401_ABF1_03
M00404_MADSB_Q2
M00408_MADSA_Q2
M00417_ATHB9_01
True Positive Rate
False Positive Rate
0.0
0.2
0.4
0.6
0.8
1.0

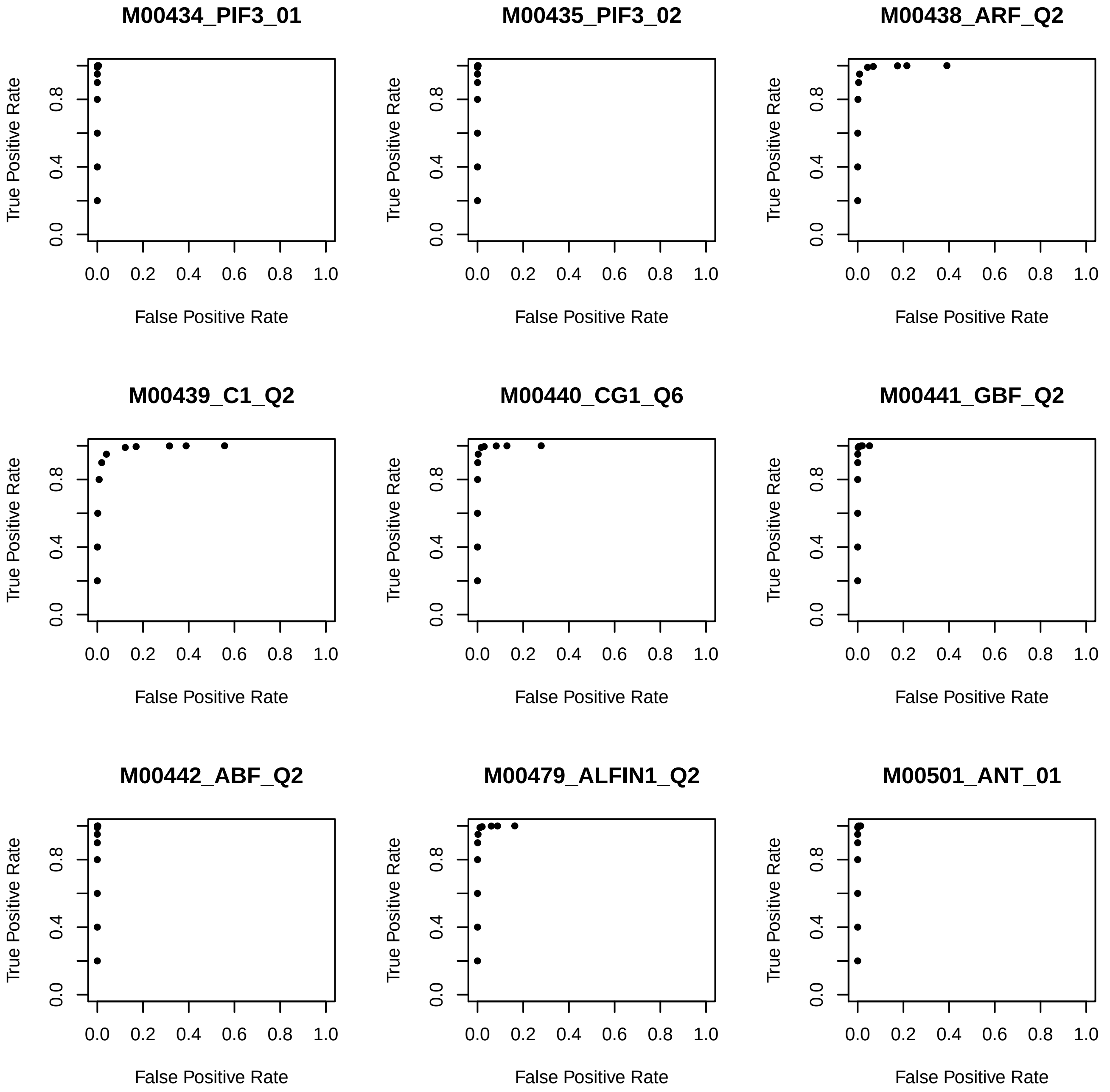
M00434_PIF3_01
M00435_PIF3_02
M00438_ARF_Q2
M00439_C1_Q2
M00440_CG1_Q6
M00441_GBF_Q2
M00442_ABF_Q2
M00479_ALFIN1_Q2
M00501_ANT_01
True Positive Rate
False Positive Rate
0.0
0.2
0.4
0.6
0.8
1.0

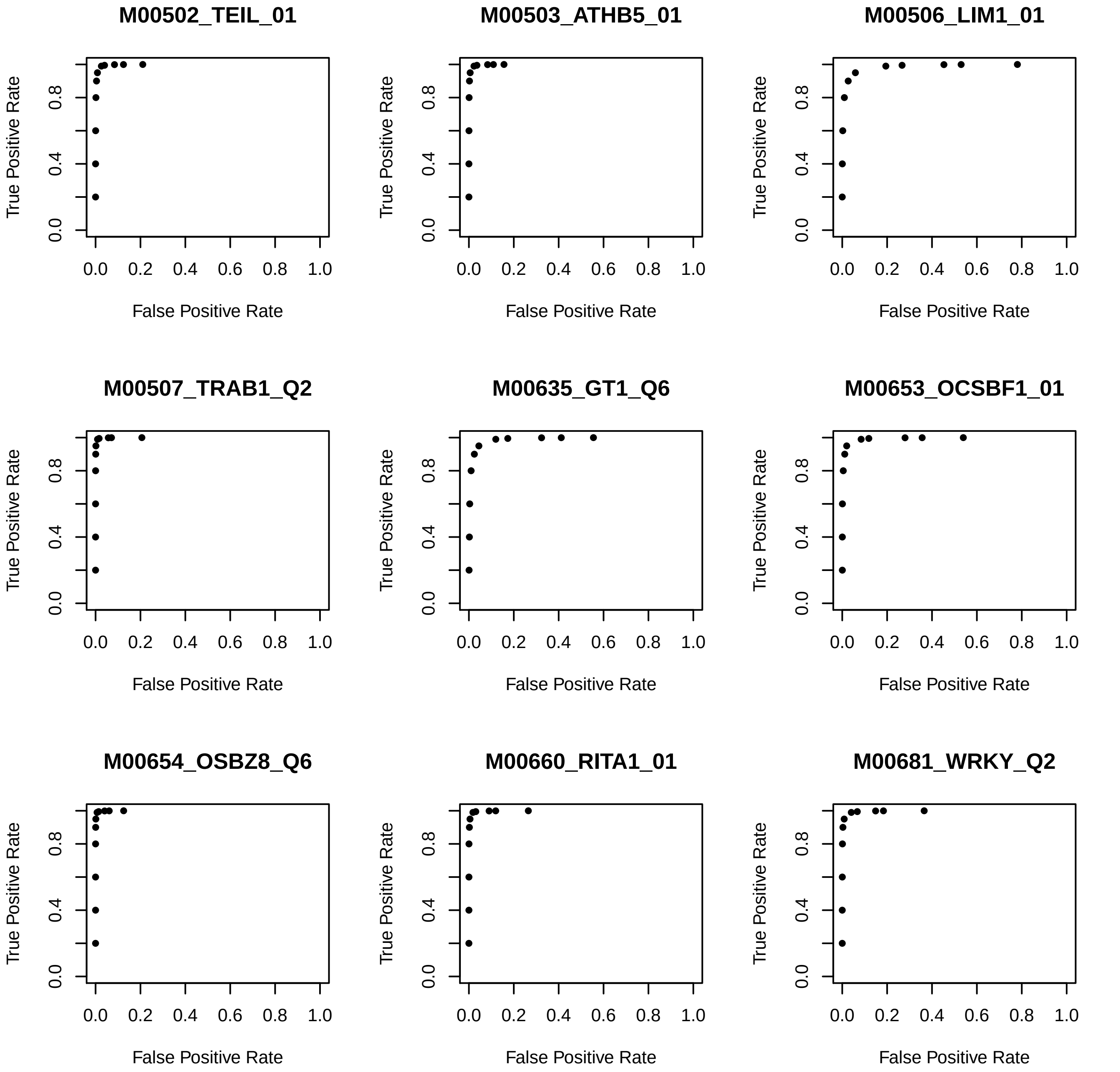
M00502_TEIL_01
M00503_ATHB5_01
M00506_LIM1_01
M00507_TRAB1_Q2
M00635_GT1_Q6
M00653_OCSBF1_01
M00654_OSBZ8_Q6
M00660_RITA1_01
M00681_WRKY_Q2
True Positive Rate
False Positive Rate
0.0
0.2
0.4
0.6
0.8
1.0

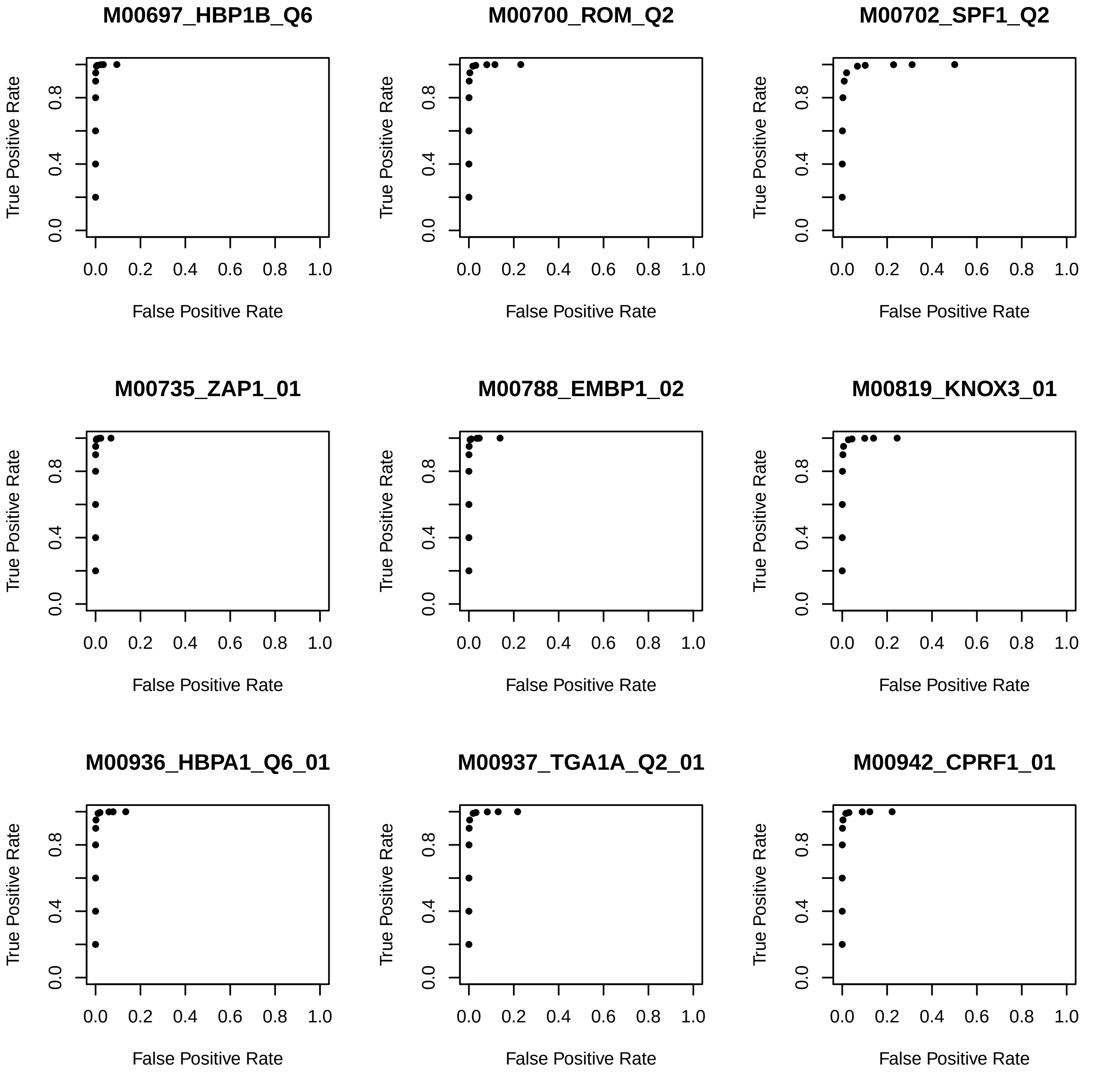
M00697_HBP1B_Q6
M00700_ROM_Q2
M00702_SPF1_Q2
M00735_ZAP1_01
M00788_EMBP1_02
M00819_KNOX3_01
M00936_HBPA1_Q6_01
M00937_TGA1A_Q2_01
M00942_CPRF1_01
True Positive Rate
False Positive Rate
0.0
0.2
0.4
0.6
0.8
1.0

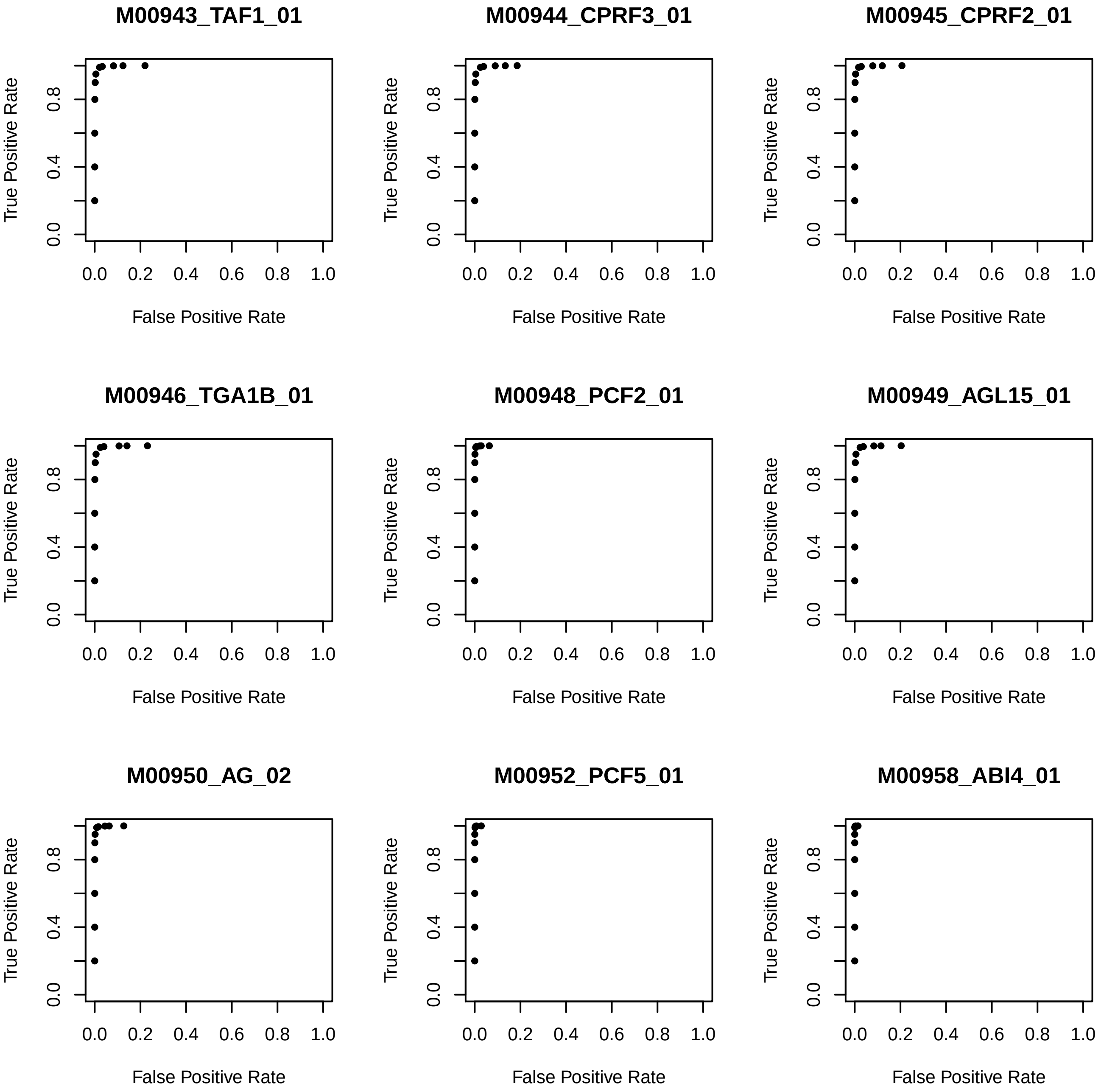
M00943_TAF1_01
M00944_CPRF3_01
M00945_CPRF2_01
M00946_TGA1B_01
M00948_PCF2_01
M00949_AGL15_01
M00950_AG_02
M00952_PCF5_01
M00958_ABI4_01
True Positive Rate
False Positive Rate
0.0
0.2
0.4
0.6
0.8
1.0

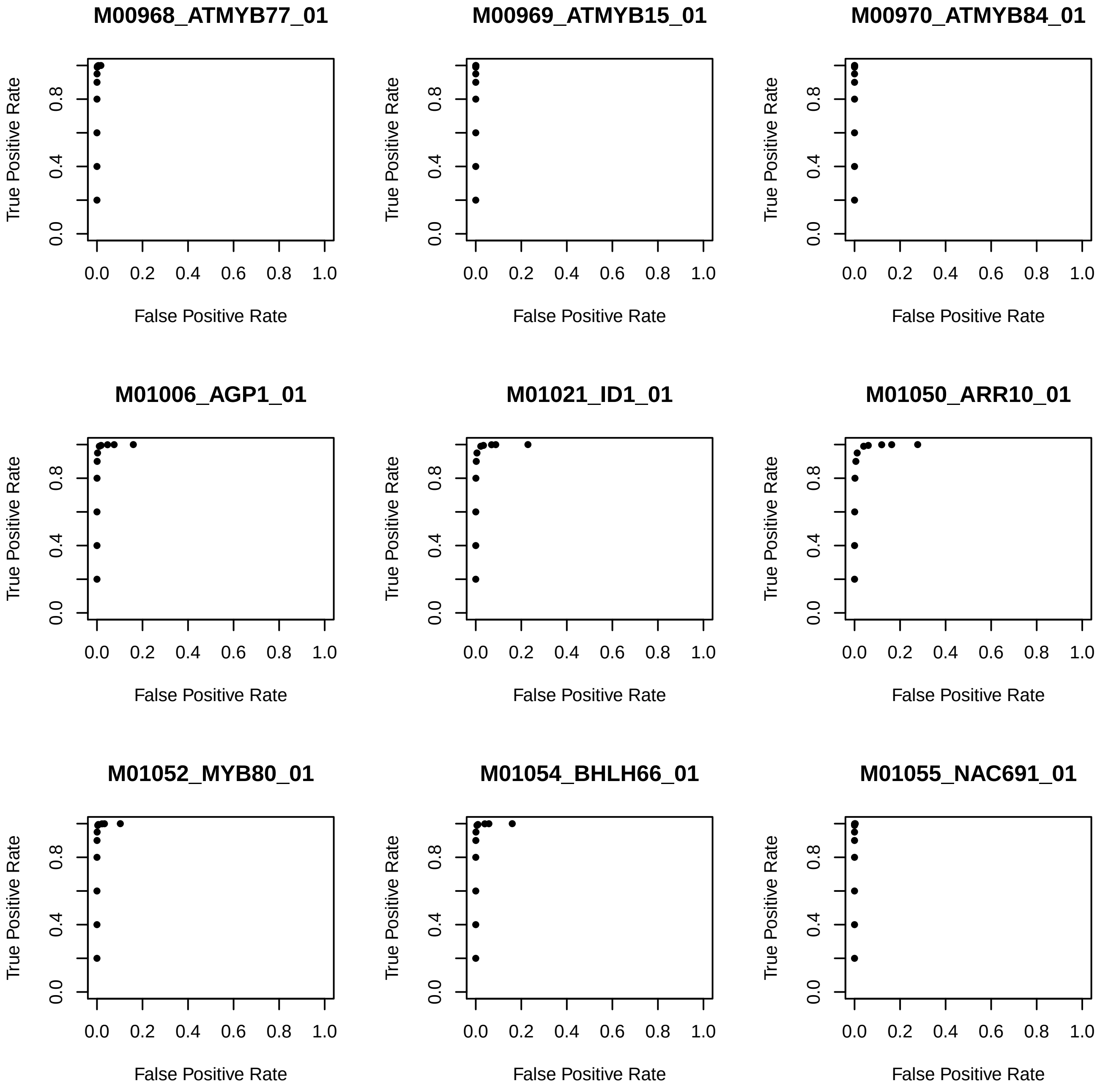
M00968_ATMYB77_01
M00969_ATMYB15_01
M00970_ATMYB84_01
M01006_AGP1_01
M01021_ID1_01
M01050_ARR10_01
M01052_MYB80_01
M01054_BHLH66_01
M01055_NAC691_01
True Positive Rate
False Positive Rate
0.0
0.2
0.4
0.6
0.8
1.0

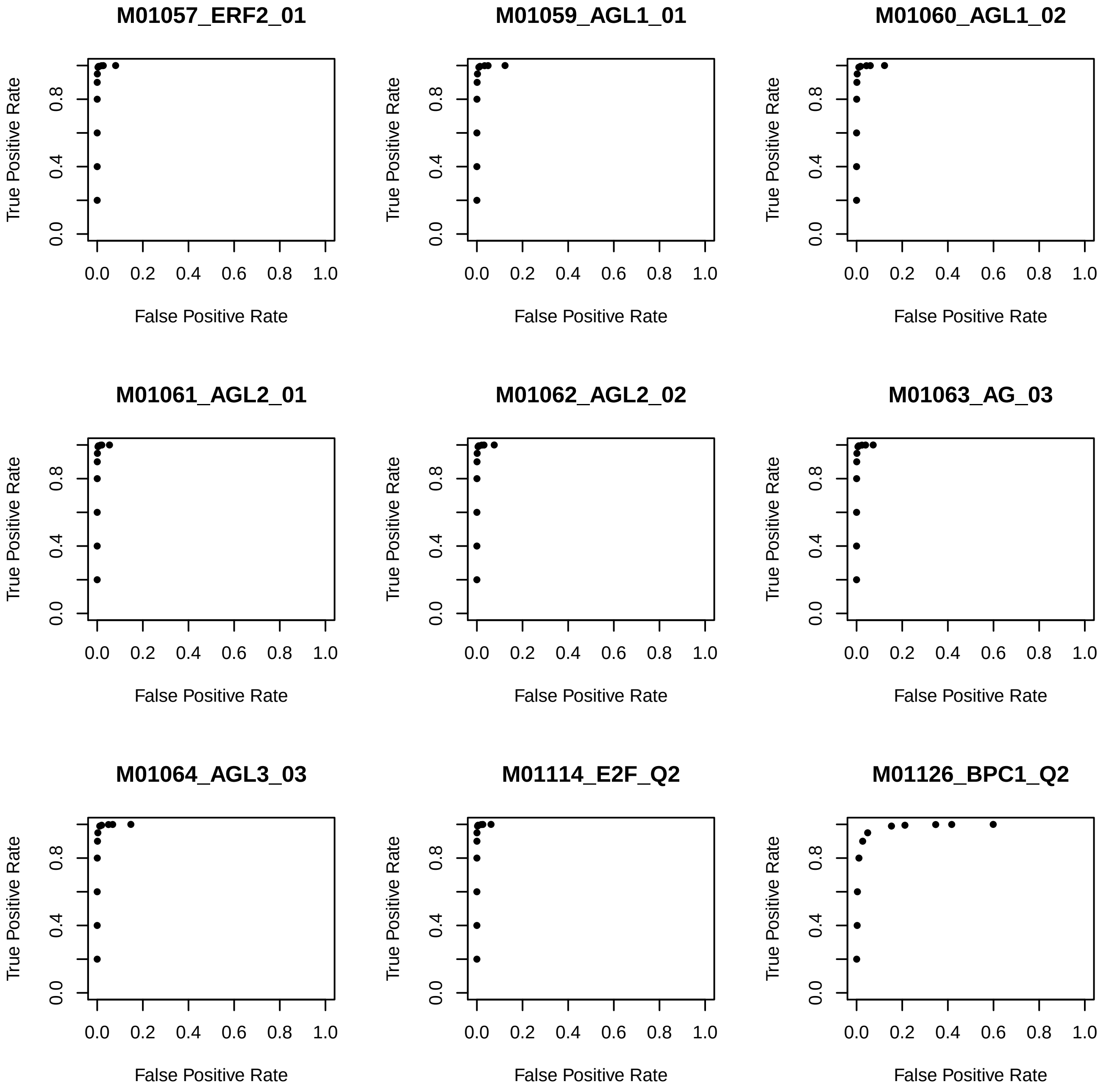
M01057_ERF2_01
M01059_AGL1_01
M01060_AGL1_02
M01061_AGL2_01
M01062_AGL2_02
M01063_AG_03
M01064_AGL3_03
M01114_E2F_Q2
M01126_BPC1_Q2
True Positive Rate
False Positive Rate
0.0
0.2
0.4
0.6
0.8
1.0
